# How Should Parallel Cluster Randomized Trials With a Baseline Period be Analyzed?—A Survey of Estimands and Common Estimators

**DOI:** 10.1002/bimj.70052

**Published:** 2025-04-29

**Authors:** Kenneth Menglin Lee, Fan Li

**Affiliations:** ^1^ Department of Biostatistics, Epidemiology and Informatics University of Pennsylvania Philadelphia Pennsylvania USA; ^2^ Palliative and Advanced Illness Research Center Perelman School of Medicine University of Pennsylvania Philadelphia Pennsylvania USA; ^3^ Department of Biostatistics Yale School of Public Health New Haven Connecticut USA; ^4^ Center for Methods in Implementation and Prevention Science Yale School of Public Health New Haven Connecticut USA

**Keywords:** baseline period, bias and efficiency, cluster randomized trials, estimands, fixed‐effects model, informative cluster sizes

## Abstract

The parallel cluster randomized trial with baseline (PB‐CRT) is a common variant of the standard parallel cluster randomized trial (P‐CRT). We define two natural estimands in the context of PB‐CRTs with informative cluster sizes, the individual‐average treatment effect (iATE) and cluster‐average treatment effect (cATE), to address individual and cluster‐level hypotheses. In this work, we theoretically derive the convergence of the unweighted and inverse cluster‐period size weighted (i) independence estimating equation (IEE), (ii) fixed‐effects (FE) model, (iii) exchangeable mixed‐effects (EME) model, and (iv) nested‐exchangeable mixed‐effects (NEME) model treatment effect estimators in a PB‐CRT with informative cluster sizes and continuous outcomes. Overall, we theoretically show that the unweighted and weighted IEE and FE models yield consistent estimators for the iATE and cATE estimands. Although mixed‐effects models yield inconsistent estimators to these two natural estimands under informative cluster sizes, we empirically demonstrate that the EME model is surprisingly robust to bias. This is in sharp contrast to the corresponding analyses in P‐CRTs and the NEME model in PB‐CRTs when informative cluster sizes are present, carrying implications for practice. We report a simulation study and conclude with a re‐analysis of a PB‐CRT examining the effects of community youth teams on improving mental health among adolescent girls in rural eastern India.

## 1 Introduction

Cluster randomized trials (CRTs) refer to the collection of study designs where randomization is carried out at the group level (such as a hospital, clinic, or worksite level) and often includes outcome measurements collected at the individual level (Turner et al. 2017). The standard parallel cluster randomized trial (P‐CRT) with data collected over a single time period admits two standard estimands with particularly natural interpretations: the cluster‐average treatment effect (cATE, sometimes also referred to as the unit average treatment effect or UATE [Imai et al. 2009]) and the individual‐average treatment effect (iATE, sometimes also referred to as the participant‐average treatment effect or pATE) (Kahan, Li, Copas et al. 2023; Kahan et al. 2024). Briefly, the cATE is the average treatment effect defined at the clusterlevel and is commonly of interest when studying interventions designed for implementation at the cluster level. The iATE is the average treatment effect over individuals across clusters, is often the target estimand had individual randomization been feasible and may often be of relevance when studying individual‐level interventions (which themselves may be cluster randomized as the only feasible randomization scheme). These two estimands correspond to different units of inference and can differ in magnitude when variable cluster sizes moderate treatment effects.

To account for within‐cluster correlation, P‐CRTs are often analyzed with an exchangeable correlation structure specified through a mixed‐effects model or generalized estimating equations (GEEs). When P‐CRTs have variable cluster sizes, inverse cluster size weights can additionally be specified to ensure that all clusters contribute equally regardless of cluster size (Williamson et al. 2003). This is useful in the presence of heterogeneous treatment effects that vary according to cluster size, also referred to as “informative cluster sizes” (Bugni et al. 2024; Kahan, Li, Blette et al. 2023; B. Wang, Park et al. 2024; X. Wang et al. 2022; Williamson et al. 2003). It can then be of interest to combine the specified exchangeable correlation structure with inverse cluster size weights for estimation of the average treatment effect. However, previous work has indicated that specifying both an inverse cluster size weight and an exchangeable correlation structure in the analysis of P‐CRTs can potentially lead to estimation of an uninterpretable treatment effect estimand that is neither the cATE nor iATE under informative cluster sizes and accordingly observe that “two weights make a wrong” (X. Wang et al. 2022).

A common variant of the P‐CRT design is the parallel CRT with a baseline period (PB‐CRT). Such a design maintains parallel randomization of clusters across two sequences, with one sequence of clusters receiving the treatment following the initial baseline period. Accordingly, the PB‐CRT design allows for more analytic options that can make within‐cluster and/or between‐cluster comparisons (Hooper et al. 2018) and can be more efficient than a standard P‐CRT, depending on the cluster size and correlation parameters (Copas and Hooper 2021; Hooper and Copas 2021; Lee and Cheung 2024a). This baseline period also allows for cluster‐period cell sizes to differ between periods within clusters, which can further complicate the analysis and interpretation of results in PB‐CRT designs.

In this article, our goal is to explore if “two weights make a wrong” still holds in PB‐CRT designs with continuous outcomes, cross‐sectional measurements, and variable cluster‐period sizes between clusters. We start by focusing on two natural estimands of interest in Section 2 before introducing standard estimators for PB‐CRTs in Section 3. We then derive the convergence probability limits of the point estimators from the following set of commonly used models for PB‐CRTs (Section 4):

- independence estimating equation (IEE);
- independence estimating equation with inverse cluster‐period size weighting (IEEw);
- fixed‐effects model (FE);
- fixed‐effects model with inverse cluster‐period size weighting (FEw);
- exchangeable mixed‐effects model (EME);
- exchangeable mixed‐effects model with inverse cluster‐period size weighting (EMEw);
- nested‐exchangeable mixed‐effects model (NEME);
- nested‐exchangeable mixed‐effects model with inverse cluster‐period size weighting (NEMEw).

We further characterize the bias of the EMEw and NEMEw estimators for the cATE estimand in Section 5.

Naturally, the IEE, IEEw, and EME, EMEw estimators are included as direct extensions of the previous work by Wang et al. (2022), who found that corresponding estimators can be consistent and inconsistent, respectively, for the iATE and cATE estimands in a P‐CRT with informative cluster sizes (X. Wang et al. 2022). The NEME model is included as a more general form of the EME model that allows the correlation between different outcomes to vary between periods (Girling and Hemming 2016; Hooper et al. 2016). Alternatively, the FE model can also be interpreted as a more general form of the EME model that allows cluster effects to be correlated with other model covariates, therefore controlling for all measured and unmeasured cluster‐level, time‐invariant covariates and confounding (Allison 2005; Lee and Cheung 2024b; Mundlak 1978; Wooldridge 2010). Although the use of FE models has historically been less favored than mixed‐effects models for application to CRTs (Murray 1998), we demonstrate that the treatment effect coefficient from FE models can still target the iATE or cATE estimand. Furthermore, valid inference for FE models can be achieved using a leave‐one‐cluster‐out jackknife variance estimator.

In particular, with an identity link, the treatment effect estimators under GEE and mixed‐effects models are expected to coincide (Gardiner et al. 2009; Hubbard et al. 2010); accordingly, we only focus on the latter under a mixed‐effects setup in this article. Furthermore, we often assume in this article that cluster‐period sizes only vary between clusters but not between periods within clusters, allowing us to define inverse cluster‐period size weights at the cluster level in the style of Williamson et al. (2003) and Wang et al. (2022).

In this article, the overarching goal is to determine which estimators from the collection of commonly used models are theoretically consistent for the iATE and cATE estimands in PB‐CRTs. In addition, we report a simulation study with informative cluster sizes to compare the performance of the different estimators in terms of bias and efficiency with model‐based and jackknife variance estimators (Section 6). We then re‐analyze a real‐world PB‐CRT examining the effects of community youth teams on improving mental health among adolescent girls in rural Eastern India (Section 7) and end with concluding remarks (Section 8).

## 2 Potential Outcomes Framework and Estimands

In this article, we focus on the basic PB‐CRT design with two periods, I clusters, and $K_{ij}$ individuals in each cluster‐period cell under a cross‐sectional sampling scheme. Data from each cluster are collected in the two discrete, equally spaced periods indexed by j=0,1, with period j=0 being the baseline period where all clusters are in the control group and period j=1 being the follow‐up period where I/2 clusters are randomized to the treatment sequence $S_{i}=1$ and receive the treatment. Accordingly, the total study sample size is $n=\sum_{i=1}^{I}\left(K_{i0}+K_{i1}\right)$. We focus on continuous outcomes $Y_{ijk}$ for each individual k in period j of cluster i.

**FIGURE 1:**
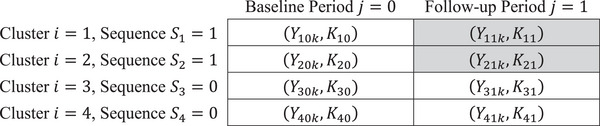
An example of a four‐cluster, two‐period PB‐CRT with continuous outcomes $Y_{ijk}$ and cluster period sizes $K_{ij}$. Cluster‐period cells receiving the treatment are highlighted in gray.

For illustrative purposes, we provide an example of a four‐cluster, two‐period PB‐CRT in Figure 1. This I=4 cluster, two‐period PB‐CRT with continuous outcomes $Y_{ijk}$ has cluster‐period sizes of $K_{ij}$. Half of the clusters are randomized into sequence $S_{i}=1$, where they receive the treatment in period j=1. In Figure 1, cluster‐period cells that receive the treatment are highlighted in gray.

Previous work in stepped‐wedge cluster randomized trials (SW‐CRTs) has defined estimands with potential outcomes by breaking the SW‐CRT design into a “pre‐rollout” baseline period j=0, where all clusters receive the control; “rollout” follow‐up periods j=1,…,J during which some clusters are receiving treatment and some are receiving the control; and a “post‐rollout” period j=J+1 where all clusters receive the treatment (Chen and Li 2024; B. Wang, Wang et al. 2024). Estimands are then defined with potential outcomes during the “rollout” follow‐up periods where there is within‐period variation in treatment.

Analogously, we can use the potential outcomes framework to define the treatment effect estimands in a PB‐CRT during the follow‐up periods, where at least one cluster is exposed under treatment. We denote $Y_{ijk}\left(z\right)$ as the potential outcome for individual k in period j of cluster i receiving treatment arm z=1 (treatment) or 0 (control). Similar to the SW‐CRT, the PB‐CRT by design induces treatment non‐positivity in the baseline period j=0 (Chen and Li 2024; B. Wang, Wang et al. 2024), where potential outcomes under treatment are never observable by the study design and $Y_{i0k}=Y_{i0k}\left(0\right)\;\forall \;i,k$. Therefore, focusing only on the follow‐up period in a PB‐CRT, we connect the observed outcome $Y_{i1k}$ to the potential outcomes via the cluster‐level stable unit treatment value assumption:

$$Y_{i1k}=S_{i}Y_{i1k}\left(1\right)+\left(1-S_{i}\right)Y_{i1k}\left(0\right).$$

To proceed, we pursue a cluster superpopulation framework, where sampled clusters are independent and identically distributed draws from an infinite, notional superpopulation of clusters (Kahan et al. 2024). Under such a framework, randomness is introduced in the sampling of clusters, followed by the randomization. Individuals are assumed to be fixed within each cluster‐period cell, without subsampling within clusters.

Focusing on the follow‐up period, the iATE and cATE can be defined as the average treatment effect across individuals (where individuals are given equal weight) and across clusters (where clusters are given equal weight), respectively (Kahan, Li, Copas et al. 2023). Their mathematical expressions are as follows:

- *individual‐Average Treatment Effect (iATE):*
$$\mathrm{iATE}=E\left[\frac{1}{E\left[K_{i1}\right]}\sum_{k=1}^{K_{i1}}\left[Y_{i1k}\left(1\right)-Y_{i1k}\left(0\right)\right]\right]$$
- *cluster‐Average Treatment Effect (cATE):*
$$\mathrm{cATE}=E\left[\frac{1}{K_{i1}}\sum_{k=1}^{K_{i1}}\left[Y_{i1k}\left(1\right)-Y_{i1k}\left(0\right)\right]\right]$$

with more details included in Supporting Information Appendix Section A.1. Notably, when cluster sizes in the follow‐up period are independent of the potential outcomes (non‐informative cluster sizes) and when potential outcomes have identical marginal means, both the iATE and cATE estimands can be further simplified to $E[Y_{i1k}\left(1\right)-Y_{i1k}\left(0\right)]$ (Supporting Information Appendix Section A.1).

We focus on these two estimands due to their natural interpretations and by no means indicate that these are the only possible estimands of interest in PB‐CRTs. Importantly, these two estimands address different levels of hypothesis. The iATE addresses an individual‐level hypothesis, whereas the cATE addresses a cluster‐level hypothesis; see Kahan, Li, Copas et al. (2023) for a detailed discussion. When all clusters have the same cluster‐period size or there is a homogeneous treatment effect across clusters, the two estimands coincide, but they generally differ in magnitude otherwise. Finally, our estimand definitions are identical to those defined in P‐CRTs without a baseline period (X. Wang et al. 2022) due to the lack of treatment positivity in the baseline period. However, the presence of a baseline period allows for more analytic options. Hence, our central goal and novel contribution is thus to explore whether any of the existing analytic options previously proposed for PB‐CRTs can target these potential outcome estimands in the presence of informative cluster sizes.

## 3 Analytic Models for PB‐CRTs

There are several common analytic models for PB‐CRTs (Hooper et al. 2018). First, PB‐CRTs can be simply analyzed with an “independence estimating equation” (IEE) with treatment and period fixed effects. Such a model strictly makes “vertical” within‐period comparisons; therefore, the baseline period does not contribute any information to the treatment effect estimator. Accordingly, the IEE yields an equivalent estimator to the ordinary least squares (OLS) estimator applied to only the follow‐up period data as if it were a P‐CRT.

Alternatively, we can utilize information from the baseline period to analyze PB‐CRT data using a “two‐way fixed‐effects model,” which includes fixed effects for both the clusters and periods. Throughout, we will simply refer to this as the “fixed‐effects” (FE) model. With a PB‐CRT design, the FE model estimator makes both between‐ and within‐cluster comparisons and closely resembles a standard difference‐in‐differences (DiD) estimator (Goodman‐Bacon 2021). In fact, as we will demonstrate, the FE model with inverse cluster‐period size weights in a PB‐CRT is equivalent to a DiD estimator with cluster‐period cell means. Furthermore, the corresponding FE model in the analysis of SW‐CRTs, another multi‐period CRT design, has been recently illustrated to automatically adjust for all measured and unmeasured cluster‐level time‐invariant covariates and potential confounding (Lee and Cheung 2024b).

Cluster effects in such multi‐period CRT designs can also be accounted for with a mixed‐effects model. Unlike the FE model, mixed‐effects models specify cluster intercepts (among other potential terms) as random effects, which makes an additional exogeneity assumption between the modeled (random) cluster intercepts and the other model covariates and does not automatically adjust for cluster‐level time‐invariant covariates and potential confounding (Wooldridge 2010). Instead, the mixed‐effects model relies on cluster randomization in the study design to avoid confounding and balance covariates. Therefore, a major distinction between modeling clusters as random or fixed depends on whether cluster‐level confounders may exist (Gardiner et al. 2009). In this article, we investigate an “exchangeable mixed‐effects” (EME) model, which specifies a cluster random effect to induce an exchangeable correlation structure. Such a model has been previously referred to as a “constrained baseline analysis with a less flexible correlation structure” in the analysis of PB‐CRTs (Hooper et al. 2018). We also investigate a “nested‐exchangeable mixed‐effects” (NEME) model, which specifies both a cluster random effect and a cluster‐period random interaction to induce a nested‐exchangeable correlation structure between outcomes within the same cluster. Such a model has been previously referred to as a “constrained baseline analysis with a realistic correlation structure” in the analysis of PB‐CRTs (Hooper et al. 2018).

### 3.1 IEE Model

We can analyze the treatment effects in a PB‐CRT by specifying treatment and period fixed effects in an IEE model. As mentioned previously, such a model yields an equivalent estimator to the OLS estimator applied to only the follow‐up period data as if it were a P‐CRT. Accordingly, this estimating equation has an independence correlation structure within and between clusters:

(1) $$\begin{array}{c} Y_{ijk}=\mu +X_{ij}\delta +\Phi_{j}+e_{ijk}\\ e_{ijk}\overset{iid}{∼}N\left(0,\sigma_{w}^{2}\right) \end{array}$$

where μ is the grand mean, $X_{ij}$ and δ are the indicator and coefficient for the treatment effect, $\Phi_{j}$ is the period fixed effect for period j ($\Phi_{0}=0$ for identifiability), and $e_{ijk}$ is the residual error.

The treatment effect in the IEE model can be estimated using OLS, with Equation (1) rewritten as:

(2) $$\begin{array}{c} Y=Z\theta_{\mathrm{IEE}}+\varepsilon \\ \varepsilon \overset{iid}{∼}N\left(0,\dot{V}\right) \end{array}$$

with Y being the n by 1 vector of individual‐level outcomes $Y_{ijk}$. Z is the conventional n by (2+1) design matrix and $\theta_{\mathrm{IEE}}={\left(\mu ,\delta ,\Phi_{1}\right)}^{\prime }$ is the J+1 by 1 vector of parameters. $\dot{V}=\sigma_{w}^{2}I_{n}$ (where $I_{n}$ is an n by n dimension identity matrix) denotes the variance–covariance matrix of Y. Recall that $n=\sum_{i=1}^{I}\sum_{j=0}^{1}K_{ij}$ for the total sample size.

The resulting IEE point estimator is then:

(3) $$\;{\hat{\theta }}_{\mathrm{IEE}}={\left(Z^{\prime }Z\right)}^{-1}Z^{\prime }Y.$$

### 3.2 FE Model

We can define the FE model in the analysis of a PB‐CRT with treatment, period, and cluster fixed effects, as shown in the following equation:

(4) $$\begin{array}{c} \;Y_{ijk}=\mu +X_{ij}\delta +\Phi_{j}+\alpha_{i}+e_{ijk}\\ e_{ijk}\overset{iid}{∼}N\left(0,\sigma_{w}^{2}\right) \end{array}$$

where $\alpha_{i}$ are the I−1 fixed cluster deviations from μ ($\alpha_{1}=0$ for identifiability).

The treatment effect in the FE model can be estimated using OLS, with Equation (4) rewritten as:

(5) $$\begin{array}{c} Y=\overset{ˇ}{Z}\theta_{\mathrm{FE}}+\varepsilon \\ \varepsilon \overset{\mathrm{iid}}{∼}N\left(0,\dot{V}\right). \end{array}$$

This resembles the IEE model (Equation 2) but is instead defined with $\overset{ˇ}{Z}$ as the n by (I+2) design matrix and $\theta_{\mathrm{FE}}={\left(\mu ,\delta ,\Phi_{1},\alpha_{2},\text{…},\alpha_{I}\right)}^{\prime }$ as the (I+2) by 1 vector of coefficients.

The resulting FE point estimator is then:

(6) $${\hat{\theta }}_{\mathrm{FE}}={\left({\overset{ˇ}{Z}}^{\prime }\overset{ˇ}{Z}\right)}^{-1}{\overset{ˇ}{Z}}^{\prime }Y.$$

### 3.3 EME Model

We can define the EME model in the analysis of a PB‐CRT by including an additional cluster random effect in the IEE model (Equation 1), as shown in the following equation:

(7) $$\begin{array}{c} \;Y_{\mathrm{ijk}}=\mu +X_{\mathrm{ij}}\delta +\Phi_{j}+\alpha_{i}+e_{\mathrm{ijk}}\\ \alpha_{i}\overset{\mathrm{iid}}{∼}N\left(0,\tau_{\alpha }^{2}\right)\\ e_{\mathrm{ijk}}\overset{\mathrm{iid}}{∼}N\left(0,\sigma_{w}^{2}\right). \end{array}$$

In contrast to the FE model (Equation 4), here $\alpha_{i}\overset{\mathrm{iid}}{∼}N\left(0,\tau_{\alpha }^{2}\right)$ is defined as the random (instead of fixed) cluster deviations from μ.

The treatment effect in the EME model can be estimated using generalized least squares (GLS), with Equation (7) rewritten as:

(8) $$\begin{array}{c} Y=Z\theta_{\mathrm{EME}}+\tilde{\varepsilon }\\ \tilde{\varepsilon }∼N\left(0,\tilde{V}\right) \end{array}$$

which resembles the IEE model (Equation 2) but is instead defined with $\tilde{V}$ denoting the variance‐covariance matrix of Y as a n by n block diagonal matrix. $\tilde{V}=I_{I}⊗R_{i}^{\mathrm{EME}}$ (where $I_{I}$ is an I by I dimension identity matrix):

$$\;\tilde{V}=\left(\begin{array}{cccc} R_{1}^{\mathrm{EME}} & 0 & \text{…} & 0\\ 0 & R_{i}^{\mathrm{EME}} & \text{…} & 0\\ \vdots & \vdots & \ddots & \vdots \\ 0 & 0 & \text{…} & R_{I}^{\mathrm{EME}} \end{array}\right)$$

and each block $R_{i}^{\mathrm{EME}}$ is a $\sum_{j=0}^{1}K_{ij}$ by $\sum_{j=0}^{1}K_{ij}$ symmetric matrix:

$$\;R_{i}^{\mathrm{EME}}=I_{\sum_{j=0}^{1}K_{\mathrm{ij}}}\sigma_{w}^{2}+J_{\sum_{j=0}^{1}K_{\mathrm{ij}}}\tau_{\alpha }^{2}=\left(\begin{array}{cccc} \sigma_{w}^{2}+\tau_{\alpha }^{2} & \tau_{\alpha }^{2} & \cdots & \tau_{\alpha }^{2}\\ \tau_{\alpha }^{2} & \sigma_{w}^{2}+\tau_{\alpha }^{2} & \cdots & \tau_{\alpha }^{2}\\ \vdots & \vdots & \ddots & \vdots \\ \tau_{\alpha }^{2} & \tau_{\alpha }^{2} & \cdots & \sigma_{w}^{2}+\tau_{\alpha }^{2} \end{array}\right)$$

where $I_{\sum_{j=0}^{1}K_{ij}}$ and $J_{\sum_{j=0}^{1}K_{ij}}$ are a $\sum_{j=0}^{1}K_{ij}$ by $\sum_{j=0}^{1}K_{ij}$ dimension identity matrix and matrix of ones, respectively.

The resulting EME model point estimator is then:

(9) $$\;{\hat{\theta }}_{\mathrm{EME}}={\left(Z^{\prime }{\tilde{V}}^{-1}Z\right)}^{-1}Z^{\prime }{\tilde{V}}^{-1}Y.$$

### 3.4 NEME Model

We can further define the NEME model in the analysis of a PB‐CRT by specifying a cluster random effect and an additional cluster‐period random interaction, as shown in the following equation:

(10) $$\;\begin{array}{c} \begin{array}{c} Y_{\mathrm{ijk}}=\mu +X_{\mathrm{ij}}\delta +\Phi_{j}+\alpha_{i}+\gamma_{\mathrm{ij}}+e_{\mathrm{ijk}}\\ \alpha_{i}\overset{\mathrm{iid}}{∼}N\left(0,\tau_{\alpha }^{2}\right) \end{array}\\ \gamma_{\mathrm{ij}}\overset{\mathrm{iid}}{∼}N\left(0,\tau_{\gamma }^{2}\right)\\ e_{\mathrm{ijk}}\overset{\mathrm{iid}}{∼}N\left(0,\sigma_{w}^{2}\right). \end{array}$$

The NEME model resembles the EME model (Equation 7), but with an additional cluster‐period random interaction term $\gamma_{\mathrm{ij}}∼N\left(0,\tau_{\gamma }^{2}\right)$.

The treatment effect in the NEME model can then be estimated using GLS, with Equation (10) rewritten as:

(11) $$\begin{array}{c} Y=Z\theta_{\mathrm{NEME}}+\overset{ˇ}{\varepsilon }\\ \overset{ˇ}{\varepsilon }∼N\left(0,\overset{ˇ}{V}\right) \end{array}$$

which is defined with $\overset{ˇ}{V}$, denoting the variance–covariance matrix of Y, as an n by n block diagonal matrix. $\overset{ˇ}{V}=I_{I}⊗R_{i}^{\mathrm{NEME}}$ (where $I_{I}$ is an I by I dimension identity matrix):

$$\;\overset{ˇ}{V}=\left(\begin{array}{cccc} R_{1}^{\mathrm{NEME}} & 0 & \cdots & 0\\ 0 & R_{i}^{\mathrm{NEME}} & \cdots & 0\\ \vdots & \vdots & \ddots & \vdots \\ 0 & 0 & \cdots & R_{I}^{\mathrm{NEME}} \end{array}\right)$$

and each block $R_{i}^{\mathrm{NEME}}$ is a $\sum_{j=0}^{1}K_{ij}$ by $\sum_{j=0}^{1}K_{ij}$ symmetric matrix that can be written as the following block matrices:

$$\;R_{i}^{\mathrm{NEME}}=\left(\begin{array}{cc} R_{i1}^{\mathrm{NEME}} & R_{i2}^{\mathrm{NEME}}\\ R_{i3}^{\mathrm{NEME}} & R_{i4}^{\mathrm{NEME}} \end{array}\right).$$

Assuming equal cluster‐period cell sizes between periods within clusters, $K_{i0}=K_{i1}=K_{i-}$, for simplicity, the components of the correlation matrix are:

$$\begin{array}{ccc} \;R_{i1}^{\mathrm{NEME}} & = & R_{i4}^{\mathrm{NEME}}=\left(I_{K_{i-}}\sigma_{w}^{2}+J_{K_{i-}}\left(\tau_{\alpha }^{2}+\tau_{\gamma }^{2}\right)\right)\\ & = & \left(\begin{array}{cccc} \sigma_{w}^{2}+\tau_{\alpha }^{2}+\tau_{\gamma }^{2} & \tau_{\alpha }^{2}+\tau_{\gamma }^{2} & \text{…} & \tau_{\alpha }^{2}+\tau_{\gamma }^{2}\\ \tau_{\alpha }^{2}+\tau_{\gamma }^{2} & \sigma_{w}^{2}+\tau_{\alpha }^{2}+\tau_{\gamma }^{2} & \text{…} & \tau_{\alpha }^{2}+\tau_{\gamma }^{2}\\ \vdots & \vdots & \ddots & \vdots \\ \tau_{\alpha }^{2}+\tau_{\gamma }^{2} & \tau_{\alpha }^{2}+\tau_{\gamma }^{2} & \text{…} & \sigma_{w}^{2}+\tau_{\alpha }^{2}+\tau_{\gamma }^{2} \end{array}\right), \end{array}$$

$$\;R_{i2}^{\mathrm{NEME}}=R_{i3}^{\mathrm{NEME}}=\left(J_{K_{i-}}\tau_{\alpha }^{2}\right)=\left(\begin{array}{cccc} \tau_{\alpha }^{2} & \tau_{\alpha }^{2} & \text{…} & \tau_{\alpha }^{2}\\ \tau_{\alpha }^{2} & \tau_{\alpha }^{2} & \text{…} & \tau_{\alpha }^{2}\\ \vdots & \vdots & \ddots & \vdots \\ \tau_{\alpha }^{2} & \tau_{\alpha }^{2} & \text{…} & \tau_{\alpha }^{2} \end{array}\right)$$

where $I_{K_{i-}}$ and $J_{K_{i-}}$ are a $K_{i-}$ by $K_{i-}$ dimension identity matrix and matrix of ones, respectively.

The resulting NEME model point estimator is then:

(12) $${\hat{\theta }}_{\mathrm{NEME}}={\left(Z^{\prime }{\overset{ˇ}{V}}^{-1}Z\right)}^{-1}Z^{\prime }{\overset{ˇ}{V}}^{-1}Y.$$

## 4 Convergence of Different Estimators

In this section, we derive different estimators and their corresponding convergence probability limits for estimating the iATE and cATE; full derivations are included in Supporting Information Appendix Section A.2. The properties of these estimators are summarized in Table 1. For clarity, we first demonstrate this in the analysis of P‐CRTs before extending the weighting to PB‐CRTs.

**TABLE 1:** The convergence probability limits of the different treatment effect estimators $\hat{\delta }$ described here for continuous outcomes, alongside the sufficient conditions under which each method converges to the iATE or cATE estimands.

Method	$\hat{\delta }\overset{P}{\to }$	Sufficient conditions for convergence to the	
		iATE	cATE
IEE	$E\left[\frac{1}{E[K_{i1}]}\sum_{k=1}^{K_{i1}}\left[Y_{i1k}\left(1\right)-Y_{i1k}\left(0\right)\right]\right]$	Always	No ICS
IEEw	$E\left[\frac{1}{K_{i1}}\sum_{k=1}^{K_{i1}}\left[Y_{i1k}\left(1\right)-Y_{i1k}\left(0\right)\right]\right]$	No ICS	Always
FE	$E\left[\frac{\frac{K_{i0}}{\sum_{j=0}^{1}K_{\mathrm{ij}}}\sum_{k=1}^{K_{i1}}\left[Y_{i1k}\left(1\right)-Y_{i1k}\left(0\right)\right]}{E\left[\frac{\prod_{j=0}^{1}K_{\mathrm{ij}}}{\sum_{j=0}^{1}K_{\mathrm{ij}}}\right]}\right]$	When $K_{i0}=K_{i1}$ or no ICS	No ICS
FEw	$E\left[\frac{1}{K_{i1}}\sum_{k=1}^{K_{i1}}\left[Y_{i1k}\left(1\right)-Y_{i1k}\left(0\right)\right]\right]$	No ICS	Always
EME*	$E\left[\frac{\left(\frac{1+(K_{i-}-1)\rho }{1+(2K_{i-}-1)\rho }\right)}{E\left[\left(\frac{1+(K_{i-}-1)\rho }{1+(2K_{i-}-1)\rho }\right)K_{i-}\right]}\sum_{k=1}^{K_{i-}}\left[Y_{i1k}\left(1\right)-Y_{i1k}\left(0\right)\right]\right]$	When ρ=0 or 1 and $K_{i0}=K_{i1}$, or no ICS	No ICS
EMEw*	$E\left[\frac{\left(\frac{1+(K_{i-}-1)\rho }{1+(2K_{i-}-1)\rho }\right)}{E\left[\left(\frac{1+(K_{i-}-1)\rho }{1+(2K_{i-}-1)\rho }\right)\right]}\left(\frac{1}{K_{i-}}\sum_{k=1}^{K_{i-}}\left[Y_{i1k}\left(1\right)-Y_{i1k}\left(0\right)\right]\right)\right]$	No ICS	When ρ=0 or 1 and $K_{i0}=K_{i1}$, or no ICS
NEME*	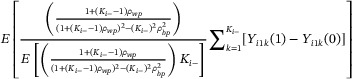	When $\rho_{wp}=\rho_{bp}=0$ and $K_{i0}=K_{i1}$, or no ICS	No ICS
NEMEw*	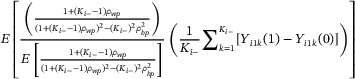	No ICS	When $\rho_{wp}=\rho_{bp}=0$ and $K_{i0}=K_{i1}$, or no ICS

*Note:* Conditions with non‐informative cluster sizes (referred to as “No ICS” and additionally assuming potential outcomes have identical marginal means) occur when 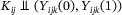, including scenarios where $K_{ij}=K\;\forall \;i,j$ or with homogeneous treatment effects. The ICC is denoted in the EME and EMEw models with $\rho =\frac{\tau_{\alpha }^{2}}{\tau_{\alpha }^{2}+\sigma_{w}^{2}}$. The within‐period ICC and between‐period ICC in the NEME and NEMEw models are accordingly $\rho_{wp}=\frac{\tau_{\alpha }^{2}+\tau_{\gamma }^{2}}{\tau_{\alpha }^{2}+\tau_{\gamma }^{2}+\sigma_{w}^{2}}$ and $\rho_{bp}=\frac{\tau_{\alpha }^{2}}{\tau_{\alpha }^{2}+\tau_{\gamma }^{2}+\sigma_{w}^{2}}$. (*We assume that cluster‐period cell sizes do not vary within clusters $K_{i0}=K_{i1}=K_{i-}$ in the derivation of these estimators.).

Abbreviations: cATE, cluster‐average treatment effect; EME, exchangeable mixed‐effects; EMEw, exchangeable mixed‐effects model with inverse cluster‐period size weighting; FE, fixed‐effects; FEw, fixed‐effects model with inverse cluster‐period size weighting; iATE, individual‐average treatment effect; ICS, informative cluster sizes; IEE, independence estimating equation; IEEw, independence estimating equation with inverse cluster‐period size weighting; NEME, nested‐exchangeable mixed‐effects; NEMEw, nested‐exchangeable mixed‐effects model with inverse cluster‐period size weighting.

In a simple P‐CRT, the cATE estimand can be estimated by additionally utilizing inverse cluster size weights. The weighted treatment effect estimator, as described in Williamson et al. (2003), can be obtained by solving the weighted estimating equations:

$$\sum_{i=1}^{I}\frac{D_{i}^{\prime }V_{i}^{-1}\left(Y_{i}-\mu_{i}\right)}{w_{i}}=0$$

where $D_{i}=\frac{d\mu_{i}}{d\theta }$ and $V_{i}$ is the variance–covariance matrix among outcomes in cluster i, corresponding to the specified model. Subsequently, $Y_{i}={\left(Y_{i1},\cdots ,Y_{iK_{i}}\right)}^{\prime }$ and $\mu_{i}={\left(\mu_{i},\cdots ,\mu_{i}\right)}^{\prime }$ are the $K_{i}$ by 1 vector of outcomes and marginal means $\mu_{i}=E\;\left[Y_{\mathrm{ik}}|Z_{i}\right]=Z_{i}\theta$, respectively. Here, $Z_{i}$ describes the P‐CRT design matrix for observations in cluster i. Subsequently, $w_{i}$ is the cluster i‐specific weight equal to 1 for equal individual‐level weights or cluster size $K_{i}$ for equal cluster‐level weights. This weighted estimating equation can be rewritten as solving for:

$$\sum_{i=1}^{I}D_{i}^{\prime }W_{i}^{-1}\left(Y_{i}-\mu_{i}\right)=0$$

where $W_{i}=I_{I}⊗Q_{i}$ (where $I_{I}$ is an I by I dimension identity matrix) for I total clusters. With an independence and exchangeable correlation structure, we define the model‐specific values of $Q_{i}$ as:

$$\;Q_{i}^{\mathrm{IEE}}=w_{i}\left[\left(\sigma_{w}^{2}\right)I_{K_{i}}\right],$$

$$\;Q_{i}^{\mathrm{EME}}=w_{i}\left[\left(\sigma_{w}^{2}\right)I_{K_{i}}+\left(\tau_{\alpha }^{2}\right)J_{K_{i}}\right],$$

respectively, where $I_{K_{i}}$ is the $K_{i}$ by $K_{i}$ identity matrix and $J_{K_{i}}$ is the $K_{i}$ by $K_{i}$ matrix of ones. Recall that $\sigma_{w}^{2}$ and $\tau_{\alpha }^{2}$ are the residual and cluster random effect variances. Altogether, in a P‐CRT, the vector of coefficients is estimated by:


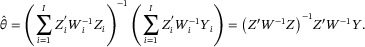




Accordingly, we can extend this weighted treatment effect estimator to PB‐CRTs using inverse cluster‐period size weights to target the cATE. In realistic scenarios where cluster‐period cell sizes vary between periods within a cluster, similar weighting can be implemented in analyses with uncorrelated errors (IEEw and FEw) by performing the corresponding analyses with cluster‐period cell means (as cluster‐level summaries) (Kahan et al. 2024). However, to our knowledge, the extension of variable cluster‐period size weighting to analyses with correlated errors (EMEw and NEMEw) is not as clear in the existing literature, nor is it obvious if such weighted mixed‐effects analyses are even desirable.

We assume for simplicity that cluster‐period sizes vary between clusters but not between periods within clusters in a PB‐CRT, $K_{i0}=K_{i1}=K_{i-}$. Therefore, the weighted treatment effect estimator in a PB‐CRT can still be obtained by solving for:

$$\sum_{i=1}^{I}\;\frac{D_{i}^{\prime }V_{i}^{-1}\left(Y_{i}-\mu_{i}\right)}{w_{i}}=0$$

where $D_{i}=\frac{d\mu_{i}}{d\theta }$ and $V_{i}$ is the variance–covariance matrix among outcomes across periods j=0 and 1 in cluster i, corresponding to the specified model. Subsequently, $Y_{i}={\left(Y_{i01},\cdots ,Y_{i0K_{i-}},Y_{i11},\cdots ,Y_{i1K_{i-}}\right)}^{\prime }$ and $\mu_{i}={\left(\mu_{i0},\cdots ,\mu_{i0},\mu_{i1},\cdots ,\mu_{i1}\right)}^{\prime }$ are the $\left(2K_{i-}\right)$ by 1 vector of outcomes and marginal means, respectively, with $\mu_{i}=E\;\left[Y_{\mathrm{ijk}}|Z_{i}\right]=Z_{i}\theta$ ($\overset{ˇ}{Z}$ in a FE model). The quantity $w_{i}$ is the cluster‐specific weight equal to 1 for equal individual‐level weights or cluster‐period size $K_{i-}$. As in a P‐CRT, this can be rewritten in a PB‐CRT as solving for:

$$\sum_{i=1}^{I}D_{i}^{\prime }W_{i}^{-1}\left(Y_{i}-\mu_{i}\right)=0$$

where $W_{i}=I_{I}⊗Q_{i}$ (where $I_{I}$ is an I by I dimension identity matrix) for I total clusters. With an IEE, FE, EME, and NEME model, we define the corresponding model‐specific values of $Q_{i}$ as:

$$\begin{array}{ccc} & & Q_{i}^{\mathrm{IEE}}=Q_{i}^{\mathrm{FE}}=w_{i}\sigma_{w}^{2},\\ & & Q_{i}^{\mathrm{EME}}=w_{i}R_{i}^{\mathrm{EME}},\\ & & Q_{i}^{\mathrm{NEME}}=w_{i}R_{i}^{\mathrm{NEME}}, \end{array}$$

respectively. Altogether, in a PB‐CRT:

$$\;\hat{\theta }={\left(\sum_{i=1}^{I}Z_{i}^{\prime }W_{i}^{-1}Z_{i}\right)}^{-1}\left(\sum_{i=1}^{I}Z_{i}^{\prime }W_{i}^{-1}Y_{i}\right)={\left(Z^{\prime }W^{-1}Z\right)}^{-1}Z^{\prime }W^{-1}Y$$

($\overset{ˇ}{Z}$ in a FE model).

### 4.1 Independence Estimating Equation (IEE) Estimator

Earlier, we mentioned how the IEE treatment effect estimator only uses information from the follow‐up period of the PB‐CRT and can be written with potential outcomes $\left(Y_{i1k}\left(0\right),Y_{i1k}\left(1\right)\right)$ for individual $k\in (1,\text{…},K_{i1})$ in period j=1 of cluster i∈(1,…,I). Let $S_{i}$ be an indicator for whether individuals are assigned to cluster sequence $S_{i}=1$ (Section 2), the IEE treatment effect estimator is then:

(13) $$\begin{array}{c} \;{\hat{\delta }}_{\mathrm{IEE}}=\left[\frac{\sum_{i=1}^{I}S_{i}\sum_{k=1}^{K_{i1}}Y_{i1k}\left(1\right)}{\sum_{i=1}^{I}S_{i}K_{i1}}\right]\;-\left[\frac{\sum_{i=1}^{I}\left(1-S_{i}\right)\sum_{k=1}^{K_{i1}}Y_{i1k}\left(0\right)}{\sum_{i=1}^{I}\left(1-S_{i}\right)K_{i1}}\right]. \end{array}$$

We can demonstrate that this estimator is consistent and asymptotically unbiased for the iATE:

(14) $${\hat{\delta }}_{\mathrm{IEE}}\overset{P}{\to }E\left[\frac{1}{E\left[K_{i1}\right]}\sum_{k=1}^{K_{i1}}\left[Y_{i1k}\left(1\right)-Y_{i1k}\left(0\right)\right]\right].$$

More information about the derivation of these estimators is included in Supporting Information Appendix Section A.2.1.

### 4.2 Independence Estimating Equation With Inverse Cluster‐Period Size Weighting (IEEw) Estimator

The IEEw treatment effect estimator can then be written as:

$$\begin{array}{ccc} \;{\hat{\delta }}_{\mathrm{IEEw}} & = & \left[\frac{\sum_{i=1}^{I}S_{i}\frac{1}{K_{i1}}\sum_{k=1}^{K_{i1}}Y_{i1k}\left(1\right)}{\sum_{i=1}^{I}S_{i}}\right]-\left[\frac{\sum_{i=1}^{I}\left(1-S_{i}\right)\frac{1}{K_{i1}}\sum_{k=1}^{K_{i1}}Y_{i1k}\left(0\right)}{\sum_{i=1}^{I}\left(1-S_{i}\right)}\right] \end{array}$$

We can demonstrate that this estimator is consistent and asymptotically unbiased for the cATE:

(15) $${\hat{\delta }}_{\mathrm{IEEw}}\overset{P}{\to }E\left[\frac{1}{K_{i1}}\sum_{k=1}^{K_{i1}}\left[Y_{i1k}\left(1\right)-Y_{i1k}\left(0\right)\right]\right].$$

Additionally, the IEEw estimator is also unbiased for the cATE in expectation over the sampling distribution (Supporting Information Appendix Section A.2.2). This is a stronger result than consistency.

### 4.3 Fixed‐Effects Model (FE) Estimator

The FE treatment effect estimator can be written with potential outcomes $\left(Y_{ijk}\left(0\right),Y_{i1k}\left(1\right)\right)$ for individual $k\in (1,\text{…},K_{ij})$ in period j∈(0,1) of cluster i∈(1,…,I). Let $S_{i}$ be an indicator for whether individuals are assigned to cluster sequence $S_{i}=1$ (Section 2):

(16) δ^FE=∑i=1ISiKi0∑j=01Kij∑k=1Ki1Yi1k1−∑i=1ISiKi1∑j=01Kij∑kYi0k0∑i=1ISi∏j=01Kij∑j=01Kij−∑i=1I1−SiKi0∑j=01Kij∑kYi1k0−∑i=1I1−SiKi1∑j=01Kij∑kYi0k0∑i=1I1−Si∏j=01Kij∑j=01Kij.

Accordingly, we have the following convergence in probability result:

(17) $${\hat{\delta }}_{\mathrm{FE}}\overset{P}{\to }E\left[\frac{\frac{K_{i0}}{\sum_{j=0}^{1}K_{ij}}\sum_{k=1}^{K_{i1}}\left[Y_{i1k}\left(1\right)-Y_{i1k}\left(0\right)\right]}{E\left[\frac{\prod_{j=0}^{1}K_{ij}}{\sum_{j=0}^{1}K_{ij}}\right]}\right]$$

when cluster‐period sizes vary between periods within clusters, $K_{i0}\neq K_{i1}$. Notably, this FE estimator is not a theoretically consistent estimator for the iATE estimand when cluster‐period sizes vary within clusters. Additional information is included in Supporting Information Appendix Section A.2.3.

However, if we assume that cluster‐period sizes vary between clusters but not between periods within clusters, $K_{i0}=K_{i1}=K_{i-}$, Equation (16) simplifies to:

(18) $$\begin{array}{ccc} {\hat{\delta }}_{\mathrm{FE}} & = & \left[\frac{\left(\sum_{i=1}^{I}S_{i}\sum_{k=1}^{K_{i-}}Y_{i1k}\left(1\right)\right)-\left(\sum_{i=1}^{I}S_{i}\sum_{k=1}^{K_{i-}}Y_{i0k}\left(0\right)\right)}{\sum_{i=1}^{I}S_{i}K_{i-}}\right]\\ & & -\left[\frac{\left(\sum_{i=1}^{I}\left(1-S_{i}\right)\sum_{k=1}^{K_{i-}}Y_{i1k}\left(0\right)\right)-\left(\sum_{i=1}^{I}\left(1-S_{i}\right)\sum_{k=1}^{K_{i-}}Y_{i0k}\left(0\right)\right)}{\sum_{i=1}^{I}\left(1-S_{i}\right)K_{i-}}\right]. \end{array}$$

We can show that this estimator is consistent and asymptotically unbiased for the iATE when cluster‐period sizes do not vary within clusters:

(19) $${\hat{\delta }}_{\mathrm{FE}}\overset{P}{\to }E\left[\frac{1}{E\left[K_{i-}\right]}\sum_{k=1}^{K_{i-}}\left[Y_{i1k}\left(1\right)-Y_{i1k}\left(0\right)\right]\right].$$

Additional information is included in Supporting Information Appendix Section A.2.3.

### 4.4 Fixed‐Effects Model With Inverse Cluster‐Period Size Weighting (FEw) Estimator

Even when cluster‐period sizes vary between periods within clusters, $K_{i0}\neq K_{i1}$, we can still easily specify inverse cluster‐period size weights with the FEw estimator. Accordingly, this gives the following FEw treatment effect estimator:

(20) δ^FEw=∑i=1ISi1Ki1∑k=1Ki1Yi1k1−∑i=1ISi1Ki0∑k=1Ki0Yi0k0∑i=1ISi−∑i=1I1−Si1Ki1∑k=1Ki1Yi1k0−∑i=1I1−Si1Ki0∑k=1Ki0Yi0k0∑i=1I1−Si.

We can show that this estimator is consistent and asymptotically unbiased for the cATE:

(21) $${\hat{\delta }}_{\mathrm{FEw}}\overset{P}{\to }E\left[\frac{1}{K_{i1}}\sum_{k=1}^{K_{i1}}\left[Y_{i1k}\left(1\right)-Y_{i1k}\left(0\right)\right]\right].$$

Additionally, the FEw estimator is also unbiased for the cATE in expectation over the sampling distribution (Supporting Information Appendix Section A.2.4), similar to the IEEw estimator described in Section 4.2.

Importantly, the weights included in the FEw estimator deviate from those of Williamson et al. (2003) and are instead specified as cluster‐period‐specific weights. The inverse cluster‐period size weights can be easily defined in analyses with an independence correlation structure (IEEw and FEw) by specifying analyses with cluster‐period cell means.

Furthermore, as alluded to in Section 3, the FEw estimator is equivalent to a standard DiD estimator with the cluster‐period means. Without randomization, the estimator in expectation is still a theoretically consistent estimator for the cluster average treatment effect on the treated (cATT) estimand when assuming parallel trends. Additional information is included in Supporting Information Appendix Section A.2.4.

### 4.5 Mixed‐Effects Model Estimators

In the subsequent sections (Sections 4.6–4.9), we describe the different mixed‐effects models (weighted and unweighted), assuming $K_{i0}=K_{i1}=K_{i-}$ and cluster randomization. We will generally demonstrate that the mixed‐effects treatment effect estimators are of the form:

(22) $${\hat{\delta }}_{\mathrm{ME}}\overset{P}{\to }E\left[\frac{A_{i}/K_{i-}}{E\left[A_{i}\right]}\sum_{k=1}^{K_{i-}}\left[Y_{i1k}\left(1\right)-Y_{i1k}\left(0\right)\right]\right]$$

with mixed‐effects model‐specific values for $A_{i}$. Therefore, unless $A_{i}\propto 1$, ${\hat{\delta }}_{\mathrm{ME}}$ converges to a weighted average treatment effect estimand with data‐dependent model‐specific weights that may be challenging to interpret. This issue was discussed by Kahan et al. (2024) in the context of P‐CRTs without a baseline period and is generalized here in the context of PB‐CRTs. As a result, unweighted and weighted mixed‐effects models are not theoretically consistent estimators for the iATE nor cATE estimands, unless under some extreme conditions (Table 1).

### 4.6 Exchangeable Mixed‐Effects Model (EME) Estimator

The EME treatment effect estimator can be specified on the basis of Equation (9), where the diagonal terms $\left(D_{i}\right)$ and off‐diagonal terms $\left(F_{i}\right)$ in the block matrix corresponding to the observations within cluster i in the inverted correlation structure ${\tilde{V}}^{-1}$ are:

$$\;D_{i}=\frac{1}{\sigma_{w}^{2}}\left(\frac{\sigma_{w}^{2}+\left(2K_{i-}-1\right)\tau_{\alpha }^{2}}{\sigma_{w}^{2}+2K_{i-}\tau_{\alpha }^{2}}\right),$$

$$F_{i}=-\frac{1}{\sigma_{w}^{2}}\left(\frac{\tau_{\alpha }^{2}}{\sigma_{w}^{2}+2K_{i-}\tau_{\alpha }^{2}}\right),$$

assuming that cluster‐period sizes vary between clusters but not between periods within clusters, $K_{i0}=K_{i1}=K_{i-}$, with $2K_{i-}$ observations within each cluster.

Accordingly, using Equation (22), where:

$$\;A_{i}=K_{i-}\left[D_{i}+\left(K_{i-}-1\right)F_{i}\right]=\frac{1}{\sigma_{w}^{2}}\left(\frac{1+\left(K_{i-}-1\right)\rho }{1+\left(2K_{i-}-1\right)\rho }\right)K_{i-}$$

with intracluster correlation coefficient (ICC) $\rho =\frac{\tau_{\alpha }^{2}}{\tau_{\alpha }^{2}+\sigma_{w}^{2}}$, we can then show that the EME treatment effect estimator converges in probability to:

(23) $${\hat{\delta }}_{\mathrm{EME}}\overset{P}{\to }E\left[\frac{\left(\frac{1+\left(K_{i-}-1\right)\rho }{1+\left(2K_{i-}-1\right)\rho }\right)}{E\left[\left(\frac{1+\left(K_{i-}-1\right)\rho }{1+\left(2K_{i-}-1\right)\rho }\right)K_{i-}\right]}\sum_{k=1}^{K_{i-}}\left[Y_{i1k}\left(1\right)-Y_{i1k}\left(0\right)\right]\right].$$

This estimand includes a cluster‐specific weight that depends on both the cluster‐period size as well as the probability limit of the model‐based ICC estimator ρ. Therefore, in general, the ICC will dictate the magnitude of the targeted estimand, and therefore the analysis of a different outcome may inadvertently affect the definition of an estimand.

There are several special cases where this estimand reduces to a familiar and more interpretable estimand. First, when ρ=0 and there is simply no ICC, then $E\left[{\hat{\delta }}_{\mathrm{EME}}\right]\overset{P}{\to }E\left[\frac{1}{E[K_{i-}]}\sum_{k=1}^{K_{i-}}\left[Y_{i1k}\left(1\right)-Y_{i1k}\left(0\right)\right]\right]$, which is the iATE estimand. Second, when ρ=1 and the outcomes are perfectly correlated, ${\hat{\delta }}_{\mathrm{EME}}$ also converges to the iATE estimand. However, both conditions are unlikely to be realistic for PB‐CRTs. Additional information is included in Supporting Information Appendix Section A.2.5.

### 4.7 Exchangeable Mixed‐Effects Model With Inverse Cluster‐Period Size Weighting (EMEw) Estimator

The weighted EMEw treatment effect estimator can be specified similarly to the unweighted EME estimator, but with the diagonal terms $\left(D_{i}\right)$ and off‐diagonal terms $\left(F_{i}\right)$ in the block matrices of the inverted weighted correlation structure $W_{i}^{-1}$ (as described in Section 4) corresponding to the observations within cluster i specified as:

$$\;D_{i}=\frac{1}{\left(K_{i-}\right)\sigma_{w}^{2}}\left(\frac{\sigma_{w}^{2}+\left(2K_{i-}-1\right)\tau_{\alpha }^{2}}{\sigma_{w}^{2}+2K_{i-}\tau_{\alpha }^{2}}\right),$$

$$\;F_{i}=-\frac{1}{\left(K_{i-}\right)\sigma_{w}^{2}}\left(\frac{\tau_{\alpha }^{2}}{\sigma_{w}^{2}+2K_{i-}\tau_{\alpha }^{2}}\right),$$

assuming that cluster‐period sizes vary between clusters but not between periods within clusters, $K_{i0}=K_{i1}=K_{i-}$.

Accordingly, using Equation (22), where:

$$\;A_{i}=K_{i-}\left[D_{i}+\left(K_{i-}-1\right)F_{i}\right]=\frac{1}{\sigma_{w}^{2}}\left(\frac{1+\left(K_{i-}-1\right)\rho }{1+\left(2K_{i-}-1\right)\rho }\right),$$

with ICC $\rho =\frac{\tau_{\alpha }^{2}}{\tau_{\alpha }^{2}+\sigma_{w}^{2}}$, we can show that the EMEw treatment effect estimator converges in probability to:

(24) $$\begin{array}{c} {\hat{\delta }}_{\mathrm{EMEw}}\overset{P}{\to }E\left[\frac{\left(\frac{1+\left(K_{i-}-1\right)\rho }{1+\left(2K_{i-}-1\right)\rho }\right)}{E\left[\left(\frac{1+\left(K_{i-}-1\right)\rho }{1+\left(2K_{i-}-1\right)\rho }\right)\right]}\left(\frac{1}{K_{i-}}\sum_{k=1}^{K_{i-}}\left[Y_{i1k}\left(1\right)-Y_{i1k}\left(0\right)\right]\right)\right] \end{array}$$

Additional information is included in Supporting Information Appendix Section A.2.6.

Like the EME estimator (Equation 23), this estimand includes a cluster‐specific weight that depends on both the cluster‐period size as well as the probability limit of the model‐based ICC estimator ρ. Notably, for ρ→0 or 1, the $E[{\hat{\delta }}_{\mathrm{EMEw}}]\to \mathrm{cATE}$.

Surprisingly, unlike the results in (X. Wang et al. 2022) with a P‐CRT, we will further show in Section 5 that the expected value of the treatment effect with an exchangeable correlation structure and inverse cluster‐period size weights can often yield nearly unbiased estimates for the cATE in a PB‐CRT; hence, this approach is empirically robust for inferring the cATE estimand in a PB‐CRT.

### 4.8 Nested‐Exchangeable Mixed‐Effects Model (NEME) Estimator

The NEME treatment effect estimator can be specified on the basis of Equation (12). Assuming that cluster‐period sizes vary between clusters but not between periods within clusters, $K_{i0}=K_{i1}=K_{i-}$ with $2K_{i-}$ observations within each cluster, the specified correlation structure is then $\overset{ˇ}{V}=I_{I}\;⊗R_{i}^{\mathrm{NEME}}$, where:

$$\;R_{i}^{\mathrm{NEME}}=\left(\begin{array}{cc} R_{i1}^{\mathrm{NEME}} & R_{i2}^{\mathrm{NEME}}\\ R_{i3}^{\mathrm{NEME}} & R_{i4}^{\mathrm{NEME}} \end{array}\right),$$

with block matrices:

$$\begin{array}{ccc} R_{i1}^{\mathrm{NEME}} & = & R_{i4}^{\mathrm{NEME}}=\left(I_{K_{i-}}\sigma_{w}^{2}+J_{K_{i-}}\left(\tau_{\alpha }^{2}+\tau_{\gamma }^{2}\right)\right)\\ & = & \left(\begin{array}{cccc} \sigma_{w}^{2}+\tau_{\alpha }^{2}+\tau_{\gamma }^{2} & \tau_{\alpha }^{2}+\tau_{\gamma }^{2} & \text{…} & \tau_{\alpha }^{2}+\tau_{\gamma }^{2}\\ \tau_{\alpha }^{2}+\tau_{\gamma }^{2} & \sigma_{w}^{2}+\tau_{\alpha }^{2}+\tau_{\gamma }^{2} & \text{…} & \tau_{\alpha }^{2}+\tau_{\gamma }^{2}\\ \vdots & \vdots & \ddots & \vdots \\ \tau_{\alpha }^{2}+\tau_{\gamma }^{2} & \tau_{\alpha }^{2}+\tau_{\gamma }^{2} & \text{…} & \sigma_{w}^{2}+\tau_{\alpha }^{2}+\tau_{\gamma }^{2} \end{array}\right), \end{array}$$

$$\;R_{i2}^{\mathrm{NEME}}=R_{i3}^{\mathrm{NEME}}=\left(J_{K_{i-}}\tau_{\alpha }^{2}\right)=\left(\begin{array}{cccc} \tau_{\alpha }^{2} & \tau_{\alpha }^{2} & \text{…} & \tau_{\alpha }^{2}\\ \tau_{\alpha }^{2} & \tau_{\alpha }^{2} & \text{…} & \tau_{\alpha }^{2}\\ \vdots & \vdots & \ddots & \vdots \\ \tau_{\alpha }^{2} & \tau_{\alpha }^{2} & \text{…} & \tau_{\alpha }^{2} \end{array}\right),$$

where $I_{K_{i-}}$ is a $K_{i-}$ by $K_{i-}$ dimension identity matrix and $J_{K_{i-}}$ is a $K_{i-}$ by $K_{i-}$ dimension matrix of ones. Accordingly, ${\overset{ˇ}{V}}^{-1}=I_{I}\;⊗{\left(R_{i}^{\mathrm{NEME}}\right)}^{-1}$, where:

$${\left(R_{i}^{\mathrm{NEME}}\right)}^{-1\;}=\left(\begin{array}{cc} {\left[R_{i1}^{\mathrm{NEME}}-R_{i2}^{\mathrm{NEME}}{\left(R_{i1}^{\mathrm{NEME}}\right)}^{-1}R_{i2}^{\mathrm{NEME}}\right]}^{-1} & -{\left[R_{i1}^{\mathrm{NEME}}-R_{i2}^{\mathrm{NEME}}{\left(R_{i1}^{\mathrm{NEME}}\right)}^{-1}R_{i2}^{\mathrm{NEME}}\right]}^{-1}R_{i2}^{\mathrm{NEME}}{\left(R_{i1}^{\mathrm{NEME}}\right)}^{-1}\\ -{\left[R_{i1}^{\mathrm{NEME}}-R_{i2}^{\mathrm{NEME}}{\left(R_{i1}^{\mathrm{NEME}}\right)}^{-1}R_{i2}^{\mathrm{NEME}}\right]}^{-1}R_{i2}^{\mathrm{NEME}}{\left(R_{i1}^{\mathrm{NEME}}\right)}^{-1} & {\left[R_{i1}^{\mathrm{NEME}}-R_{i2}^{\mathrm{NEME}}{\left(R_{i1}^{\mathrm{NEME}}\right)}^{-1}R_{i2}^{\mathrm{NEME}}\right]}^{-1} \end{array}\right).$$

We define:

$${\left[R_{i1}^{\mathrm{NEME}}-R_{i2}^{\mathrm{NEME}}{\left(R_{i1}^{\mathrm{NEME}}\right)}^{-1}R_{i2}^{\mathrm{NEME}}\right]}^{-1}=\left(I_{K_{i-}}\left(D_{i}-F_{i}\right)+J_{K_{i-}}\left(F_{i}\right)\right)$$

where the diagonal terms $\left(D_{i}\right)$ and off‐diagonal terms $\left(F_{i}\right)$ are:

$$\;D_{i}=\frac{1}{\sigma_{w}^{2}}\left(\frac{\sigma_{w}^{2}+\left(K_{i-}-1\right)\left[\left(\tau_{\alpha }^{2}+\tau_{\gamma }^{2}\right)-\frac{\left(K_{i-}\right){\left(\tau_{\alpha }^{2}\right)}^{2}}{\sigma_{w}^{2}+\left(K_{i-}\right)\left(\tau_{\alpha }^{2}+\tau_{\gamma }^{2}\right)}\right]}{\sigma_{w}^{2}+\left(K_{i-}\right)\left[\left(\tau_{\alpha }^{2}+\tau_{\gamma }^{2}\right)-\frac{\left(K_{i-}\right){\left(\tau_{\alpha }^{2}\right)}^{2}}{\sigma_{w}^{2}+\left(K_{i-}\right)\left(\tau_{\alpha }^{2}+\tau_{\gamma }^{2}\right)}\right]}\right),$$

$$F_{i}=-\frac{1}{\sigma_{w}^{2}}\left(\frac{\left(\tau_{\alpha }^{2}+\tau_{\gamma }^{2}\right)-\frac{\left(K_{i-}\right){\left(\tau_{\alpha }^{2}\right)}^{2}}{\sigma_{w}^{2}+\left(K_{i-}\right)\left(\tau_{\alpha }^{2}+\tau_{\gamma }^{2}\right)}}{\sigma_{w}^{2}+\left(K_{i-}\right)\left[\left(\tau_{\alpha }^{2}+\tau_{\gamma }^{2}\right)-\frac{\left(K_{i-}\right){\left(\tau_{\alpha }^{2}\right)}^{2}}{\sigma_{w}^{2}+\left(K_{i-}\right)\left(\tau_{\alpha }^{2}+\tau_{\gamma }^{2}\right)}\right]}\right).$$

Accordingly, using Equation (22), where:

$$\begin{array}{ccc} \;A_{i} & = & K_{i-}\left[D_{i}+\left(K_{i-}-1\right)F_{i}\right]\\ & = & K_{i-}\left(\frac{1+\left(K_{i-}-1\right)\rho_{\mathrm{wp}}}{\left[{\left(1+\left(K_{i-}-1\right)\rho_{\mathrm{wp}}\right)}^{2}-{\left(K_{i-}\right)}^{2}\rho_{\mathrm{bp}}^{2}\right]\left(\tau_{\alpha }^{2}+\tau_{\gamma }^{2}+\sigma_{w}^{2}\right)}\right) \end{array}$$

with within‐period ICC $\left(\rho_{\mathrm{wp}}=\frac{\tau_{\alpha }^{2}+\tau_{\gamma }^{2}}{\tau_{\alpha }^{2}+\tau_{\gamma }^{2}+\sigma_{w}^{2}}\right)$ and between‐period ICC $\left(\rho_{\mathrm{bp}}=\frac{\tau_{\alpha }^{2}}{\tau_{\alpha }^{2}+\tau_{\gamma }^{2}+\sigma_{w}^{2}}\right)$, we can demonstrate that the NEME treatment effect estimator converges in probability to:

(25) $$\begin{array}{c} {\hat{\delta }}_{\mathrm{NEME}}\overset{P}{\to }\;E\left[\frac{\left(\frac{1+\left(K_{i-}-1\right)\rho_{wp}}{{\left(1+\left(K_{i-}-1\right)\rho_{wp}\right)}^{2}-{\left(K_{i-}\right)}^{2}\rho_{bp}^{2}}\right)}{E\left[\left(\frac{1+\left(K_{i-}-1\right)\rho_{wp}}{{\left(1+\left(K_{i-}-1\right)\rho_{wp}\right)}^{2}-{\left(K_{i-}\right)}^{2}\rho_{bp}^{2}}\right)K_{i-}\right]}\sum_{k=1}^{K_{i-}}\left[Y_{i1k}\left(1\right)-Y_{i1k}\left(0\right)\right]\right].\;\;\; \end{array}$$

Additional information is included in Supporting Information Appendix Section A.2.7.

The NEME estimand includes a cluster‐specific weight that depends on both the cluster‐period size and the probability limit of the within‐period and between‐period ICC estimators, $\rho_{wp}$ and $\rho_{bp}$, respectively. Notably, for $\rho_{wp}=\rho_{bp}\to 0$, the $E[{\hat{\delta }}_{\mathrm{NEME}}]\to \mathrm{iATE}$. However, this condition is given only for theoretical interest and is unlikely to be realistic for PB‐CRTs.

### 4.9 Nested‐Exchangeable Mixed‐Effects Model With Inverse Cluster‐Period Size Weighting (NEMEw) Estimator

The weighted NEMEw treatment effect estimator can be specified similarly to the unweighted NEME estimator but with the following terms:

$$\;D_{i}=\frac{1}{\left(K_{i-}\right)\sigma_{w}^{2}}\left(\frac{\sigma_{w}^{2}+\left(K_{i-}-1\right)\left[\left(\tau_{\alpha }^{2}+\tau_{\gamma }^{2}\right)-\frac{\left(K_{i-}\right){\left(\tau_{\alpha }^{2}\right)}^{2}}{\sigma_{w}^{2}+\left(K_{i-}\right)\left(\tau_{\alpha }^{2}+\tau_{\gamma }^{2}\right)}\right]}{\sigma_{w}^{2}+\left(K_{i-}\right)\left[\left(\tau_{\alpha }^{2}+\tau_{\gamma }^{2}\right)-\frac{\left(K_{i-}\right){\left(\tau_{\alpha }^{2}\right)}^{2}}{\sigma_{w}^{2}+\left(K_{i-}\right)\left(\tau_{\alpha }^{2}+\tau_{\gamma }^{2}\right)}\right]}\right),$$

$$\;F_{i}=-\frac{1}{\left(K_{i-}\right)\sigma_{w}^{2}}\left(\frac{\left(\tau_{\alpha }^{2}+\tau_{\gamma }^{2}\right)-\frac{\left(K_{i-}\right){\left(\tau_{\alpha }^{2}\right)}^{2}}{\sigma_{w}^{2}+\left(K_{i-}\right)\left(\tau_{\alpha }^{2}+\tau_{\gamma }^{2}\right)}}{\sigma_{w}^{2}+\left(K_{i-}\right)\left[\left(\tau_{\alpha }^{2}+\tau_{\gamma }^{2}\right)-\frac{\left(K_{i-}\right){\left(\tau_{\alpha }^{2}\right)}^{2}}{\sigma_{w}^{2}+\left(K_{i-}\right)\left(\tau_{\alpha }^{2}+\tau_{\gamma }^{2}\right)}\right]}\right).$$

Accordingly, using Equation (22), where:

$$\begin{array}{ccc} \;A_{i} & = & K_{i-}\left[D_{i}+\left(K_{i-}-1\right)F_{i}\right]\\ & = & \left(\frac{1+\left(K_{i-}-1\right)\rho_{wp}}{\left[{\left(1+\left(K_{i-}-1\right)\rho_{wp}\right)}^{2}-{\left(K_{i-}\right)}^{2}\rho_{bp}^{2}\right]\left(\tau_{\alpha }^{2}+\tau_{\gamma }^{2}+\sigma_{w}^{2}\right)}\right) \end{array}$$

with within‐period ICC $\left(\rho_{\mathrm{wp}}=\frac{\tau_{\alpha }^{2}+\tau_{\gamma }^{2}}{\tau_{\alpha }^{2}+\tau_{\gamma }^{2}+\sigma_{w}^{2}}\right)$ and between‐period ICC $\left(\rho_{\mathrm{bp}}=\frac{\tau_{\alpha }^{2}}{\tau_{\alpha }^{2}+\tau_{\gamma }^{2}+\sigma_{w}^{2}}\right)$, we can show that the NEMEw treatment effect estimator converges in probability to:

(26) $$\begin{array}{c} {\hat{\delta }}_{\mathrm{NEMEw}}\overset{P}{\to }E\left[\frac{\left(\frac{1+\left(K_{i-}-1\right)\rho_{wp}}{{\left(1+\left(K_{i-}-1\right)\rho_{wp}\right)}^{2}-{\left(K_{i-}\right)}^{2}\rho_{bp}^{2}}\right)}{E\left[\frac{1+\left(K_{i-}-1\right)\rho_{wp}}{{\left(1+\left(K_{i-}-1\right)\rho_{wp}\right)}^{2}-{\left(K_{i-}\right)}^{2}\rho_{bp}^{2}}\right]}\left(\frac{1}{K_{i-}}\sum_{k=1}^{K_{i-}}\left[Y_{i1k}\left(1\right)-Y_{i1k}\left(0\right)\right]\right)\right]\;\;\; \end{array}$$

Additional information is included in Supporting Information Appendix Section A.2.8.

The NEMEw estimand includes a cluster‐specific weight that depends on both the cluster‐period size as well as the probability limit of the within‐period and between‐period ICC estimators, $\rho_{wp}$ and $\rho_{bp}$, respectively. Notably, for $\rho_{wp}=\rho_{bp}\to 0$, the $E[{\hat{\delta }}_{\mathrm{NEMEw}}]\to \mathrm{cATE}$. However, as mentioned previously, this condition is only given for theoretical interest and is unlikely to be realistic for PB‐CRTs.

## 5 A Further Evaluation of EMEw and NEMEw Estimators

As we previously demonstrated in Sections 4.5–4.9, the unweighted and weighted mixed‐effects models are not theoretically consistent estimators for the iATE nor the cATE and instead converge to weighted versions of these estimands.

In this section, we will further evaluate the EMEw and NEMEw treatment effect estimators, characterize their estimand weights, and illustrate the pattern of the estimators’ biases for the cATE estimand. We focus on the estimand weights in the EMEw and NEMEw estimators for the cATE estimand and not in the EME and NEME estimators for the iATE estimand due to the simpler interpretation.

In Equation (22), we demonstrated that the exchangeable and nested‐exchangeable mixed effects model estimators share a common general form in the analysis of PB‐CRTs. Notably, the EMEw treatment effect estimator converges in probability to a weighted average of the cluster‐specific cATE estimands:

$${\hat{\delta }}_{\mathrm{EMEw}}\overset{P}{\to }E\left[\lambda_{\mathrm{EMEw}}\left(\frac{1}{K_{i-}}\sum_{k=1}^{K_{i-}}\left[Y_{i1k}\left(1\right)-Y_{i1k}\left(0\right)\right]\right)\right]$$

with cluster‐specific estimand weights:

$$\;\lambda_{\mathrm{EMEw}}=\frac{\left(\frac{1+\left(K_{i-}-1\right)\rho }{1+\left(2K_{i-}-1\right)\rho }\right)}{E\left[\left(\frac{1+\left(K_{i-}-1\right)\rho }{1+\left(2K_{i-}-1\right)\rho }\right)\right]}.$$

Similarly, the NEMEw treatment effect estimator converges in probability to a weighted average of the cluster‐specific cATE estimands:

$${\hat{\delta }}_{\mathrm{NEMEw}}\overset{P}{\to }E\left[\lambda_{\mathrm{NEMEw}}\left(\frac{1}{K_{i-}}\sum_{k=1}^{K_{i-}}\left[Y_{i1k}\left(1\right)-Y_{i1k}\left(0\right)\right]\right)\right]$$

with cluster‐specific estimand weights:

$$\;\lambda_{\mathrm{NEMEw}}=\frac{\left(\frac{1+\left(K_{i-}-1\right)\rho_{wp}}{{\left(1+\left(K_{i-}-1\right)\rho_{wp}\right)}^{2}-{\left(K_{i-}\right)}^{2}\rho_{bp}^{2}}\right)}{E\left[\frac{1+\left(K_{i-}-1\right)\rho_{wp}}{{\left(1+\left(K_{i-}-1\right)\rho_{wp}\right)}^{2}-{\left(K_{i-}\right)}^{2}\rho_{bp}^{2}}\right]}.$$

When the estimand weights $\lambda_{\mathrm{EMEw}}=1$ and $\lambda_{\mathrm{NEMEw}}=1$, the EMEw and NEMEw treatment effect estimators converge to the cATE estimand. We plot some numerical examples to explore when these estimand weights deviate from 1 and provide numerical insights into the pattern of the bias in the EMEw and NEMEw estimators for the cATE estimand in the presence of informative cluster sizes.

**FIGURE 2:**
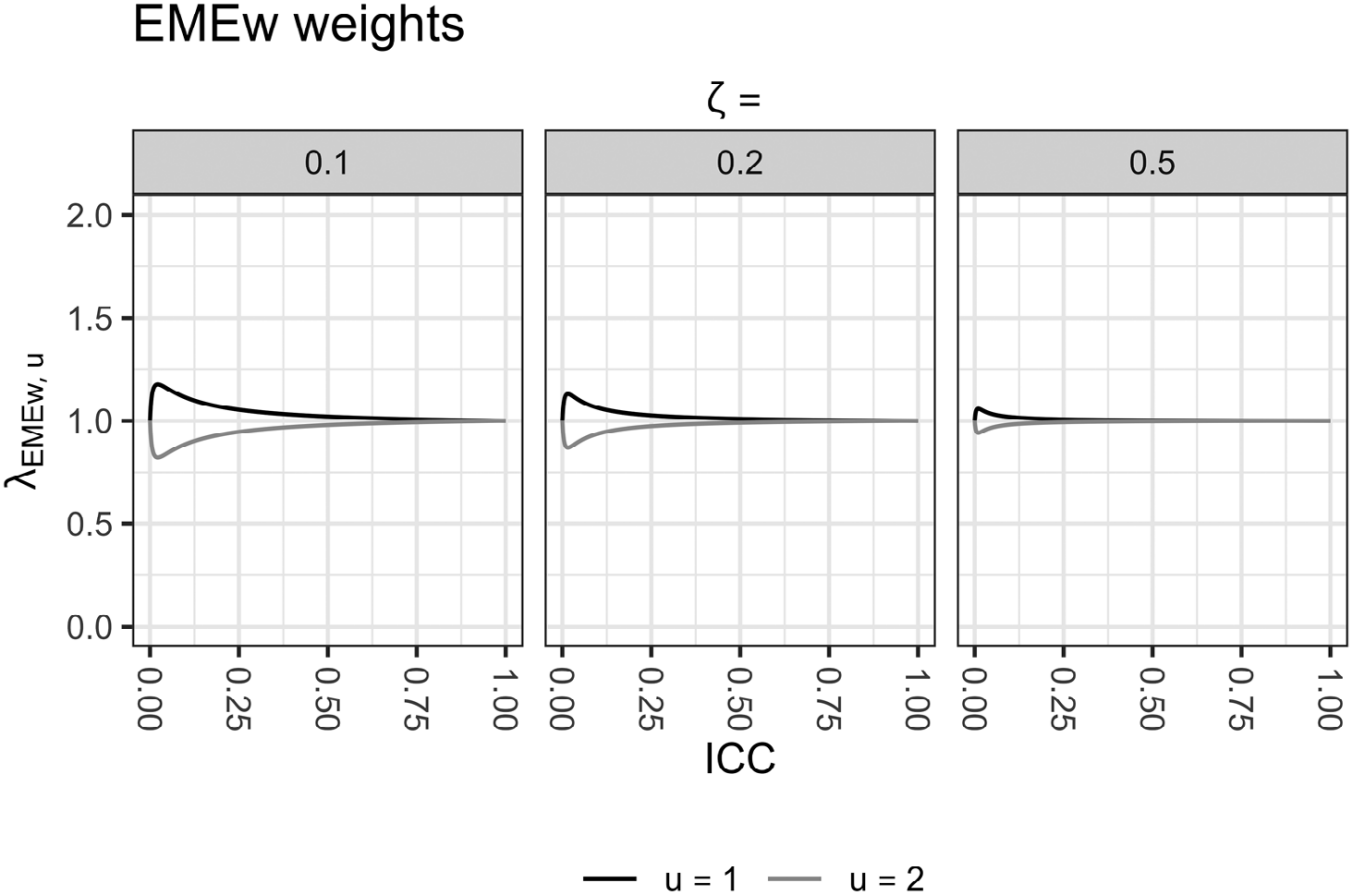
EMEw estimand weights $\lambda_{\mathrm{EMEw},u}$ are plotted across two subpopulations u=1,2 and different values of ICC, with subpopulation‐specific cluster‐period sizes $K_{i-,1}$ and $K_{i-,2}$ that differ by a ratio of $\zeta =\frac{K_{i-,1}}{K_{i-,2}}$, and have equal probabilities of being sampled P(u=1)=P(u=2)=0.5. EMEw, exchangeable mixed‐effects model with inverse cluster‐period size weighting; ICC, intracluster correlation coefficient.

In Figure 2, we plot the values of the estimand weights from the EMEw estimator $\lambda_{\mathrm{EMEw},u}$ across different ICC values ρ∈[0,1], in scenarios with two subpopulations u=1,2 with fixed subpopulation‐specific cluster‐period sizes $K_{i-,1}$ and $K_{i-,2}$ that differ by a ratio of $\zeta =\frac{K_{i-,1}}{K_{i-,2}}$, and have equal probabilities of being sampled P(u=1)=0.5 and P(u=2)=1−P(u=1).

**FIGURE 3:**
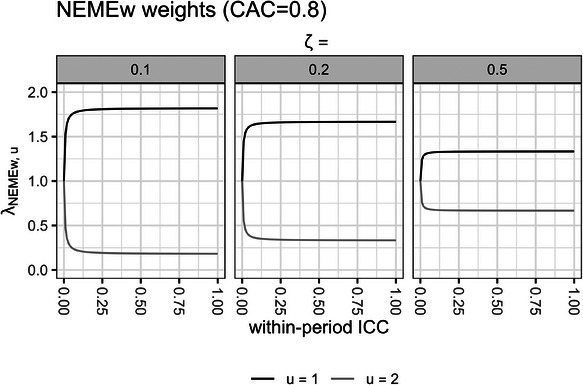
NEMEw estimand weights $\lambda_{\mathrm{NEMEw},u}$ are plotted across two subpopulations u=1,2 and different values of within‐period ICC and fixed cluster auto‐correlation CAC=0.8, with subpopulation‐specific cluster‐period sizes $K_{i-,1}$ and $K_{i-,2}$ that differ by a ratio of $\zeta =\frac{K_{i-,1}}{K_{i-,2}}$, and have equal probabilities of being sampled P(u=1)=P(u=2)=0.5. NEMEw, nested‐exchangeable mixed‐effects model with inverse cluster‐period size weighting; ICC, intracluster correlation coefficient.

In Figure 3, we plot the values of the estimand weights from the NEMEw estimator $\lambda_{\mathrm{NEMEw},u}$ across different within‐period ICC values $\rho_{wp}\in \left[0,1\right]$ with cluster auto‐correlation $\mathrm{CAC}=\frac{\rho_{bp}}{\rho_{wp}}=0.8$, in scenarios with two subpopulations u=1,2 with fixed subpopulation‐specific cluster‐period sizes $K_{i-,1}$ and $K_{i-,2}$ that differ by a ratio of $\zeta =\frac{K_{i-,1}}{K_{i-,2}}$. Again, these subpopulations have equal probabilities of being sampled from the overall population P(u=1)=0.5 and P(u=2)=1−P(u=1).

Overall, we observe that the estimand weights $\lambda_{\mathrm{EMEw}}$ in the EMEw treatment effect estimator are slightly elevated in scenarios with low ICC, low cluster‐period size ratios ζ, and high sampling probabilities P(u=1) (Figure 2). Accordingly, the EMEw treatment effect estimates will be more biased for the cATE estimand in these scenarios. However, the weights are minimized for larger values of ICC (Figure 2), which would result in a less biased EMEw treatment effect estimator.

Notably, the maximum amount of difference between the estimand weights of the two subpopulations in the EMEw estimator $\left(\lambda_{\mathrm{EMEw},1}-\lambda_{\mathrm{EMEw},2}\right)$ (Figure 2) is still considerably lower than that of the NEMEw estimator $\left(\lambda_{\mathrm{NEMEw},1}-\lambda_{\mathrm{NEMEw},\;2}\right)$ (Figure 3). We observe that the estimand weights $\lambda_{\mathrm{NEMEw}}$ in the NEMEw treatment effect estimator are often severely unbalanced and remain so for nearly all values of within‐period ICC (Figure 3). This indicates that the NEMEw treatment effect estimator will generally yield biased estimates for the cATE estimand across most scenarios.

We observe that the maximum difference between the EMEw estimand weights increases with larger values of ζ. However, graphing up to an extreme value of ζ=0.001 with the optimal P(u=1) and ρ to maximize the bias still yielded relatively balanced estimand weights with fairly small values for $\lambda_{\mathrm{EMEw},1}-\lambda_{\mathrm{EMEw},2}$ (Supporting Information Appendix Section A.3.3).

In the EMEw estimator, given two subpopulations each with fixed subpopulation‐specific cluster‐period sizes $K_{i-,1}$ and $K_{i-,2}$, we can prove that the maximum difference between the estimand weights and accordingly the maximum amount of bias occur when the ICC is equivalent to

$$\rho =\frac{1}{1+\sqrt{2K_{i-,1}K_{i-,2}}}$$

as demonstrated in Supporting Information Appendix Section A.3.1. Subsequently, we also prove that the maximum amount of bias occurs when the probability of belonging to subpopulation u=1 is equivalent to:

$$P\left(u=1\right)=\frac{\sqrt{\lambda_{\mathrm{EMEw},2}}}{\sqrt{\lambda_{\mathrm{EMEw},1}}+\sqrt{\lambda_{\mathrm{EMEw},2}}}$$

which tends to be ≈0.5, as demonstrated in Supporting Information Appendix Section A.3.2.

Overall, we demonstrate that in the analysis of a PB‐CRT, the EMEw treatment effect estimator is more resistant to bias than the NEMEw treatment effect estimator in estimating the cATE estimand. This numerical exploration in a simplified example provides useful insights into the simulation results in Section 6.

## 6 Simulations

We simulated PB‐CRT data with continuous outcomes and homogeneous or heterogeneous treatment effects that vary according to cluster size to empirically demonstrate the theoretical results derived in Section 4.

Potential outcomes were generated with the following data‐generating process (DGP):

$$Y_{i0k}\left(0\right)=\mu +\alpha_{i}+\gamma_{ij}+e_{ijk}$$

$$Y_{i1k}\left(0\right)=\mu +\Phi_{1}+\alpha_{i}+\gamma_{ij}+e_{ijk}$$

$$Y_{i1k}\;\left(1\right)=\mu +\delta_{u}+\Phi_{1}+\alpha_{i}+\gamma_{ij}+e_{ijk}$$

$$\alpha_{i}\overset{\mathrm{iid}}{∼}N\left(0,\tau_{\alpha }^{2}\right)$$

$$\gamma_{\mathrm{ij}}\overset{\mathrm{iid}}{∼}N\left(0,\tau_{\gamma }^{2}\right)$$

$$e_{\mathrm{ijk}}\overset{\mathrm{iid}}{∼}N\left(0,\sigma_{w}^{2}=1\right)$$

with $\delta_{u}$ being the u subpopulation‐specific heterogeneous treatment effect. We set μ=1 with a period effect $\Phi_{1}=0.2$. Cluster random intercepts and cluster‐period random interaction terms are drawn from independent normal distributions with variances $\tau_{\alpha }^{2}=0.053$ and $\tau_{\gamma }^{2}=0.013$, respectively, to yield a within‐period ICC of $\rho_{\mathrm{wp}}=\frac{\tau_{\alpha }^{2}+\tau_{\gamma }^{2}}{\tau_{\alpha }^{2}+\tau_{\gamma }^{2}+\sigma_{w}^{2}}\approx 0.06$, between‐period ICC of $\rho_{\mathrm{bp}}=\frac{\tau_{\alpha }^{2}}{\tau_{\alpha }^{2}+\tau_{\gamma }^{2}+\sigma_{w}^{2}}\approx 0.05$, and a cluster auto‐correlation of $\mathrm{CAC}=\frac{\rho_{bp}}{\rho_{wp}}=\frac{\tau_{\alpha }^{2}}{\tau_{\alpha }^{2}+\tau_{\gamma }^{2}}=0.8$.

For illustration, we simulated data from a 10 cluster, 2 period PB‐CRT. Half of the clusters arose from subpopulation u=1 with the other half from population u=2 (accordingly, the cluster sampling probabilities are P(u=1)=P(u=2)=0.5). Cluster‐period cell sizes were fixed between periods within clusters for subpopulations u=1 and 2, and were generated with $K_{i-,1}∼\mathrm{Poisson}\left(20\right)$ and $K_{i-,2}∼\mathrm{Poisson}\left(100\right)$, respectively. Half of the clusters were randomized to receive the treatment in period j=1, with the other half receiving the control throughout the trial.

In scenarios with noninformative cluster sizes, we simulated a homogeneous treatment effect $\delta_{1}=\delta_{2}=0.35$. In scenarios with informative cluster sizes between subpopulations u=1 and 2, we simulated heterogeneous treatment effects $\delta_{1}=0.2$ and $\delta_{2}=0.5$. Given the cluster‐level treatment indicator $X_{ij}$, the observed outcomes during the follow‐up period j=1 are $Y_{i1k}=X_{i1}Y_{i1k}\left(1\right)+\left(1-X_{i1}\right)Y_{i1k}\left(0\right)$.

We analyzed the data with the IEE, FE, EME, NEME, and IEEw, FEw, EMEw, NEMEw models. With the described DGP, the true iATE estimand is:

$$\begin{array}{ccc} \mathrm{iATE} & = & E\left[\frac{1}{E\left[K_{i1}\right]}\sum_{k=1}^{K_{i1}}\left[Y_{i1k}\left(1\right)-Y_{i1k}\left(0\right)\right]\right]=\frac{E\left[E\left[K_{i-}\delta_{u}|u\right]\right]}{E\left[E\left[K_{i-}|u\right]\right]}\\ & = & \frac{P\left(u=1\right)E\left[K_{i-}\delta_{u}|u=1\right]+P\left(u=2\right)E\left[K_{i-}\delta_{u}|u=2\right]}{P\left(u=1\right)E\left[K_{i-}|u=1\right]+P\left(u=2\right)E\left[K_{i-}|u=2\right]}\\ & = & \frac{0.5\left(20\delta_{1}\right)+0.5\left(100\delta_{2}\right)}{0.5\left(20\right)+0.5\left(100\right)}=\frac{10\delta_{1}+50\delta_{2}}{60} \end{array}$$

which is equal to 0.35 in scenarios with non‐informative cluster sizes ($\delta_{1}=\delta_{2}=0.35$) and 0.45 in scenarios with informative cluster sizes ($\delta_{1}=0.2$ and $\delta_{2}=0.5$). The true cATE estimand is then:

$$\begin{array}{ccc} \mathrm{cATE} & = & E\left[\frac{1}{K_{i1}}\sum_{k=1}^{K_{i1}}\left[Y_{i1k}\left(1\right)-Y_{i1k}\left(0\right)\right]\right]=E\left[E\left[\delta_{u}|u\right]\right]\\ & = & P\left(u=1\right)E\left[\delta_{u}|u=1\right]+P\left(u=2\right)E\left[\delta_{u}|u=2\right]\\ & = & 0.5\left(\delta_{1}\right)+0.5\left(\delta_{2}\right)=0.35 \end{array}$$

in simulation scenarios with non‐informative cluster sizes or informative cluster sizes.

For each scenario, we simulated 1000 PB‐CRT datasets. Accordingly, we presented the results for the iATE and cATE in terms of percent relative bias and root mean square error. Furthermore, we studied the accuracy of different variance estimators and compared the model‐based and the leave‐one‐cluster‐out jackknife variance estimators alongside the true Monte Carlo variance (the variance of the 1000 simulated point estimates) and used the model‐based and jackknife variance estimators to calculate the coverage probability (CP) of the 95% Wald confidence intervals and the power.

The weighted mixed‐effects models were run using the *WeMix* package in R (Bailey et al. 2018). Notably, the *WeMix* package (Bailey et al. 2018) by default returns the sandwich variance estimator described by Rabe‐Hesketh and Skrondal (2006). For demonstration purposes, we manually programmed the model‐based variance estimators for the EMEw and NEMEw estimators using the output from *WeMix*, although inference with this model‐based variance estimator is not recommended for weighted regression methods. The leave‐one‐cluster‐out jackknife variance estimator was manually programmed in R version 4.3.2 following the description by Bell and McCaffrey (2002). A similar jackknife variance estimator has been previously demonstrated to yield robust inference with mixed‐effects model misspecification in SW‐CRTs (Ouyang et al. 2024). We additionally evaluated the sandwich variance estimator described by Liang and Zeger (1986) and the bias‐reduced linearization method recommended for inference with two‐way FE models (J. E. Pustejovsky and Tipton 2018). The bias‐reduced linearization method and other cluster‐robust variance estimators can be implemented with the *clubSandwich* package (J. Pustejovsky 2016) in R for the IEE, IEEw, FE, and FEw models. However, *clubSandwich* is not compatible with *WeMix*. Therefore, the bias‐reduced linearization method is not easily implemented with the weighted mixed‐effects models. The results from the bias‐reduced linearization (J. E. Pustejovsky and Tipton 2018) variance estimator, the Liang and Zeger (1986) sandwich variance estimator, and the Rabe‐Hesketh and Skrondal (2006) sandwich variance estimator for the different treatment effect estimators are reported in Supporting Information Web Appendix Section A.4.1.

### 6.1 Simulation Results With Non‐Informative Cluster Sizes

**FIGURE 4:**
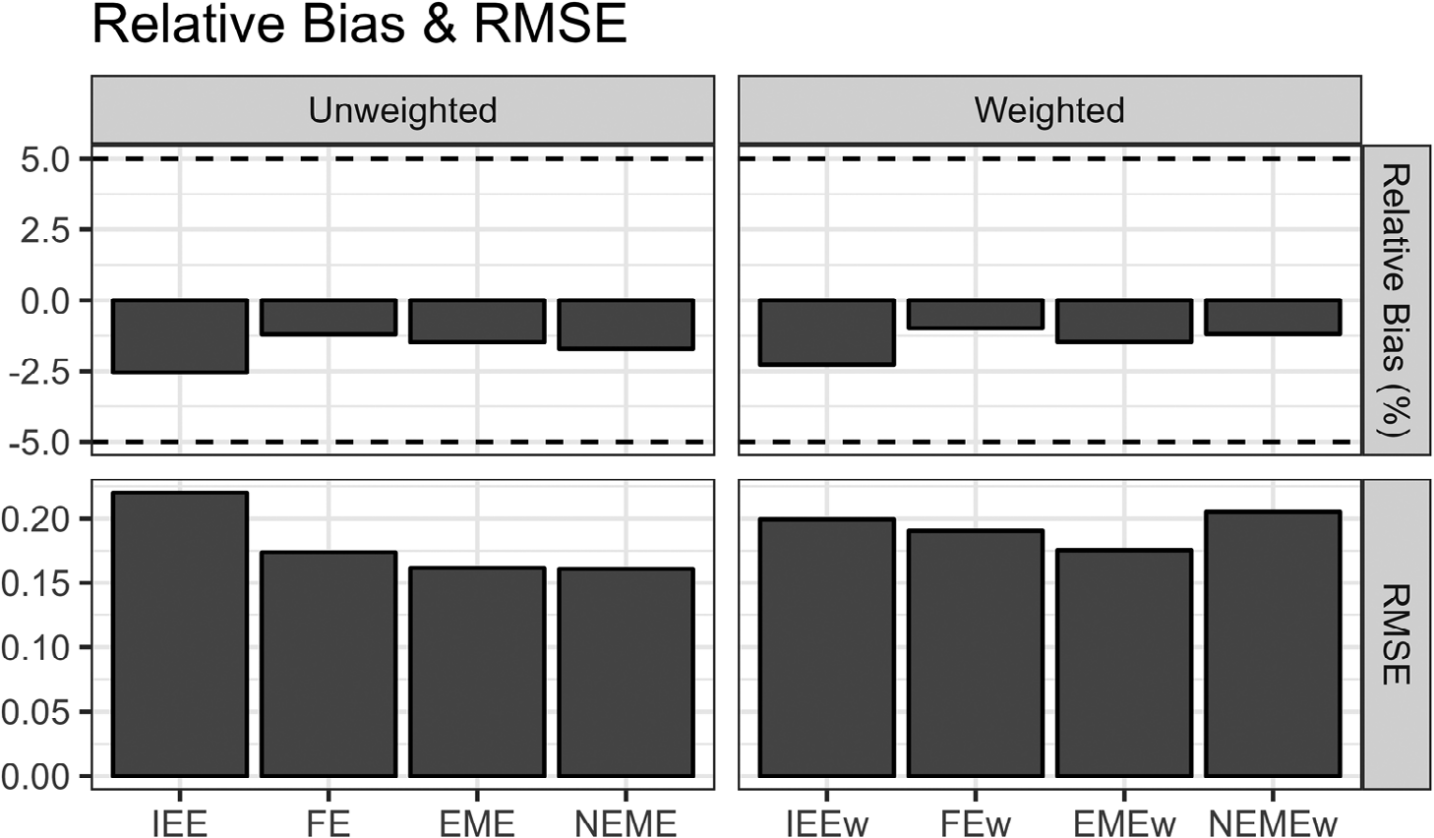
Simulation bias and RMSE results in scenarios with homogeneous treatment effects (non‐informative cluster sizes). Dashed lines show a relative bias (%) of 5% and −5%. EME, exchangeable mixed‐effects; EMEw, exchangeable mixed‐effects model with inverse cluster‐period size weighting; FE, fixed‐effects; FEw, fixed‐effects model with inverse cluster‐period size weighting; IEE, independence estimating equation; IEEw, independence estimating equation with inverse cluster‐period size weighting; NEME, nested‐exchangeable mixed‐effects; NEMEw, nested‐exchangeable mixed‐effects model with inverse cluster‐period size weighting.

The simulation results in scenarios with homogeneous treatment effects (non‐informative cluster sizes) are shown below. With such homogeneous treatment effects, the iATE and cATE estimands coincide under our DGP. As expected, all the unweighted and weighted treatment effect estimators were unbiased for the true average treatment effect estimand (Figure 4). Comparing the weighted models against their unweighted counterparts, we observe that the IEEw model can yield lower RMSE than the IEE model, whereas the NEMEw model can yield a considerably higher RMSE than the NEME model (Figure 4).

**FIGURE 5:**
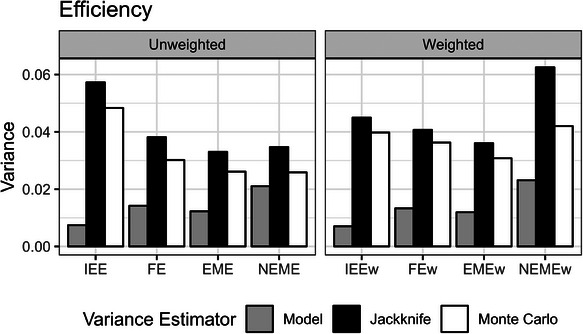
The average of the model‐based and leave‐one‐cluster jackknife variance estimates of the different unweighted and weighted models, over the 1000 simulation replicates, graphed alongside the corresponding true Monte Carlo variances. EME, exchangeable mixed‐effects; EMEw, exchangeable mixed‐effects model with inverse cluster‐period size weighting; FE, fixed‐effects; FEw, fixed‐effects model with inverse cluster‐period size weighting; IEE, independence estimating equation; IEEw, independence estimating equation with inverse cluster‐period size weighting; NEME, nested‐exchangeable mixed‐effects; NEMEw, nested‐exchangeable mixed‐effects model with inverse cluster‐period size weighting.

The averages of the model‐based and leave‐one‐cluster‐out jackknife variance estimates are plotted in Figure 5, alongside the Monte Carlo variance. The Monte Carlo variance has also been commonly referred to as the “empirical,” “observed,” or “sampling” variances of the point estimates over the simulation replicates (Morris et al. 2019). The model‐based and jackknife variance estimators explicitly target the Monte Carlo variance, with systematic deviations representing a bias in the estimation of the variance (Morris et al. 2019). Comparing the variance estimates in Figure 5, where the true underlying DGP has a nested‐exchangeable correlation structure, we observe that the jackknife variance estimates roughly approximate the Monte Carlo variances, even in scenarios where the correlation structure is misspecified.

Across the unweighted analyses in our simulations, the IEE model had the largest jackknife variance estimates and Monte Carlo variances. In contrast, the FE, EME, and NEME models notably all had smaller and similar jackknife variance estimates and Monte Carlo variances. On the basis of these empirical observations of the Monte Carlo variances, the IEE treatment effect estimator is the least efficient of the described estimators. This can occur because the IEE estimator only makes “vertical” comparisons within periods (as described in Section 3), whereas the other described estimators use information both within and between periods and tend to be more efficient.

Across the weighted analyses in our simulations, the NEMEw model has much larger model‐based and jackknife variance estimates than the corresponding estimates in the other weighted analyses. Furthermore, the NEMEw model had the largest Monte Carlo variances that were poorly approximated by the jackknife variance estimators. In contrast, the IEEw, FEw, and EMEw models had lower Monte Carlo variances, which were more closely approximated by the jackknife variance estimators. Overall, the FEw and EMEw models had the lowest Monte Carlo and jackknife variances across the weighted analyses.

We compared the weighted models against their unweighted counterparts and observed that modeling with inverse cluster‐period size weights may or may not lead to worse efficiency (Figure 5). The NEMEw model had very inflated model‐based, jackknife, and Monte Carlo variances in comparison to the unweighted NEME model (Figure 5). Conversely, the IEEw model yielded comparatively lower jackknife and Monte Carlo variances than the unweighted IEE model. In contrast, including inverse cluster‐period size weights in the FEw and EMEw models had little effect on the jackknife variance estimates and Monte Carlo variances compared to the corresponding unweighted models (FE and EME models) (Figure 5).

**FIGURE 6:**
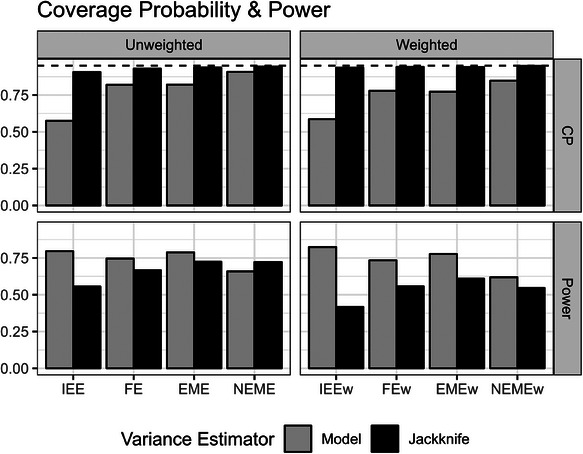
The coverage probability of the 95% confidence interval and the power of the different unweighted and weighted models using the model‐based and leave‐one‐cluster jackknife variance estimators in scenarios with a homogeneous treatment effect (non‐informative cluster sizes). Dashed lines show a coverage probability of 0.95. EME, exchangeable mixed‐effects; EMEw, exchangeable mixed‐effects model with inverse cluster‐period size weighting; FE, fixed‐effects; FEw, fixed‐effects model with inverse cluster‐period size weighting; IEE, independence estimating equation; IEEw, independence estimating equation with inverse cluster‐period size weighting; NEME, nested‐exchangeable mixed‐effects; NEMEw, nested‐exchangeable mixed‐effects model with inverse cluster‐period size weighting.

With the true underlying DGP having a nested‐exchangeable correlation structure, all analyses, regardless of their specified correlation structure, had proper coverage of the 95% confidence intervals with the jackknife variance estimator (Figure 6). This corresponds with previous work that demonstrated the jackknife variance estimator can yield robust inference with correlation structure misspecification in SW‐CRTs (Ouyang et al. 2024). Subsequently, the power across the different analyses is only slightly reduced when using the jackknife variance estimator compared to the power using the model‐based variance estimator in the correctly specified NEME and NEMEw models. In fact, the FE and EME models with jackknife variance estimators can have greater or comparable power to the correctly specified NEME estimators with model‐based variance estimators, as is also the case for their weighted counterparts (Figure 6).

Overall, we recommend using the leave‐one‐cluster‐out jackknife variance estimator for inference due to its robustness despite misspecification of the true correlation structure. We empirically observe that the jackknife variance estimates roughly approximate the Monte Carlo variances. Furthermore, it's simple to manually program, which makes it easily compatible with different R packages, including *WeMix*. In contrast, the sandwich (Liang and Zeger 1986; Rabe‐Hesketh and Skrondal 2006) and bias‐reduced linearization (J. E. Pustejovsky and Tipton 2018) variance estimators routinely underestimated the Monte Carlo variances and yielded under‐coverage of the 95% confidence intervals across the different treatment effect estimators (Supporting Information Web Appendix Section A.4.1).

### 6.2 Simulation Results With Informative Cluster Sizes

**FIGURE 7:**
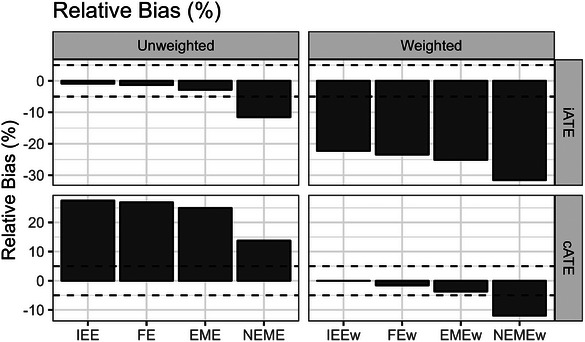
Simulation bias results for the iATE and cATE estimands in scenarios with heterogeneous treatment effects (informative cluster sizes). Dashed lines show a relative bias (%) of 5% and −5%. EME, exchangeable mixed‐effects; EMEw, exchangeable mixed‐effects model with inverse cluster‐period size weighting; FE, fixed‐effects; FEw, fixed‐effects model with inverse cluster‐period size weighting; IEE, independence estimating equation; IEEw, independence estimating equation with inverse cluster‐period size weighting; NEME, nested‐exchangeable mixed‐effects; NEMEw, nested‐exchangeable mixed‐effects model with inverse cluster‐period size weighting.

The simulation results in scenarios with heterogeneous outcomes (informative cluster sizes) are shown below. As expected, the IEE and FE treatment effect estimators were unbiased for the true iATE estimand, and the IEEw and FEw treatment effect estimators were unbiased for the true cATE estimand (Figure 7). Surprisingly, the EME and EMEw treatment effect estimators were also empirically unbiased across our simulation replicates for the iATE and cATE, respectively (Figure 7). The possible mechanics for this phenomenon were discussed earlier in Section 5, and this result clearly differs from that presented by Wang et al. (2022) for a corresponding estimator in P‐CRTs (X. Wang et al. 2022). In contrast, the NEME and NEMEw treatment effect estimators were biased (accepting over 10% relative bias) for the iATE and cATE, respectively, in PB‐CRT scenarios with informative cluster sizes (Figure 7).

**FIGURE 8:**
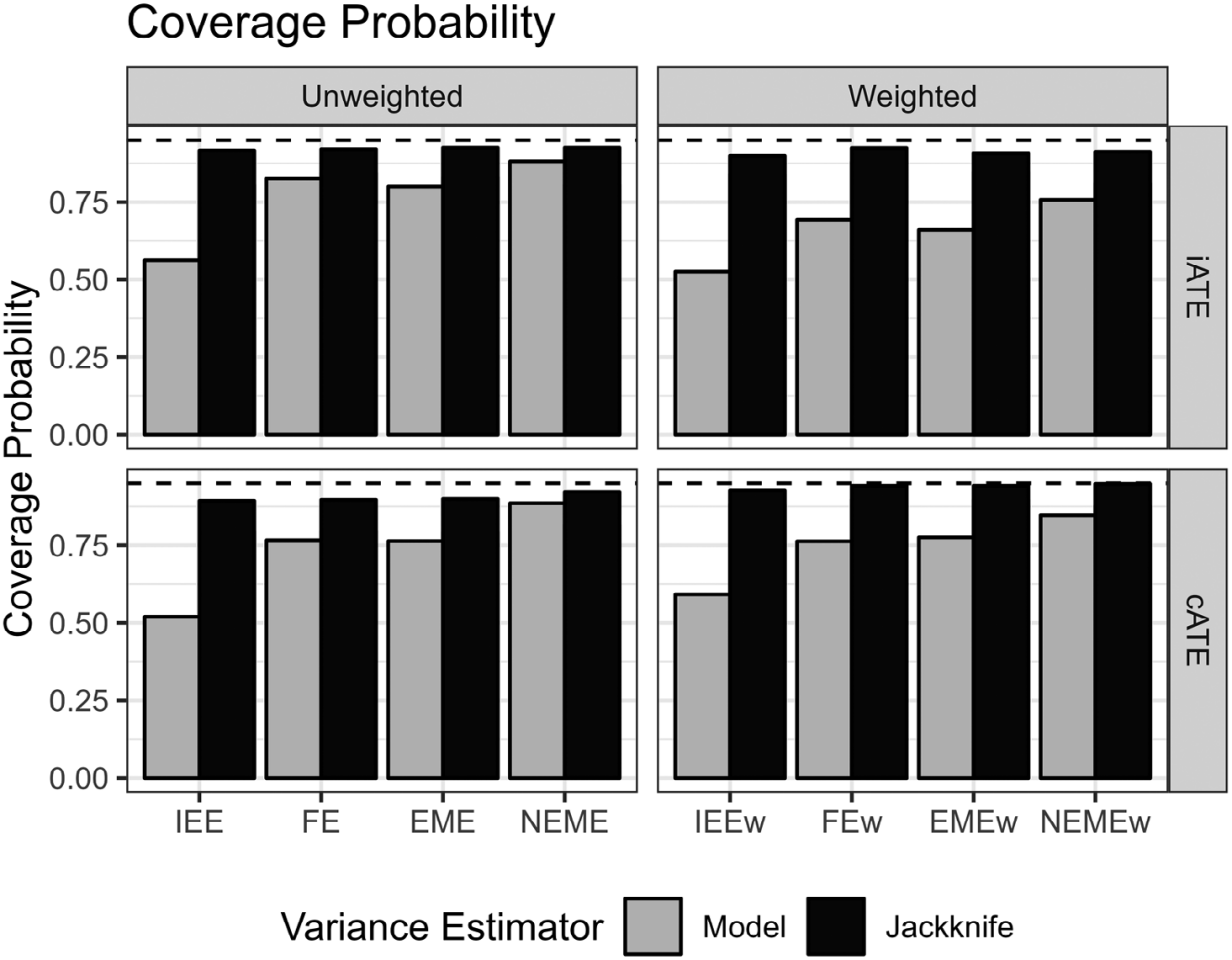
The coverage probability of the 95% confidence interval of the different unweighted and weighted models using the model‐based and leave‐one‐cluster jackknife variance estimators in scenarios with a heterogeneous treatment effect (informative cluster sizes). Dashed lines show a coverage probability of 0.95. EME, exchangeable mixed‐effects; EMEw, exchangeable mixed‐effects model with inverse cluster‐period size weighting; FE, fixed‐effects; FEw, fixed‐effects model with inverse cluster‐period size weighting; IEE, independence estimating equation; IEEw, independence estimating equation with inverse cluster‐period size weighting; NEME, nested‐exchangeable mixed‐effects; NEMEw, nested‐exchangeable mixed‐effects model with inverse cluster‐period size weighting.

**FIGURE 9:**
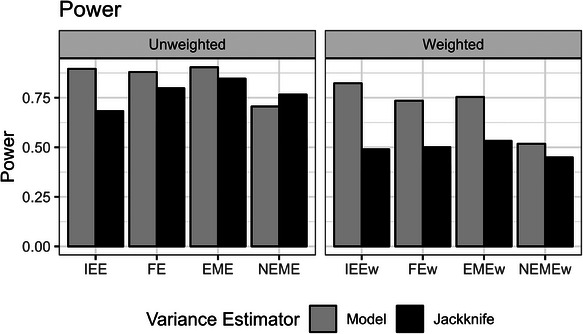
The power of the different unweighted and weighted models using the model‐based and leave‐one‐cluster jackknife variance estimators in scenarios with a heterogeneous treatment effect (informative cluster sizes). EME, exchangeable mixed‐effects; EMEw, exchangeable mixed‐effects model with inverse cluster‐period size weighting; FE, fixed‐effects; FEw, fixed‐effects model with inverse cluster‐period size weighting; IEE, independence estimating equation; IEEw, independence estimating equation with inverse cluster‐period size weighting; NEME, nested‐exchangeable mixed‐effects; NEMEw, nested‐exchangeable mixed‐effects model with inverse cluster‐period size weighting.

Subsequent inference using the leave‐one‐cluster‐out jackknife variance estimator with the weighted analyses produced good CP of the 95% confidence interval for unbiased estimators of the cATE (IEEw, FEw, and EMEw models) but had slight under‐coverage for unbiased estimators of the iATE (IEE, FE, EME models) across our simulation scenarios (Figure 8). As expected, this improved CP with the jackknife variance estimator came at the cost of power (Figure 9).

In Supporting Information Appendix Section A.4.2, we included bias results from two additional simulation scenarios with informative cluster sizes, testing the amount of bias yielded by the EME and EMEw estimators for the iATE and cATE estimands. Simulation parameters were set to maximize bias in the EMEw estimator, with small values of ζ and the ICC set to the corresponding value ρ to optimize the bias in the EMEw estimator for the cATE estimand (as previously discussed in Section 5). Despite these scenarios being unrealistically tailored to yield maximally biased EMEw estimates for the cATE estimand, the EMEw estimator still remained relatively unbiased. Overall, we observe that the EMEw estimator generally yields minimal bias for the cATE estimand across many scenarios, despite not being a theoretically consistent estimator for the cATE in PB‐CRTs with informative cluster sizes.

## 7 Case Study

We reanalyzed a publicly available PB‐CRT: the JIAH trial, which explored the effects of community youth teams on a variety of outcomes among adolescent girls in rural eastern India (Bhatia et al. 2023). The JIAH trial had 28 clusters with observations from both periods of the trial. We re‐analyzed the data collected from those clusters, with reported mental health scores as the continuous outcome of interest.

The cluster‐period cell sizes in this trial varied greatly between periods within clusters (Supporting Information Appendix Section A.5). For weighted analyses, we used the inverse cluster‐period cell size weights to generate the IEEw and FEw treatment effect estimators, as described in Section 4. To our knowledge, the extension of inverse cluster‐period size weighting to analyses with variable cluster‐period size and correlated errors (EMEw and NEMEw) is not as clear and deserves additional future work. Furthermore, it is not even desirable to use the NEMEw estimator as seen in Section 6.

**FIGURE 10:**
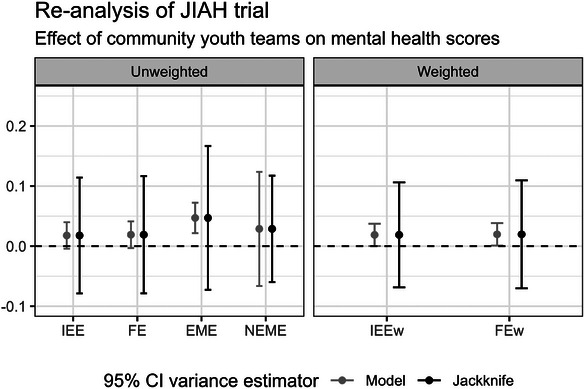
Estimates and the corresponding 95% confidence intervals as estimated using the model‐based and leave‐one‐cluster‐out jackknife variance estimators from the different unweighted and weighted models in the re‐analysis of the JIAH trial. EME, exchangeable mixed‐effects; FE, fixed‐effects; FEw, fixed‐effects model with inverse cluster‐period size weighting; IEE, independence estimating equation; IEEw, independence estimating equation with inverse cluster‐period size weighting; NEME, nested‐exchangeable mixed‐effects.

The JIAH case study estimates with corresponding 95% confidence intervals, as generated with the model‐based and leave‐one‐cluster‐out jackknife variance estimators, are presented in Figure 10. As we previously observed in our simulation results (Figure 6), the model‐based variances can yield overly narrow confidence intervals that may overstate the significance of the results. Here, we similarly observe that the model‐based variances are considerably smaller than the jackknife variance estimates in all models except for the NEME model. In contrast, the jackknife variance estimates across the different models roughly approximate the model‐based variance estimates from the NEME model. Accordingly, we suggest using the jackknife variance estimator for inference.

The results between the weighted and unweighted analyses are qualitatively similar, which may imply this trial has non‐informative cluster sizes or a homogeneous treatment effect across clusters on mental health scores. However, the subtle difference between the IEE and FE estimates as compared to the EME and NEME estimates for the iATE estimand may suggest the opposite, as the latter two estimators are not theoretically consistent for the iATE estimand. On the basis of this re‐analysis of a single dataset, we do not make a judgment on whether informative cluster sizes were present in the JIAH trial. However, the magnitudes of the iATE and cATE estimates are generally similar.

## 8 Discussion

Under the potential outcomes framework, we focus on two natural estimands of interest in the context of PB‐CRTs, the iATE and cATE. Notably, in scenarios with non‐informative cluster sizes and assuming the potential outcomes have the same marginal mean, the iATE and cATE will be equivalent, and all of the described treatment effect estimators are expected to be consistent and unbiased. Under informative cluster sizes, we demonstrate that the IEE and FE model point estimators are theoretically consistent estimators of the iATE estimand. However, unlike the IEE estimator, the FE estimator is only consistent for the iATE estimand in the presence of informative cluster sizes when cluster‐period sizes do not vary between periods within clusters. Additionally, these two estimators with inverse cluster‐period size weights, the IEEw and FEw model point estimators, are theoretically consistent estimators of the cATE estimand.

We used a simulation study to compare the performance of the different estimators in terms of bias and efficiency. As expected, the IEEw and FEw point estimators yielded empirically unbiased estimates for the cATE estimand. Across our simulation replicates, we empirically observe through the Monte Carlo variances that the FE and FEw treatment effect estimators were more efficient than the IEE and IEEw treatment effect estimators. This observation was not previously discussed in the context of CRTs and highlights the potential for FE and FEw estimators in estimand‐driven analysis of PB‐CRTs. Despite potential misspecification of the correlation structure in the FE and FEw estimators, the jackknife variance estimator roughly approximated the Monte Carlo variances and yielded proper coverage of the 95% confidence interval.

We observe that the exchangeable (EME) and nested‐exchangeable (NEME) mixed‐effects models and their inverse cluster‐period size weighted counterparts (EMEw and NEMEw) are not theoretically consistent estimators for the iATE and cATE estimands, respectively. Instead, these unweighted and weighted treatment effect estimators converge to estimands that are difficult to interpret, with cluster‐specific weights that depend on both the cluster‐period size as well as the probability limit of the model‐based ICC estimator. Therefore, in general, the ICC will dictate the estimands, and therefore the analysis of a different outcome may inadvertently change the estimand, leading to potential challenges in interpretation. However, we surprisingly demonstrate that unlike previously reported results in a P‐CRT (X. Wang et al. 2022), the unweighted and weighted treatment effect estimators with an exchangeable correlation structure (EME and EMEw) typically yield minimally biased estimates of the iATE and cATE estimands once a baseline period is included in the trial. This provides some empirical justification for the application of EME and EMEw in PB‐CRTs even under informative cluster sizes. For ease of reference, we provide in Table 2 a concise summary of the potential advantages and disadvantages of the different models explored in this article.

**TABLE 2:** Potential advantages and disadvantages of several commonly used models for estimating the individual‐average treatment effect (iATE) and cluster‐average treatment effect (cATE) estimands in PB‐CRTs when informative cluster sizes (ICS) are present.

Target	Methods	Potential advantages	Potential disadvantages
iATE	IEE	Theoretically consistent under ICS	Only uses information from the follow‐up period and may be inefficient Doesn't automatically estimate ICC
	FE	Theoretically consistent under ICS when cluster‐period sizes don't vary Uses information from both baseline and follow‐up periods, which can improve efficiency over IEE	Not theoretically consistent under ICS when cluster‐period sizes vary Doesn't automatically estimate ICC
	EME	Minimally biased under ICS Automatically estimates the ICC Uses information from both baseline and follow‐up periods, which can improve efficiency over IEE	Not theoretically consistent under ICS Doesn't automatically estimate wp‐ICC and bp‐ICC
	NEME	Automatically estimates wp‐ICC and bp‐ICC	Not theoretically consistent and potentially biased under ICS
cATE	IEEw	Theoretically consistent under ICS Weights are easy to program in standard statistical software, even when cluster‐period sizes vary	Only uses information from the follow‐up period and may be inefficient Doesn't automatically estimate ICC
	FEw	Theoretically consistent under ICS Weights are easy to program in standard statistical software, even when cluster‐period sizes vary Uses information from both baseline and follow‐up periods, which can improve efficiency over IEEw	Doesn't automatically estimate ICC
	EMEw	Minimally biased under ICS Automatically estimates ICC Uses information from both baseline and follow‐up periods, which can improve efficiency over IEE	Not theoretically consistent under ICS More complicated to program in standard statistical software (requires *WeMix* in R) Unclear how to specify the weights when cluster‐period sizes vary
	NEMEw	Automatically estimates wp‐ICC and bp‐ICC	Not theoretically consistent and potentially biased under ICS More complicated to program in standard statistical software (requires *WeMix* in R) Unclear how to specify the weights when cluster‐period sizes vary

Abbreviations: EME, exchangeable mixed‐effects; EMEw, exchangeable mixed‐effects model with inverse cluster‐period size weighting; FE, fixed‐effects; FEw, fixed‐effects model with inverse cluster‐period size weighting; IEE, independence estimating equation; IEEw, independence estimating equation with inverse cluster‐period size weighting; NEME, nested‐exchangeable mixed‐effects; NEMEw, nested‐exchangeable mixed‐effects model with inverse cluster‐period size weighting.

In many of our derivations for the convergence probability limit of the treatment effect estimators, we assume that cluster‐period sizes are equivalent between periods within clusters. However, it is possible for multi‐period CRTs to have variable cluster‐period cell sizes (although on many occasions the within‐cluster variation in cluster‐period size may not be as substantial compared to between‐cluster variation). It is straightforward to specify variable inverse cluster‐period size weights in analyses with an independence correlation structure, as is the case with the IEEw and FEw estimators, which we have demonstrated are theoretically consistent estimators for the cATE estimand. In R, such weights can be easily specified with the “weights” option in lm(). However, to our knowledge, the extension of inverse cluster‐period size weighting to analyses with variable cluster‐period size and correlated errors (EMEw and NEMEw) is unclear. Notably, specifying such weights deviates from the weighted estimating equation described by Williamson et al. (2003), which specifies weights on the cluster‐level rather than the cluster‐period‐level. Nonetheless, the FEw estimator has similar properties to the EMEw estimator, is a theoretically consistent estimator for the cATE, and can be easily implemented in most statistical software. In general, if cluster‐period sizes vary between periods within clusters, the IEEw and FEw estimators can be used instead to estimate the cATE in the analysis of PB‐CRTs with informative cluster sizes.

Many of the models explored in this article have been previously described for the analysis of PB‐CRTs by Hooper et al. (2018). Specifically, the FE, EME, and NEME estimators have been referred to as the “difference of differences,” “constrained baseline analysis with a less flexible correlation structure,” and “constrained baseline analysis with a realistic correlation structure,” respectively (Hooper et al. 2018). Under the assumption of non‐informative cluster sizes, Hooper et al. (2018) previously discouraged the use of the FE model, citing that it can fail to achieve the statistical power that was expected when using the model‐based variance estimator (Hooper et al. 2018). However, mixed‐effects models can also have an increased risk of false positives when using the model‐based variance estimator, as pointed out in the same work (Hooper et al. 2018). One of our novel contributions is to point out that, under informative cluster sizes, the FE estimators can in fact return theoretically consistent estimators for estimands defined under the potential outcomes framework and lead to efficiency improvements over the IEE estimators. From this perspective, the FE estimators should not be entirely discouraged and remain useful for the analysis of PB‐CRTs. Regardless, we suggest using the jackknife variance estimator over the model‐based variance estimator due to its ability to maintain valid inference even under informative cluster size and/or with misspecification of the correlation structure.

Hooper et al. (2018) also describe and recommend an “ANCOVA” analysis, which only analyzes the follow‐up period (in this article, J=1) with an exchangeable correlation structure and includes additional covariate adjustment for the cluster‐specific average baseline outcome (in this article, ${\bar{Y}}_{i0}=\sum_{k}Y_{i0k}/K_{i0}$). This analysis is equivalent to a covariate‐adjusted EME analysis of a P‐CRT and is always robust under arbitrary misspecification of the analysis, assuming non‐informative cluster sizes (B. Wang et al. 2023). Accordingly, the point estimator of such an “ANCOVA” analysis is equivalent to the expected EME estimator for a P‐CRT, which, as described by Wang et al. (2022) (referred to by them as the EEE estimator), will be vulnerable to accepting additional bias in weighted analyses in the presence of informative cluster sizes (X. Wang et al. 2022).

Aside from the concerns detailed in this article regarding PB‐CRT scenarios with informative cluster sizes, there are also other considerations when choosing among these different analytic methods. Notably, in terms of empirical variance, we observe that the IEE estimator can be less efficient than the FE estimator and the often minimally biased EME estimator (Supporting Information Appendix Section A.4.1). Furthermore, as described earlier in Section 3, modeling cluster intercepts as fixed in the FE and FEw estimators automatically adjusts for all cluster‐level time‐invariant covariates and potential confounding. Indeed, the FEw estimator is demonstrated here to be equivalent to a DiD estimator and estimates the cATT in the absence of randomization (Supporting Information Appendix Section A.2.4). This is in contrast to the described mixed‐effects models (EME, EMEw, NEME, NEMEw), which make an additional exogeneity assumption between their (random) cluster effects and the other model covariates (Wooldridge 2010) and can be more vulnerable to unmeasured cluster‐level time‐invariant covariate imbalance (Lee and Cheung 2024b). Recent work has also demonstrated that in contrast to the mixed‐effects model, the FE model can yield proper Type I errors in SW‐CRTs with a small number of clusters (Lee and Cheung 2024b). We conjecture this observation would hold in PB‐CRTs with a small number of clusters, although future work is needed to systematically investigate the performance characteristics of the FE model with few clusters across different outcome DGPs. Our overall standpoint is that the FE model remains a valuable tool and deserves wider recognition, particularly for the purpose of estimand‐driven analysis in PB‐CRTs.

### 8.1 Conclusions

Previous work by Wang et al. (2022) demonstrated that out of their explored estimators, only their corresponding IEEw estimator is theoretically consistent for estimating the cATE estimand in a P‐CRT design (X. Wang et al. 2022). Furthermore, they demonstrate that the commonly used exchangeable mixed‐effects model with inverse cluster‐size weights (corresponding to the EMEw) can yield unacceptably biased results in the presence of informative cluster sizes (X. Wang et al. 2022).

In extending their work to a PB‐CRT design, we demonstrate that in addition to the IEE and IEEw estimators, the FE and FEw estimators are also theoretically consistent for estimating the iATE and cATE estimands, respectively. Additionally, the FE and FEw estimators can be more efficient than the IEE and IEEw estimators in our empirical observations and are thus recommended for practice. This is especially true for the FEw estimator, which is theoretically consistent for the cATE estimand, regardless of whether cluster‐period sizes vary between periods within clusters. In contrast, the FE estimator is not theoretically consistent for the iATE estimand when cluster‐period sizes vary between periods within clusters. In such scenarios, the IEE may be more appropriate. Furthermore, we observe that despite being theoretically inconsistent for the iATE and cATE estimands, the EME and EMEw estimators can have surprisingly minimal bias even in the presence of informative cluster sizes.

Overall, the IEE, FE, and EME treatment effect estimators (and their weighted counterparts) are generally trustworthy estimators for the iATE (and cATE) in PB‐CRTs with informative cluster sizes. In contrast, the NEME and NEMEw estimators can yield unacceptably biased estimates for the iATE and cATE in the presence of informative cluster sizes. It is likely that these conclusions would extend to other multi‐period CRT designs with informative cluster‐period cell sizes, including the cluster randomized crossover and stepped wedge designs. These expansions would require a careful account of the more complex analytic options, with new derivations of the convergence results, and will be pursued in our future work.

## Conflicts of Interest

The authors declare no conflicts of interest.

## Supporting information



Supporting Information

## Data Availability

Data sharing is not applicable to this article as no new data were created or analyzed in this study. The de‐identified data analyzed in Section 7 can be accessed via the following link (https://figshare.com/s/20b46535925e26ea3da2). R codes for the simulations will be deposited to figshare by Wiley.
