# Supplementary material for: How Should Parallel Cluster Randomized Trials With a Baseline Period be Analyzed?—A Survey of Estimands and Common Estimators
Source: Biom J. 2025 Apr 29;67(3):e70052. doi: 10.1002/bimj.70052 (PMC12041842; doi:10.1002/bimj.70052)
Supplement: Supplementary file 1 — Supporting Information [file BIMJ-67-e70052-s001.pdf]

# A Web Appendix

## A.1 Estimand derivations

Here, we derive the individual-Average Treatment Effect (iATE) and cluster-Average Treatment Effect (cATE) estimands in a PB-CRT under a superpopulation framework, where $I \to \infty$.

The iATE under a finite population framework is $iATE = \frac{\sum_{i=1}^{I}\sum_{k=1}^{K_{i1}}(Y_{i1k}(1)-Y_{i1k}(0))}{\sum_{i=1}^{I}K_{i1}}$ (Kahan et al., 2024; X. Wang et al., 2022). Accordingly, the iATE estimand under an infinite superpopulation, where $I \to \infty$, is:

$$lim_{I\to\infty} iATE = lim_{I\to\infty} \frac{\sum_{i=1}^{I}\sum_{k=1}^{K_{i1}}\left(Y_{i1k}(1)-Y_{i1k}(0)\right)}{\sum_{i=1}^{I}K_{i1}}$$

$$= \frac{lim_{I\to\infty}\sum_{i=1}^{I}\sum_{k=1}^{K_{i1}}\left(Y_{i1k}(1)-Y_{i1k}(0)\right)}{lim_{I\to\infty}\sum_{i=1}^{I}K_{i1}}$$

And:

$$iATE \xrightarrow{P} \frac{E\left[\sum_{k=1}^{K_{i1}}\left(Y_{i1k}(1)-Y_{i1k}(0)\right)\right]}{E[K_{i1}]}$$

$$= E\left[\frac{1}{E[K_{i1}]}\sum_{k=1}^{K_{i1}}[Y_{i1k}(1)-Y_{i1k}(0)]\right].$$

Crucially, if $K_{i1}$ is independent of potential outcomes $\left(Y_{i1k}(1), Y_{i1k}(0)\right)$ for any $k$ (i.e., non-informative cluster sizes) and if the marginal mean of $\left(Y_{i1k}(1), Y_{i1k}(0)\right)$ is identical across $k$, then we have:

$$\frac{E\left[\sum_{k=1}^{K_{i1}}\left(Y_{i1k}(1)-Y_{i1k}(0)\right)\right]}{E[K_{i1}]} = \frac{E\left[E\left[\sum_{k=1}^{K_{i1}}\left(Y_{i1k}(1)-Y_{i1k}(0)\right)|K_{ij}\right]\right]}{E[K_{i1}]}$$

$$= \frac{E\left[\sum_{k=1}^{K_{i1}}E\left[Y_{i1k}(1)-Y_{i1k}(0)|K_{ij}\right]\right]}{E[K_{i1}]} = \frac{E\left[\sum_{k=1}^{K_{i1}}E[Y_{i1k}(1)-Y_{i1k}(0)]\right]}{E[K_{i1}]}$$

$$= \frac{E\left[K_{i1}E[Y_{i1k}(1)-Y_{i1k}(0)]\right]}{E[K_{i1}]} = \frac{E[K_{i1}]E[Y_{i1k}(1)-Y_{i1k}(0)]}{E[K_{i1}]}$$

$$= E[Y_{i1k}(1)-Y_{i1k}(0)].$$

The cATE under a finite population framework is $cATE = \frac{1}{I}\sum_{i=1}^{I}\frac{\sum_{k=1}^{K_{i1}}(Y_{i1k}(1)-Y_{i1k}(0))}{K_{i1}}$ (Kahan et al., 2024; X. Wang et al., 2022). Accordingly, the cATE estimand under a superpopulation, where $I \to \infty$, is:

$$lim_{I\to\infty} cATE = lim_{I\to\infty} \frac{1}{I}\sum\nolimits_{i=1}^{I} \frac{\sum_{k=1}^{K_{i1}}\left(Y_{i1k}(1) - Y_{i1k}(0)\right)}{K_{i1}}$$

and:

$$cATE \xrightarrow{P} E\left[\frac{\sum_{k=1}^{K_{i1}} Y_{i1k}(1)}{K_{i1}} - \frac{\sum_{k=1}^{K_{i1}} Y_{i1k}(0)}{K_{i1}}\right]$$

$$= E\left[\frac{1}{K_{i1}}\sum_{k=1}^{K_{i1}}[Y_{i1k}(1) - Y_{i1k}(0)]\right].$$

If $K_{i1}$ is independent of potential outcomes $\left(Y_{i1k}(1), Y_{i1k}(0)\right)$ for any $k$ (i.e., non-informative cluster sizes) and if the marginal mean of $\left(Y_{i1k}(1), Y_{i1k}(0)\right)$ is identical across $k$, then we can similarly demonstrate that:

$$E\left[\frac{\sum_{k=1}^{K_{i1}}\left(Y_{i1k}(1) - Y_{i1k}(0)\right)}{K_{i1}}\right] = E[Y_{i1k}(1) - Y_{i1k}(0)]\,.$$

## A.2 Complete derivations of the main results for all estimators

### A.2.1 Independence estimating equation (IEE)

Generally, the IEE estimator can be written with potential outcomes $\left(Y_{ijk}(0), Y_{i1k}(1)\right)$ for individual $k \in (1, \dots, K_{i-})$ in period $j \in (0,1)$ of cluster $i \in (1, \dots, I)$. Let $S_i$ be an indicator for whether individuals are assigned to cluster sequence $S_i = 1$:

$$\hat{\delta}_{IEE} = \left[\frac{\sum_i S_i \sum_j \sum_k Y_{i1k}(1)}{\sum_i S_i K_{i1}}\right] - \left[\frac{\sum_i (1 - S_i) \sum_j \sum_k Y_{i1k}(0)}{\sum_i (1 - S_i) K_{i1}}\right]$$

$$= \left[\frac{\sum_i S_i \sum_k Y_{i1k}(1)}{\sum_i S_i K_{i1}}\right] - \left[\frac{\sum_i (1 - S_i) \sum_k Y_{i1k}(0)}{\sum_i (1 - S_i) K_{i1}}\right].$$

We can show that this estimator is consistent and asymptotically unbiased for the iATE:

$$lim_{I \to \infty} \hat{\delta}_{IEE} = lim_{I \to \infty} \left[\frac{\sum_i S_i \sum_k Y_{i1k}(1)}{\sum_i S_i K_{i1}}\right] - lim_{I \to \infty} \left[\frac{\sum_i (1 - S_i) \sum_k Y_{i1k}(0)}{\sum_i (1 - S_i) K_{i1}}\right]$$

$$= \left[\frac{lim_{I \to \infty} \sum_i S_i \sum_k Y_{i1k}(1)}{lim_{I \to \infty} \sum_i S_i K_{i1}}\right] - \left[\frac{lim_{I \to \infty} \sum_i (1 - S_i) \sum_k Y_{i1k}(0)}{lim_{I \to \infty} \sum_i (1 - S_i) K_{i1}}\right]$$

as the number of sampled clusters $I \to \infty$, by the law of large numbers, we can show that the IEE estimator converges in probability to:

$$\hat{\delta}_{IEE} \overset{P}{\to} \left[\frac{E[\sum_k Y_{i1k}(1) \mid S_i]}{E[K_{i1} \mid S_i]}\right] - \left[\frac{E[\sum_k Y_{i1k}(0) \mid 1 - S_i]}{E[K_{i1} \mid 1 - S_i]}\right]$$

where with randomization, the sequence variable $S_i$ is independent of the potential outcomes and cluster-period sizes $S_i \perp\!\!\!\perp \Omega$, and $\Omega = \{Y_{i1k}(1), Y_{i1k}(0), K_{i1}\}_{i=1,k=1}^{I,K_{i1}}$:

$$= \left[\frac{E[\sum_k Y_{i1k}(1)]}{E[K_{i1}]}\right] - \left[\frac{E[\sum_k Y_{i1k}(0)]}{E[K_{i1}]}\right]$$

$$= E\left[\frac{1}{E[K_{i1}]} \sum_{k=1}^{K_{i1}} [Y_{i1k}(1) - Y_{i1k}(0)]\right].$$

### A.2.2 Independence estimating equation with inverse cluster-period size weighting (IEEw)

The weighted IEEw estimator can then be written as:

$$\hat{\delta}_{IEEw} = \left[\frac{\sum_i S_i \sum_j \frac{1}{K_{i1}} \sum_k Y_{i1k}(1)}{\sum_i S_i}\right] - \left[\frac{\sum_i (1 - S_i) \sum_j \frac{1}{K_{i1}} \sum_k Y_{i1k}(0)}{\sum_i (1 - S_i)}\right]$$

$$= \left[\frac{\sum_i S_i \frac{1}{K_{i1}} \sum_k Y_{i1k}(1)}{\sum_i S_i}\right] - \left[\frac{\sum_i (1-S_i) \frac{1}{K_{i1}} \sum_k Y_{i1k}(0)}{\sum_i (1-S_i)}\right]$$

where with equal allocation:

$$\hat{\delta}_{IEEw} = \left(\frac{1}{I/2}\right)\left[\sum_i S_i \frac{1}{K_{i1}} \sum_k Y_{i1k}(1) - \sum_i (1-S_i) \frac{1}{K_{i1}} \sum_k Y_{i1k}(0)\right]$$

and as the number of sampled clusters $I \to \infty$, by the law of large numbers, we can show that the IEEw estimator converges in probability to:

$$lim_{I\to\infty}\hat{\delta}_{IEEw} = \left(\frac{1}{I/2}\right) lim_{I\to\infty}\left[\sum_i S_i \frac{1}{K_{i1}} \sum_k Y_{i1k}(1) - \sum_i (1-S_i) \frac{1}{K_{i1}} \sum_k Y_{i1k}(0)\right]$$

$$= E\left[\frac{\sum_{k=1}^{K_{ij}} Y_{i1k}(1)}{K_{i1}} |S_i\right] - E\left[\frac{\sum_{k=1}^{K_{ij}} Y_{i1k}(0)}{K_{i1}} |1 - S_i\right]$$

where with randomization, the sequence variable $S_i$ is independent of the potential outcomes and cluster-period sizes $S_i \perp\!\!\!\perp \Omega$, and $\Omega = \{Y_{i1k}(1), Y_{i1k}(0), K_{i1}\}_{i=1,k=1}^{I,K_{i1}}$:

$$\hat{\delta}_{IEE} \xrightarrow{P} E\left[\frac{\sum_{k=1}^{K_{ij}} Y_{i1k}(1)}{K_{i1}} - \frac{\sum_{k=1}^{K_{ij}} Y_{i1k}(0)}{K_{i1}}\right].$$

In addition to being consistent and asymptotically unbiased for the cATE, we can also demonstrate that the IEEw estimator is unbiased for the cATE in expectation over the sampling distribution. We can formally define the set of potential outcomes for all $K_{i1}$ individuals in the sampled clusters $i = 1, \ldots, I$ as $\Omega = \{Y_{i1k}(1), Y_{i1k}(0), K_{i1}\}_{i=1,k=1}^{I,K_{i1}}$. Formally, we take the expectation of $\hat{\delta}_{IEEw}$ by treating the potential outcomes of the samples as fixed quantities and the sequence assignment (equivalent to a treatment assignment in follow-up period $j = 1$) $S_i$ as random. Therefore, conditioning the expectation on the set of sampled potential outcomes $\Omega$:

$$E[\hat{\delta}_{IEEw}|\Omega] = E\left[\frac{\sum_i S_i \frac{1}{K_{i1}} \sum_k Y_{i1k}(1)}{\sum_i S_i} \mid \Omega\right] - E\left[\frac{\sum_i (1-S_i) \frac{1}{K_{i1}} \sum_k Y_{i1k}(0)}{\sum_i (1-S_i)} \mid \Omega\right]$$

$$= \frac{1}{I/2} E\left[\sum_i S_i \frac{1}{K_{i1}} \sum_k Y_{i1k}(1) \mid \Omega\right] - \frac{1}{I/2} E\left[\sum_i (1-S_i) \frac{1}{K_{i1}} \sum_k Y_{i1k}(0) \mid \Omega\right]$$

$$= \frac{1}{I/2} \sum_i P(S_i) \frac{1}{K_{i1}} \sum_k Y_{i1k}(1) - \frac{1}{I/2} \sum_i P(1-S_i) \frac{1}{K_{i1}} \sum_k Y_{i1k}(0)$$

$$= \frac{1}{I/2} \sum_i \frac{I/2}{I} \frac{1}{K_{i1}} \sum_k Y_{i1k}(1) - \frac{1}{I/2} \sum_i \frac{I/2}{I} \frac{1}{K_{i1}} \sum_k Y_{i1k}(0)$$

$$= \frac{1}{I}\sum_i \left[\frac{1}{K_{i1}}\sum_k Y_{i1k}(1) - \frac{1}{K_{i1}}\sum_k Y_{i1k}(0)\right]$$

We assume that the sample of clusters is a simple random sample from a superpopulation of clusters. With this superpopulation framework, we have two sources of randomness, random sampling from a superpopulation of clusters and subsequent randomization of treatment assignment. With the law of total expectation:

$$E\left[E\left[\hat{\delta}_{IEEw}|\Omega\right]\right] = E\left[\hat{\delta}_{IEEw}\right]$$

$$= E\left[\frac{1}{I}\sum_i \left[\frac{1}{K_{i1}}\sum_k Y_{i1k}(1) - \frac{1}{K_{i1}}\sum_k Y_{i1k}(0)\right]\right]$$

$$= E\left[\frac{\sum_{k=1}^{K_{ij}} Y_{i1k}(1)}{K_{i1}} - \frac{\sum_{k=1}^{K_{ij}} Y_{i1k}(0)}{K_{i1}}\right].$$

## A.2.3 Fixed-effects model (FE)

The FE estimator can be written with potential outcomes $\left(Y_{ijk}(0), Y_{i1k}(1)\right)$ for individual $k \in (1, \ldots, K_{i-})$ in period $j \in (0,1)$ of cluster $i \in (1, \ldots, I)$. Let $S_i$ be an indicator for whether individuals are assigned to cluster sequence $S_i = 1$:

$$\hat{\delta}_{FE}$$

$$= \left[\frac{\left(\sum_{i=1}^{I} S_i \frac{\prod_{j=0}^{1} K_{ij}}{\sum_{j=0}^{1} K_{ij}} \sum_{j=1}^{1} \frac{1}{K_{i1}} \sum_{k=1}^{K_{i1}} Y_{i1k}(1)\right) - \left(\sum_{i=1}^{I} S_i \frac{\prod_{j=0}^{1} K_{ij}}{\sum_{j=0}^{1} K_{ij}} \sum_{j=0}^{0} \frac{1}{K_{i0}} \sum_k Y_{i0k}(0)\right)}{\sum_{i=1}^{I} S_i \frac{\prod_{j=0}^{1} K_{ij}}{\sum_{j=0}^{1} K_{ij}}}\right]$$

$$- \left[\frac{\left(\sum_{i=1}^{I}(1 - S_i) \frac{\prod_{j=0}^{1} K_{ij}}{\sum_{j=0}^{1} K_{ij}} \sum_{j=1}^{1} \frac{1}{K_{i1}} \sum_k Y_{i1k}(0)\right) - \left(\sum_{i=1}^{I}(1 - S_i) \frac{\prod_{j=0}^{1} K_{ij}}{\sum_{j=0}^{1} K_{ij}} \sum_{j=0}^{0} \frac{1}{K_{i0}} \sum_k Y_{i0k}(0)\right)}{\sum_{i=1}^{I}(1 - S_i) \frac{\prod_{j=0}^{1} K_{ij}}{\sum_{j=0}^{1} K_{ij}}}\right]$$

$$= \left[\frac{\left(\sum_{i=1}^{I} S_i \frac{K_{i0}}{\sum_{j=0}^{1} K_{ij}} \sum_{k=1}^{K_{i1}} Y_{i1k}(1)\right) - \left(\sum_{i=1}^{I} S_i \frac{K_{i1}}{\sum_{j=0}^{1} K_{ij}} \sum_k Y_{i0k}(0)\right)}{\sum_{i=1}^{I} S_i \frac{\prod_{j=0}^{1} K_{ij}}{\sum_{j=0}^{1} K_{ij}}}\right]$$

$$- \left[\frac{\left(\sum_{i=1}^{I}(1 - S_i) \frac{K_{i0}}{\sum_{j=0}^{1} K_{ij}} \sum_k Y_{i1k}(0)\right) - \left(\sum_{i=1}^{I}(1 - S_i) \frac{K_{i1}}{\sum_{j=0}^{1} K_{ij}} \sum_k Y_{i0k}(0)\right)}{\sum_{i=1}^{I}(1 - S_i) \frac{\prod_{j=0}^{1} K_{ij}}{\sum_{j=0}^{1} K_{ij}}}\right]$$

We can show that this estimator converges in probability to:

$$lim_{I\to\infty}\hat{\delta}_{FE}$$

$$= lim_{I\to\infty}\left[\frac{\left(\sum_{i=1}^{I} S_i \frac{K_{i0}}{\sum_{j=0}^{1} K_{ij}} \sum_{k=1}^{K_{i1}} Y_{i1k}(1)\right) - \left(\sum_{i=1}^{I} S_i \frac{K_{i1}}{\sum_{j=0}^{1} K_{ij}} \sum_k Y_{i0k}(0)\right)}{\sum_{i=1}^{I} S_i \frac{\prod_{j=0}^{1} K_{ij}}{\sum_{j=0}^{1} K_{ij}}}\right]$$

$$- lim_{I\to\infty}\left[\frac{\left(\sum_{i=1}^{I}(1-S_i) \frac{K_{i0}}{\sum_{j=0}^{1} K_{ij}} \sum_k Y_{i1k}(0)\right) - \left(\sum_{i=1}^{I}(1-S_i) \frac{K_{i1}}{\sum_{j=0}^{1} K_{ij}} \sum_k Y_{i0k}(0)\right)}{\sum_{i=1}^{I}(1-S_i) \frac{\prod_{j=0}^{1} K_{ij}}{\sum_{j=0}^{1} K_{ij}}}\right]$$

$$= \frac{1}{E\left[\frac{\prod_{j=0}^{1} K_{ij}}{\sum_{j=0}^{1} K_{ij}} | S_i\right]} E\left[\frac{K_{i0}}{\sum_{j=0}^{1} K_{ij}} \sum_{k=1}^{K_{i1}} Y_{i1k}(1) - \frac{K_{i1}}{\sum_{j=0}^{1} K_{ij}} \sum_k Y_{i0k}(0) \,| S_i\right]$$

$$- \frac{1}{E\left[\frac{\prod_{j=0}^{1} K_{ij}}{\sum_{j=0}^{1} K_{ij}} | 1-S_i\right]} E\left[\frac{K_{i0}}{\sum_{j=0}^{1} K_{ij}} \sum_k Y_{i1k}(0) - \frac{K_{i1}}{\sum_{j=0}^{1} K_{ij}} \sum_k Y_{i0k}(0) \,| 1-S_i\right]$$

where with randomization, the sequence variable $S_i$ is independent of the potential outcomes and cluster-period sizes $S_i \perp\!\!\!\perp \Omega$, and $\Omega = \{Y_{i1k}(1), Y_{i1k}(0), K_{i1}\}_{i=1,k=1}^{I,K_{i1}}$:

$$\hat{\delta}_{FE} \xrightarrow{P} E\left[\frac{\frac{K_{i0}}{\sum_{j=0}^{1} K_{ij}} \sum_{k=1}^{K_{i1}} Y_{i1k}(1) - \frac{K_{i1}}{\sum_{j=0}^{1} K_{ij}} \sum_k Y_{i0k}(0)}{E\left[\frac{\prod_{j=0}^{1} K_{ij}}{\sum_{j=0}^{1} K_{ij}}\right]} - \frac{\frac{K_{i0}}{\sum_{j=0}^{1} K_{ij}} \sum_k Y_{i1k}(0) - \frac{K_{i1}}{\sum_{j=0}^{1} K_{ij}} \sum_k Y_{i0k}(0)}{E\left[\frac{\prod_{j=0}^{1} K_{ij}}{\sum_{j=0}^{1} K_{ij}}\right]}\right]$$

$$= E\left[\frac{\frac{K_{i0}}{\sum_{j=0}^{1} K_{ij}} \sum_{k=1}^{K_{i1}} [Y_{i1k}(1) - Y_{i1k}(0)]}{E\left[\frac{\prod_{j=0}^{1} K_{ij}}{\sum_{j=0}^{1} K_{ij}}\right]}\right].$$

Assuming that cluster-period sizes vary between clusters but not between periods within clusters, $K_{i0} = K_{i1} = K_{i-}$, gives us:

$$= E\left[\frac{\sum_{k=1}^{K_{i1}} Y_{i1k}(1)}{E[K_{i1}]} - \frac{\sum_{k=1}^{K_{i1}} Y_{i1k}(0)}{E[K_{i1}]}\right].$$

### A.2.4 Fixed-effects model with inverse cluster-period size weighting (FEw)

When cluster-period sizes vary between periods within clusters, $K_{i0} \neq K_{i1}$,we can easily specify cluster-period-specific inverse cluster-period size weights to get the following fixed-effects model with inverse cluster-period size weights (FEw):

$$\hat{\delta}_{FEw} = \left[\frac{\left(\sum_i S_i \sum_j \frac{1}{K_{i1}} \sum_k Y_{i1k}(1)\right) - \left(\sum_i S_i \sum_j \frac{1}{K_{i0}} \sum_k Y_{i0k}(0)\right)}{\sum_i S_i}\right] - \left[\frac{\left(\sum_i (1-S_i) \sum_j \frac{1}{K_{i1}} \sum_k Y_{i1k}(0)\right) - \left(\sum_i (1-S_i) \sum_j \frac{1}{K_{i0}} \sum_k Y_{i0k}(0)\right)}{\sum_i (1-S_i)}\right]$$

$$= \left[\frac{\left(\sum_{i=1}^{I} S_i \frac{1}{K_{i1}} \sum_{k=1}^{K_{i1}} Y_{i1k}(1)\right) - \left(\sum_{i=1}^{I} S_i \frac{1}{K_{i0}} \sum_{k=1}^{K_{i0}} Y_{i0k}(0)\right)}{\sum_{i=1}^{I} S_i}\right] - \left[\frac{\left(\sum_{i=1}^{I} (1-S_i) \frac{1}{K_{i1}} \sum_{k=1}^{K_{i1}} Y_{i1k}(0)\right) - \left(\sum_{i=1}^{I} (1-S_i) \frac{1}{K_{i0}} \sum_{k=1}^{K_{i0}} Y_{i0k}(0)\right)}{\sum_{i=1}^{I} (1-S_i)}\right]$$

where with equal allocation:

$$\hat{\delta}_{FEw} = \left[\frac{\left(\sum_{i=1}^{I} S_i \frac{1}{K_{i1}} \sum_{k=1}^{K_{i1}} Y_{i1k}(1)\right) - \left(\sum_{i=1}^{I} S_i \frac{1}{K_{i0}} \sum_{k=1}^{K_{i0}} Y_{i0k}(0)\right)}{I/2}\right] - \left[\frac{\left(\sum_{i=1}^{I} (1-S_i) \frac{1}{K_{i1}} \sum_{k=1}^{K_{i1}} Y_{i1k}(0)\right) - \left(\sum_{i=1}^{I} (1-S_i) \frac{1}{K_{i0}} \sum_{k=1}^{K_{i0}} Y_{i0k}(0)\right)}{I/2}\right].$$

However, such weights deviate from Williamson's derivation with cluster-specific rather than cluster-period-specific weights. Still, inverse cluster-period size weights can be easily defined in analyses with an independence correlation structure (IEEw and FEw).

As the number of sampled clusters $I \to \infty$, by the law of large numbers, we can show that the FEw estimator converges in probability to:

$$lim_{I\to\infty} \hat{\delta}_{FEw} = lim_{I\to\infty} \left[\frac{\left(\sum_{i=1}^{I} S_i \frac{1}{K_{i1}} \sum_{k=1}^{K_{i1}} Y_{i1k}(1)\right) - \left(\sum_{i=1}^{I} S_i \frac{1}{K_{i0}} \sum_{k=1}^{K_{i0}} Y_{i0k}(0)\right)}{I/2}\right] - lim_{I\to\infty} \left[\frac{\left(\sum_{i=1}^{I} (1-S_i) \frac{1}{K_{i1}} \sum_{k=1}^{K_{i1}} Y_{i1k}(0)\right) - \left(\sum_{i=1}^{I} (1-S_i) \frac{1}{K_{i0}} \sum_{k=1}^{K_{i0}} Y_{i0k}(0)\right)}{I/2}\right]$$

$$= E\left[\frac{\sum_{k=1}^{K_{i1}} Y_{i1k}(1)}{K_{i1}} - \frac{\sum_{k=1}^{K_{i0}} Y_{i0k}(0)}{K_{i0}} | S_i\right] - E\left[\frac{\sum_{k=1}^{K_{i1}} Y_{i1k}(0)}{K_{i1}} - \frac{\sum_{k=1}^{K_{i0}} Y_{i0k}(0)}{K_{i0}} | 1 - S_i\right]$$

where with randomization, the sequence variable $S_i$ is independent of the potential outcomes and cluster-period sizes $S_i \perp\!\!\!\perp \Omega$, and $\Omega = \{Y_{i1k}(1), Y_{i1k}(0), K_{i1}\}_{i=1,k=1}^{I,K_{i1}}$:

$$\hat{\delta}_{FEw} \xrightarrow{P} E\left[\frac{\sum_{k=1}^{K_{ij}} Y_{i1k}(1)}{K_{i1}} - \frac{\sum_{k=1}^{K_{ij}} Y_{i1k}(0)}{K_{i1}}\right].$$

In addition to being consistent and asymptotically unbiased for the cATE, we can also demonstrate that the FEw estimator is unbiased for the cATE in expectation over the sampling distribution. We can formally define the set of potential outcomes for all $K_{i1}$ individuals in the sampled clusters $i = 1, \dots, I$ as $\Omega = \{Y_{i1k}(1), Y_{i1k}(0), K_{i1}\}_{i=1,k=1}^{I,K_{i1}}$. Formally, we take the expectation of $\hat{\delta}_{FEw}$ by treating the potential outcomes of the samples as fixed quantities and the sequence assignment (equivalent to a treatment assignment in follow-up period $j = 1$) $S_i$ as random. Therefore, conditioning the expectation on the set of sampled potential outcomes $\Omega$, when the randomization assumption is not met, the FEw is still an unbiased estimator for the cluster-average treatment effect on the treated (cATT):

$$E[\hat{\delta}_{FEw}|\Omega] = \left(\frac{1}{I/2}\right) E\left[\left[\left(\sum_i S_i \frac{1}{K_{i1}} \sum_k Y_{i1k}(1)\right) - \left(\sum_i S_i \frac{1}{K_{i0}} \sum_k Y_{i0k}(0)\right)\right]\right.$$
$$\left. - \left[\left(\sum_i (1 - S_i) \frac{1}{K_{i1}} \sum_k Y_{i1k}(0)\right) - \left(\sum_i (1 - S_i) \frac{1}{K_{i0}} \sum_k Y_{i0k}(0)\right)\right]\right]$$

$$= \left(\frac{1}{I/2}\right) E\left[\left(\sum_i S_i \frac{1}{K_{i1}} \sum_k Y_{i1k}(1)\right) - \left(\sum_i S_i \frac{1}{K_{i0}} \sum_k Y_{i0k}(0)\right)\right]$$
$$- \left(\frac{1}{I/2}\right) E\left[\left(\sum_i (1 - S_i) \frac{1}{K_{i1}} \sum_k Y_{i1k}(0)\right) - \left(\sum_i (1 - S_i) \frac{1}{K_{i0}} \sum_k Y_{i0k}(0)\right)\right]$$

with the parallel trends assumption:

$$= \left(\frac{1}{I/2}\right) E\left[\left(\sum_i S_i \frac{1}{K_{i1}} \sum_k Y_{i1k}(1)\right) - \left(\sum_i S_i \frac{1}{K_{i0}} \sum_k Y_{i0k}(0)\right)\right]$$
$$- \left(\frac{1}{I/2}\right) E\left[\left(\sum_i S_i \frac{1}{K_{i1}} \sum_k Y_{i1k}(0)\right) - \left(\sum_i S_i \frac{1}{K_{i0}} \sum_k Y_{i0k}(0)\right)\right]$$

Altogether:

$$E[\hat{\delta}_{FEw}|\Omega] = \left(\frac{1}{I/2}\right) E\left[\left(\sum_i S_i \frac{1}{K_{i1}} \sum_k Y_{i1k}(1)\right) - \left(\sum_i S_i \frac{1}{K_{i1}} \sum_k Y_{i1k}(0)\right)\right]$$
$$= \left(\frac{1}{I/2}\right) E\left[\sum_i S_i \frac{1}{K_{i1}} \sum_k [Y_{i1k}(1) - Y_{i1k}(0)]\right]$$

$$= \left(\frac{1}{I/2}\right)\sum_i E\left[S_i \frac{1}{K_{i1}}\sum_k [Y_{i1k}(1) - Y_{i1k}(0)]\right]$$

We assume that the sample of clusters is a simple random sample from a superpopulation of clusters. With this superpopulation framework, we have two sources of randomness, random sampling from a superpopulation of clusters and subsequent randomization of treatment assignment. With the law of total expectation:

$$E\left[E[\hat{\delta}_{FEw}|\Omega]\right] = E[\hat{\delta}_{FEw}]$$

$$= E\left[\left(\frac{1}{I/2}\right)\sum_i E\left[S_i \frac{1}{K_{i1}}\sum_k [Y_{i1k}(1) - Y_{i1k}(0)]\right]\right]$$

$$= \left(\frac{1}{I/2}\right)\left(\frac{I}{2}\right) E\left[S_i \frac{1}{K_{i1}}\sum_k [Y_{i1k}(1) - Y_{i1k}(0)]\right]$$

$$= E\left[S_i \frac{1}{K_{i1}}\sum_k [Y_{i1k}(1) - Y_{i1k}(0)]\right]$$

$$= E\left[\frac{1}{K_{i1}}\sum_k [Y_{i1k}(1) - Y_{i1k}(0)]\,|S_i = 1\right]$$

Therefore, regardless of randomization of clusters to the corresponding sequences, with the parallel trends assumption the FEw estimator produces the cluster-average treatment effect on the treated (cATT).

With randomization, the sequence variable $S_i$ is independent of the potential outcomes and cluster sizes $S_i \perp\!\!\!\perp \{Y_{i1k}(1), Y_{i1k}(0), K_{ij}\}$, and:

$$E[\hat{\delta}_{FEw}|\Omega] = \left(\frac{1}{I/2}\right)\left[\left(\sum_i E[S_i]\frac{1}{K_{i1}}\sum_k Y_{i1k}(1)\right) - \left(\sum_i E[S_i]\frac{1}{K_{i0}}\sum_k Y_{i0k}(0)\right)\right]$$
$$- \left[\left(\sum_i E[1-S_i]\frac{1}{K_{i1}}\sum_k Y_{i1k}(0)\right) - \left(\sum_i E[1-S_i]\frac{1}{K_{i0}}\sum_k Y_{i0k}(0)\right)\right]$$

$$= \left(\frac{1}{I/2}\right)\left[\left(\sum_i P(S_i)\frac{1}{K_{i1}}\sum_k Y_{i1k}(1)\right) - \left(\sum_i P(S_i)\frac{1}{K_{i0}}\sum_k Y_{i0k}(0)\right)\right]$$
$$- \left[\left(\sum_i P(1-S_i)\frac{1}{K_{i1}}\sum_k Y_{i1k}(0)\right) - \left(\sum_i P(1-S_i)\frac{1}{K_{i0}}\sum_k Y_{i0k}(0)\right)\right]$$

$$= \left(\frac{1}{I}\right)\left[\left(\sum_i \frac{1}{K_{i1}}\sum_k Y_{i1k}(1)\right) - \left(\sum_i \frac{1}{K_{i0}}\sum_k Y_{i0k}(0)\right)\right]$$
$$- \left[\left(\sum_i \frac{1}{K_{i1}}\sum_k Y_{i1k}(0)\right) - \left(\sum_i \frac{1}{K_{i0}}\sum_k Y_{i0k}(0)\right)\right]$$

$$= \left(\frac{1}{I}\right)\sum_i \frac{1}{K_{i1}}\sum_k [Y_{i1k}(1) - Y_{i1k}(0)]$$

Regardless of the presence of parallel trends. Accordingly:

$$E\left[E\left[\hat{\delta}_{FEw}|\Omega\right]\right] = E\left[\hat{\delta}_{FEw}\right]$$

$$= E\left[\frac{1}{K_{i1}}\sum_k [Y_{i1k}(1) - Y_{i1k}(0)]\right]$$

## A.2.5 Exchangeable Mixed-effects model (EME)

The exchangeable mixed-effects treatment effect (EME) point estimator can be specified based on Equation 3.9 where the diagonal terms $(D_i)$ and off-diagonal terms $(F_i)$ in the block matrix corresponding to the observations within cluster $i$ in $V^{-1}$ is:

$$D_i = \frac{1}{\sigma_w^2}\left(\frac{\sigma_w^2 + (2K_{i-} - 1)\tau_\alpha^2}{\sigma_w^2 + 2K_{i-}\tau_\alpha^2}\right)$$

$$F_i = -\frac{1}{\sigma_w^2}\left(\frac{\tau_\alpha^2}{\sigma_w^2 + 2K_{i-}\tau_\alpha^2}\right)$$

assuming that cluster-period sizes vary between clusters but not between periods within clusters, $K_{ij} = K_{i0} = K_{i1} = K_{i-}$ with $2K_{i-}$ observations within each cluster.

Accordingly, the EME estimator can be written with potential outcomes $\left(Y_{ijk}(0), Y_{i1k}(1)\right)$ for individual $k \in (1, \dots, K_{i-})$ in period $j \in (0,1)$ of cluster $i \in (1, \dots, I)$. Let $S_i$ be an indicator for whether individuals are assigned to cluster sequence $S_i = 1$.

$$\hat{\delta}_{EME} = \left\{\left(\sum_i S_i A_i\right)\left(\sum_i (1 - S_i)A_i\right)\left(\sum_i C_i\right) - \left(\sum_i S_i A_i\right)\left(\sum_i (1 - S_i)B_i\right)\left(\sum_i (1 - S_i)B_i\right) - \left(\sum_i (1 - S_i)A_i\right)\left(\sum_i S_i B_i\right)\left(\sum_i S_i B_i\right)\right\}^{-1} \times$$

$$
\left\{
\begin{array}{c}
\left[\left(\sum_i S_i B_i\right)\left(\sum_i (1-S_i)B_i\right)\right]
\begin{bmatrix}
\sum_i (1-S_i)B_i \sum_j (1-\phi_j)\frac{1}{K_{ij}}\sum_k Y_{ijk}(0) \\
+\sum_i (1-S_i)A_i \sum_j \phi_j \frac{1}{K_{ij}}\sum_k Y_{ijk}(0) \\
-\sum_i S_i B_i \sum_j (1-\phi_j)\frac{1}{K_{ij}}\sum_k Y_{ijk}(0) \\
-\sum_i S_i A_i \sum_j \phi_j \frac{1}{K_{ij}}\sum_k Y_{ijk}(1)
\end{bmatrix} \\
+\left[\left(\sum_i S_i B_i\right)\left(\sum_i S_i B_i\right)-\left(\sum_i S_i A_i\right)\left(\sum_i C_i\right)\right]
\begin{bmatrix}
\sum_i (1-S_i)B_i \sum_j (1-\phi_j)\frac{1}{K_{ij}}\sum_k Y_{ijk}(0) \\
+\sum_i (1-S_i)A_i \sum_j \phi_j \frac{1}{K_{ij}}\sum_k Y_{ijk}(0)
\end{bmatrix} \\
+\left[\left(\sum_i (1-S_i)B_i\right)\left(\sum_i (1-S_i)B_i\right)-\left(\sum_i (1-S_i)A_i\right)\left(\sum_i C_i\right)\right]
\begin{bmatrix}
-\sum_i S_i B_i \sum_j (1-\phi_j)\frac{1}{K_{ij}}\sum_k Y_{ijk}(0) \\
-\sum_i S_i A_i \sum_j \phi_j \frac{1}{K_{ij}}\sum_k Y_{ijk}(1)
\end{bmatrix} \\
-\left[\left(\sum_i (1-S_i)A_i\right)\left(\sum_i S_i B_i\right)-\left(\sum_i S_i A_i\right)\left(\sum_i (1-S_i)B_i\right)\right]
\begin{bmatrix}
\sum_i C_i \sum_j (1-\phi_j)\frac{1}{K_{ij}}\sum_k Y_{ijk}(0) \\
+\sum_i S_i B_i \sum_j \phi_j \frac{1}{K_{ij}}\sum_k Y_{ijk}(1) \\
+\sum_i (1-S_i)B_i \sum_j \phi_j \frac{1}{K_{ij}}\sum_k Y_{ijk}(0)
\end{bmatrix}
\end{array}
\right\}
$$

where:

$$A_i = K_{i2}[D_i + (K_{i2}-1)F_i] = K_{i2}\frac{1}{\sigma_w^2}\left(\frac{\sigma_w^2 + K_{i1}\tau_\alpha^2}{\sigma_w^2 + (K_{i1}+K_{i2})\tau_\alpha^2}\right)$$

$$= K_{i2}\frac{1}{\sigma_w^2}\left(\frac{1+(K_{i1}-1)\rho}{1+(K_{i1}+K_{i2}-1)\rho}\right)$$

$$B_i = K_{i1}K_{i2}F_i = -K_{i1}K_{i2}\frac{1}{\sigma_w^2}\left(\frac{\tau_\alpha^2}{\sigma_w^2 + (K_{i1}+K_{i2})\tau_\alpha^2}\right)$$

$$= -K_{i1}K_{i2}\frac{1}{\sigma_w^2}\left(\frac{\rho}{1+(K_{i1}+K_{i2}-1)\rho}\right)$$

$$C_i = K_{i1}[D_i + (K_{i1}-1)F_i] = K_{i1}\frac{1}{\sigma_w^2}\left(\frac{\sigma_w^2 + K_{i2}\tau_\alpha^2}{\sigma_w^2 + (K_{i1}+K_{i2})\tau_\alpha^2}\right)$$

$$= K_{i1}\frac{1}{\sigma_w^2}\left(\frac{1+(K_{i2}-1)\rho}{1+(K_{i1}+K_{i2}-1)\rho}\right)$$

Assuming that cluster-period sizes vary between clusters but not between periods within clusters, $K_{ij} = K_{i0} = K_{i1} = K_{i-}$, produces:

$$
\begin{aligned}
\hat{\delta}_{EME} = \Bigg\{ & \left(\sum_i S_i A_i\right)\left(\sum_i (1-S_i)A_i\right)\left(\sum_i A_i\right) \\
& - \left(\sum_i S_i A_i\right)\left(\sum_i (1-S_i)B_i\right)\left(\sum_i (1-S_i)B_i\right) \\
& - \left(\sum_i (1-S_i)A_i\right)\left(\sum_i S_i B_i\right)\left(\sum_i S_i B_i\right)\Bigg\}^{-1} \times
\end{aligned}
$$

$$
\left\{
\begin{aligned}
& \left(\sum_i S_i B_i\right)\left(\sum_i (1-S_i)B_i\right)
\begin{pmatrix}
\sum_i (1-S_i)B_i \frac{1}{K_{i-}} \sum_k Y_{i0k}(0) \\
+\sum_i (1-S_i)A_i \frac{1}{K_{i-}} \sum_k Y_{i1k}(0) \\
-\sum_i S_i B_i \frac{1}{K_{i-}} \sum_k Y_{i0k}(0) \\
-\sum_i S_i A_i \frac{1}{K_{i-}} \sum_k Y_{i1k}(1)
\end{pmatrix} \\
& + \left(\left(\sum_i S_i B_i\right)\left(\sum_i S_i B_i\right) - \left(\sum_i S_i A_i\right)\left(\sum_i A_i\right)\right)
\begin{pmatrix}
\sum_i (1-S_i)B_i \frac{1}{K_{i-}} \sum_k Y_{i0k}(0) \\
+\sum_i (1-S_i)A_i \frac{1}{K_{i-}} \sum_k Y_{i1k}(0)
\end{pmatrix} \\
& - \left(\left(\sum_i (1-S_i)B_i\right)\left(\sum_i (1-S_i)B_i\right) - \left(\sum_i (1-S_i)A_i\right)\left(\sum_i A_i\right)\right)
\begin{pmatrix}
\sum_i S_i B_i \frac{1}{K_{i-}} \sum_k Y_{i0k}(0) \\
+\sum_i S_i A_i \frac{1}{K_{i-}} \sum_k Y_{i1k}(1)
\end{pmatrix} \\
& - \left(\left(\sum_i (1-S_i)A_i\right)\left(\sum_i S_i B_i\right) - \left(\sum_i S_i A_i\right)\left(\sum_i (1-S_i)B_i\right)\right)
\begin{pmatrix}
\sum_i A_i \frac{1}{K_{i-}} \sum_k Y_{i0k}(0) \\
+\sum_i S_i B_i \frac{1}{K_{i-}} \sum_k Y_{i1k}(1) \\
+\sum_i (1-S_i)B_i \frac{1}{K_{i-}} \sum_k Y_{i1k}(0)
\end{pmatrix}
\end{aligned}
\right\}
$$

Where:

$$A_i = K_{i-}[D_i + (K_{i-} - 1)F_i] = \frac{1}{\sigma_w^2}\left(\frac{1 + (K_{i-} - 1)\rho}{1 + (2K_{i-} - 1)\rho}\right)K_{i-}$$

$$B_i = \sigma_w^2 K_{i-}^2 F_i = -\frac{1}{\sigma_w^2}\left(\frac{\rho}{1 + (2K_{i-} - 1)\rho}\right)K_{i-}^2$$

with the model-based intracluster correlation coefficient given by $\rho = \frac{\tau_\alpha^2}{\tau_\alpha^2 + \sigma_w^2}$.

We can demonstrate that this estimator is consistent and asymptotically unbiased for the iATE with randomization where the sequence variable $S_i$ is independent of the potential outcomes and cluster-period sizes $S_i \perp\!\!\!\perp \Omega$, and $\Omega = \{Y_{i1k}(1), Y_{i1k}(0), K_{i1}\}_{i=1,k=1}^{I,K_{i1}}$:

:

$$lim_{I\to\infty} \hat{\delta}_{EME} =$$

$$\frac{(E[A_i]E[A_i] - E[B_i]E[B_i])E\left[A_i \frac{1}{K_{i-}} \sum_k [Y_{i1k}(1) - Y_{i1k}(0)]\right]}{E[A_i]E[A_i]E[A_i] - \left(\frac{1}{2}\right) E[A_i]E[B_i]E[B_i] - \left(\frac{1}{2}\right) E[A_i]E[B_i]E[B_i]}$$

$$= \frac{E\left[A_i \frac{1}{K_{i-}} \sum_k [Y_{i1k}(1) - Y_{i1k}(0)]\right]}{E[A_i]}$$

$$= E\left[\frac{A_i/K_{i-}}{E[A_i]} \sum_k [Y_{i1k}(1) - Y_{i1k}(0)]\right].$$

Recall:

$$A_i = K_{i-}[D_i + (K_{i-} - 1)F_i] = \frac{1}{\sigma_w^2}\left(\frac{1 + (K_{i-} - 1)\rho}{1 + (2K_{i-} - 1)\rho}\right)K_{i-}$$

Altogether, we demonstrate that the EME estimator converges in probability to the following estimand (which may be more challenging to interpret given the dependence on the unknown $\rho$):

$$\hat{\delta}_{EME} \xrightarrow{P} \frac{E\left[\left(\frac{1 + (K_{i-} - 1)\rho}{1 + (2K_{i-} - 1)\rho}\right)\sum_k [Y_{i1k}(1) - Y_{i1k}(0)]\right]}{E\left[\frac{1 + (K_{i-} - 1)\rho}{1 + (2K_{i-} - 1)\rho}\right]}.$$

In general, we can show:

$$\hat{\delta}_{ME} \xrightarrow{P} E\left[\frac{A_i/K_{i-}}{E[A_i]} \sum_k [Y_{i1k}(1) - Y_{i1k}(0)]\right]$$

but with different model-specific values of $A_i$ for any of the mixed-effects models explored here (EME, EMEw, NEME, and NEMEw).

### A.2.6 Exchangeable Mixed-effects model with inverse cluster-period size weighting (EMEw)

The weighted EMEw estimator can be specified similarly to the unweighted EME estimator, but with the diagonal terms $(D_i)$ and off-diagonal terms $(F_i)$ in the block matrices of the inverted weighted correlation structure $W_i^{-1}$ corresponding to the observations within cluster $i$ specified as:

$$D_i = \frac{1}{(K_{i-})\sigma_w^2}\left(\frac{(K_{i-})\sigma_w^2 + (2K_{i-} - 1)(K_{i-})\tau_\alpha^2}{(K_{i-})\sigma_w^2 + 2K_{i-}(K_{i-})\tau_\alpha^2}\right) = \frac{1}{(K_{i-})\sigma_w^2}\left(\frac{\sigma_w^2 + (2K_{i-} - 1)\tau_\alpha^2}{\sigma_w^2 + 2K_{i-}\tau_\alpha^2}\right)$$

$$F_i = -\frac{1}{(K_{i-})\sigma_w^2}\left(\frac{(K_{i-})\tau_\alpha^2}{(K_{i-})\sigma_w^2 + 2K_{i-}\tau_\alpha^2(K_{i-})}\right) = -\frac{1}{(K_{i-})\sigma_w^2}\left(\frac{\tau_\alpha^2}{\sigma_w^2 + 2K_{i-}\tau_\alpha^2}\right)$$

As a result, exchangeable mixed-effects model with inverse cluster-period size weighting (EMEw) estimator has an identical estimator to the EME estimator, but with a different weighted specification of $A_i$ and $B_i$:

$$A_i = K_{i-}\left[\frac{1}{(K_{i-})\sigma_w^2}\left(\frac{\sigma_w^2 + (2K_{i-} - 1)\tau_\alpha^2}{\sigma_w^2 + 2K_{i-}\tau_\alpha^2}\right) - (K_{i-} - 1)\frac{1}{(K_{i-})\sigma_w^2}\left(\frac{\tau_\alpha^2}{\sigma_w^2 + 2K_{i-}\tau_\alpha^2}\right)\right]$$

$$= \frac{1}{\sigma_w^2}\left(\frac{\sigma_w^2 + (2K_{i-} - 1)\tau_\alpha^2 - (K_{i-} - 1)\tau_\alpha^2}{\sigma_w^2 + 2K_{i-}\tau_\alpha^2}\right)$$

$$= \frac{1}{\sigma_w^2}\left(\frac{\sigma_w^2 + K_{i-}\tau_\alpha^2}{\sigma_w^2 + 2K_{i-}\tau_\alpha^2}\right)$$

$$= \frac{1}{\sigma_w^2}\left(\frac{\sigma_w^2 + K_{i-}\tau_\alpha^2}{\sigma_w^2 + 2K_{i-}\tau_\alpha^2}\right)$$

$$= \frac{1}{\sigma_w^2}\left(\frac{\sigma_w^2 + \tau_\alpha^2 + (K_{i-} - 1)\tau_\alpha^2}{\sigma_w^2 + \tau_\alpha^2 + (2K_{i-} - 1)\tau_\alpha^2}\right)$$

$$= \frac{1}{\sigma_w^2}\left(\frac{1 + (K_{i-} - 1)\left(\frac{\tau_\alpha^2}{\sigma_w^2 + \tau_\alpha^2}\right)}{1 + (2K_{i-} - 1)\left(\frac{\tau_\alpha^2}{\sigma_w^2 + \tau_\alpha^2}\right)}\right)$$

$$= \frac{1}{\sigma_w^2}\left(\frac{1 + (K_{i-} - 1)\rho}{1 + (2K_{i-} - 1)\rho}\right)$$

and

$$B_i = -\frac{K_{i-}^2}{(K_{i-})\sigma_w^2}\left(\frac{\tau_\alpha^2}{\sigma_w^2 + 2K_{i-}\tau_\alpha^2}\right)$$

$$= -\frac{K_{i-}}{\sigma_w^2}\left(\frac{\tau_\alpha^2}{\sigma_w^2 + 2K_{i-}\tau_\alpha^2}\right)$$

$$= -K_{i-}\frac{1}{\sigma_w^2}\left(\frac{\rho}{1 + (2K_{i-} - 1)\rho}\right)$$

assuming that cluster-period sizes vary between clusters but not between periods within clusters, $K_{ij} = K_{i0} = K_{i1} = K_{i-}$, with the model-based intracluster correlation coefficient defined as $\rho = \frac{\tau_\alpha^2}{\tau_\alpha^2 + \sigma_w^2}$.

Therefore, the EMEw estimator converges in probability to the following estimand:

$$\hat{\delta}_{EMEw} \xrightarrow{P} E\left[\frac{\left(\frac{1 + (K_{i-} - 1)\rho}{1 + (2K_{i-} - 1)\rho}\right)}{E\left[\left(\frac{1 + (K_{i-} - 1)\rho}{1 + (2K_{i-} - 1)\rho}\right)\right]}\left(\frac{1}{K_{i-}}\sum_k [Y_{i1k}(1) - Y_{i1k}(0)]\right)\right]$$

## A.2.7 Nested Exchangeable Mixed-effects model (NEME)

The nested exchangeable mixed-effects treatment effect (NEME) point estimator can be specified based on Equation 3.12. Assuming that cluster-period sizes vary between clusters

but not between periods within clusters, $K_{ij} = K_{i0} = K_{i1} = K_{i-}$ with $2K_{i-}$ observations within each cluster, the correlation structure is then $\check{V} = \mathrm{I}_I \otimes R_i^{NEME}$, where:

$$R_i^{NEME} = \begin{pmatrix} R_{i1}^{NEME} & R_{i2}^{NEME} \\ R_{i3}^{NEME} & R_{i4}^{NEME} \end{pmatrix},$$

with block matrices:

$$R_{i1}^{NEME} = R_{i4}^{NEME} = \left(\mathrm{I}_{K_{i-}}\sigma_w^2 + \mathrm{J}_{K_{i-}}(\tau_\alpha^2 + \tau_\gamma^2)\right)$$
$$= \begin{pmatrix} \sigma_w^2 + \tau_\alpha^2 + \tau_\gamma^2 & \tau_\alpha^2 + \tau_\gamma^2 & \cdots & \tau_\alpha^2 + \tau_\gamma^2 \\ \tau_\alpha^2 + \tau_\gamma^2 & \sigma_w^2 + \tau_\alpha^2 + \tau_\gamma^2 & \cdots & \tau_\alpha^2 + \tau_\gamma^2 \\ \vdots & \vdots & \ddots & \vdots \\ \tau_\alpha^2 + \tau_\gamma^2 & \tau_\alpha^2 + \tau_\gamma^2 & \cdots & \sigma_w^2 + \tau_\alpha^2 + \tau_\gamma^2 \end{pmatrix},$$

$$R_{i2}^{NEME} = R_{i3}^{NEME} = \left(\mathrm{J}_{K_{i-}}\tau_\alpha^2\right) = \begin{pmatrix} \tau_\alpha^2 & \tau_\alpha^2 & \cdots & \tau_\alpha^2 \\ \tau_\alpha^2 & \tau_\alpha^2 & \cdots & \tau_\alpha^2 \\ \vdots & \vdots & \ddots & \vdots \\ \tau_\alpha^2 & \tau_\alpha^2 & \cdots & \tau_\alpha^2 \end{pmatrix}$$

where $\mathrm{I}_{K_{i-}}$ is a $K_{i-}$ by $K_{i-}$ dimension identity matrix and $\mathrm{J}_{K_{i-}}$ is a $K_{i-}$ by $K_{i-}$ dimension matrix of ones. Accordingly, $\check{V}^{-1} = \mathrm{I}_I \otimes (R_i^{NEME})^{-1}$, where:

$$(R_i^{NEME})^{-1} =$$
$$\begin{pmatrix} [R_{i1}^{NEME} - R_{i2}^{NEME}(R_{i1}^{NEME})^{-1}R_{i2}^{NEME}]^{-1} & -[R_{i1}^{NEME} - R_{i2}^{NEME}(R_{i1}^{NEME})^{-1}R_{i2}^{NEME}]^{-1}R_{i2}^{NEME}(R_{i1}^{NEME})^{-1} \\ -[R_{i1}^{NEME} - R_{i2}^{NEME}(R_{i1}^{NEME})^{-1}R_{i2}^{NEME}]^{-1}R_{i2}^{NEME}(R_{i1}^{NEME})^{-1} & [R_{i1}^{NEME} - R_{i2}^{NEME}(R_{i1}^{NEME})^{-1}R_{i2}^{NEME}]^{-1} \end{pmatrix}$$

We define:

$$[R_{i1}^{NEME} - R_{i2}^{NEME}(R_{i1}^{NEME})^{-1}R_{i2}^{NEME}]^{-1} = \left(\mathrm{I}_{K_{i-}}(D_i - F_i) + \mathrm{J}_{K_{i-}}(F_i)\right)$$

where the diagonal terms ($D_i$) and off-diagonal terms ($F_i$) are:

$$D_i = \frac{1}{\sigma_w^2}\left(\frac{\sigma_w^2 + (K_{i-} - 1)\left[\left(\tau_\alpha^2 + \tau_\gamma^2\right) - \frac{(K_{i-})(\tau_\alpha^2)^2}{\sigma_w^2 + (K_{i-})\left(\tau_\alpha^2 + \tau_\gamma^2\right)}\right]}{\sigma_w^2 + (K_{i-})\left[\left(\tau_\alpha^2 + \tau_\gamma^2\right) - \frac{(K_{i-})(\tau_\alpha^2)^2}{\sigma_w^2 + (K_{i-})\left(\tau_\alpha^2 + \tau_\gamma^2\right)}\right]}\right)$$

$$F_i = -\frac{1}{\sigma_w^2}\left(\frac{\left(\tau_\alpha^2 + \tau_\gamma^2\right) - \frac{(K_{i-})(\tau_\alpha^2)^2}{\sigma_w^2 + (K_{i-})\left(\tau_\alpha^2 + \tau_\gamma^2\right)}}{\sigma_w^2 + (K_{i-})\left[\left(\tau_\alpha^2 + \tau_\gamma^2\right) - \frac{(K_{i-})(\tau_\alpha^2)^2}{\sigma_w^2 + (K_{i-})\left(\tau_\alpha^2 + \tau_\gamma^2\right)}\right]}\right).$$

Subsequently, we define:

$$-[R_{i1}^{NEME} - R_{i2}^{NEME}(R_{i1}^{NEME})^{-1}R_{i2}^{NEME}]^{-1}R_{i2}^{NEME}(R_{i1}^{NEME})^{-1} = \mathrm{J}_{K_{i-}}(G_i)$$

where:

$$G_i = -\left(\frac{\tau_\alpha^2}{\sigma_w^2 + (K_{i-})(\tau_\alpha^2 + \tau_\gamma^2)}\right)\left(\frac{1}{\sigma_w^2 + (K_{i-})\left[(\tau_\alpha^2 + \tau_\gamma^2) - \frac{(K_{i-})(\tau_\alpha^2)^2}{\sigma_w^2 + (K_{i-})(\tau_\alpha^2 + \tau_\gamma^2)}\right]}\right)$$

$$= -\left(\frac{\tau_\alpha^2}{\sigma_w^2 + (K_{i-})(\tau_\alpha^2 + \tau_\gamma^2)}\right)\left(\frac{\sigma_w^2 + (K_{i-})(\tau_\alpha^2 + \tau_\gamma^2)}{\left(\sigma_w^2 + (K_{i-})(\tau_\alpha^2 + \tau_\gamma^2)\right)^2 - (K_{i-})^2(\tau_\alpha^2)^2}\right)$$

$$= -\left(\frac{\tau_\alpha^2}{\left(\sigma_w^2 + (K_{i-})(\tau_\alpha^2 + \tau_\gamma^2)\right)^2 - (K_{i-})^2(\tau_\alpha^2)^2}\right)$$

Accordingly, the NEME estimator can be written with potential outcomes $\left(Y_{ijk}(0), Y_{i1k}(1)\right)$ for individual $k \in (1, \dots, K_{i-})$ in period $j \in (0,1)$ of cluster $i \in (1, \dots, I)$. Let $S_i$ be an indicator for whether individuals are assigned to cluster sequence $S_i = 1$. The resulting, nested exchangeable mixed-effects model (NEME) estimator has an identical estimator to the EME estimator, but with a different weighted specification of $A_i$ and $B_i$:

$$A_i = K_{i-}[D_i + (K_{i-} - 1)F_i]$$

$$= K_{i-}\left[\frac{1}{\sigma_w^2}\left(\frac{\sigma_w^2 + (K_{i-} - 1)\left[(\tau_\alpha^2 + \tau_\gamma^2) - \frac{(K_{i-})(\tau_\alpha^2)^2}{\sigma_w^2 + (K_{i-})(\tau_\alpha^2 + \tau_\gamma^2)}\right]}{\sigma_w^2 + (K_{i-})\left[(\tau_\alpha^2 + \tau_\gamma^2) - \frac{(K_{i-})(\tau_\alpha^2)^2}{\sigma_w^2 + (K_{i-})(\tau_\alpha^2 + \tau_\gamma^2)}\right]}\right) - \frac{1}{\sigma_w^2}\left(\frac{(K_{i-} - 1)\left[(\tau_\alpha^2 + \tau_\gamma^2) - \frac{(K_{i-})(\tau_\alpha^2)^2}{\sigma_w^2 + (K_{i-})(\tau_\alpha^2 + \tau_\gamma^2)}\right]}{\sigma_w^2 + (K_{i-})\left[(\tau_\alpha^2 + \tau_\gamma^2) - \frac{(K_{i-})(\tau_\alpha^2)^2}{\sigma_w^2 + (K_{i-})(\tau_\alpha^2 + \tau_\gamma^2)}\right]}\right)\right]$$

$$= K_{i-}\left(\frac{1}{\sigma_w^2 + (K_{i-})\left[(\tau_\alpha^2 + \tau_\gamma^2) - \frac{(K_{i-})(\tau_\alpha^2)^2}{\sigma_w^2 + (K_{i-})(\tau_\alpha^2 + \tau_\gamma^2)}\right]}\right)$$

$$= K_{i-}\left(\frac{\sigma_w^2 + (K_{i-})(\tau_\alpha^2 + \tau_\gamma^2)}{\left(\sigma_w^2 + (K_{i-})(\tau_\alpha^2 + \tau_\gamma^2)\right)^2 - (K_{i-})^2(\tau_\alpha^2)^2}\right)$$

which can be further written in terms of the within-period ICC $\left(\rho_{wp} = \frac{\tau_\alpha^2 + \tau_\gamma^2}{\tau_\alpha^2 + \tau_\gamma^2 + \sigma_w^2}\right)$ and between-period ICC $\left(\rho_{bp} = \frac{\tau_\alpha^2}{\tau_\alpha^2 + \tau_\gamma^2 + \sigma_w^2}\right)$:

$$= K_{i-}\left(\frac{1+(K_{i-}-1)\rho_{wp}}{\left[\left(1+(K_{i-}-1)\rho_{wp}\right)^2-(K_{i-})^2\rho_{bp}^2\right]\left(\tau_\alpha^2+\tau_\gamma^2+\sigma_w^2\right)}\right)$$

and:

$$B_i = K_{i-}^2 G_i$$

$$= -K_{i-}^2\left(\frac{\tau_\alpha^2}{\left(\sigma_w^2+(K_{i-})\left(\tau_\alpha^2+\tau_\gamma^2\right)\right)^2-(K_{i-})^2(\tau_\alpha^2)^2}\right)$$

Therefore, the NEME estimator converges in probability to the following estimand:

$$\hat{\delta}_{NEME} \xrightarrow{P} E\left[\frac{\left(\frac{1+(K_{i-}-1)\rho_{wp}}{\left(1+(K_{i-}-1)\rho_{wp}\right)^2-(K_{i-})^2\rho_{bp}^2}\right)}{E\left[\left(\frac{1+(K_{i-}-1)\rho_{wp}}{\left(1+(K_{i-}-1)\rho_{wp}\right)^2-(K_{i-})^2\rho_{bp}^2}\right)K_{i-}\right]}\sum_k\left[Y_{i1k}(1)-Y_{i1k}(0)\right]\right]$$

## A.2.8 Nested Exchangeable Mixed-effects model with inverse cluster-period size weighting (NEMEw)

The weighted NEMEw estimator can be specified similarly to the unweighted NEME estimator, but with the following terms

$$D_i = \frac{1}{(K_{i-})\sigma_w^2}\left(\frac{\sigma_w^2+(K_{i-}-1)\left[\left(\tau_\alpha^2+\tau_\gamma^2\right)-\frac{(K_{i-})(\tau_\alpha^2)^2}{\sigma_w^2+(K_{i-})\left(\tau_\alpha^2+\tau_\gamma^2\right)}\right]}{\sigma_w^2+(K_{i-})\left[\left(\tau_\alpha^2+\tau_\gamma^2\right)-\frac{(K_{i-})(\tau_\alpha^2)^2}{\sigma_w^2+(K_{i-})\left(\tau_\alpha^2+\tau_\gamma^2\right)}\right]}\right)$$

$$F_i = -\frac{1}{(K_{i-})\sigma_w^2}\left(\frac{\left(\tau_\alpha^2+\tau_\gamma^2\right)-\frac{(K_{i-})(\tau_\alpha^2)^2}{\sigma_w^2+(K_{i-})\left(\tau_\alpha^2+\tau_\gamma^2\right)}}{\sigma_w^2+(K_{i-})\left[\left(\tau_\alpha^2+\tau_\gamma^2\right)-\frac{(K_{i-})(\tau_\alpha^2)^2}{\sigma_w^2+(K_{i-})\left(\tau_\alpha^2+\tau_\gamma^2\right)}\right]}\right)$$

$$G_i = -\frac{1}{(K_{i-})}\left(\frac{\tau_\alpha^2}{\sigma_w^2+(K_{i-})\left(\tau_\alpha^2+\tau_\gamma^2\right)}\right)\left(\frac{1}{\sigma_w^2+(K_{i-})\left[\left(\tau_\alpha^2+\tau_\gamma^2\right)-\frac{(K_{i-})(\tau_\alpha^2)^2}{\sigma_w^2+(K_{i-})\left(\tau_\alpha^2+\tau_\gamma^2\right)}\right]}\right)$$

$$= -\frac{1}{(K_{i-})}\left(\frac{\tau_\alpha^2}{\left(\sigma_w^2+(K_{i-})\left(\tau_\alpha^2+\tau_\gamma^2\right)\right)^2-(K_{i-})^2(\tau_\alpha^2)^2}\right)$$

As a result, nested exchangeable mixed-effects model with inverse cluster-period size weighting (NEMEw) estimator has an identical estimator to the NEME estimator, but with a different weighted specification of $A_i$ and $B_i$:

$$A_i = K_{i-}[D_i + (K_{i-} - 1)F_i]$$

$$= \frac{1}{\sigma_w^2 + (K_{i-})\left[\left(\tau_\alpha^2 + \tau_\gamma^2\right) - \frac{(K_{i-})(\tau_\alpha^2)^2}{\sigma_w^2 + (K_{i-})\left(\tau_\alpha^2 + \tau_\gamma^2\right)}\right]}$$

$$= \frac{\sigma_w^2 + (K_{i-})\left(\tau_\alpha^2 + \tau_\gamma^2\right)}{\left(\sigma_w^2 + (K_{i-})\left(\tau_\alpha^2 + \tau_\gamma^2\right)\right)^2 - (K_{i-})^2(\tau_\alpha^2)^2}$$

which can be further written in terms of the within-period ICC $\left(\rho_{wp} = \frac{\tau_\alpha^2+\tau_\gamma^2}{\tau_\alpha^2+\tau_\gamma^2+\sigma_w^2}\right)$ and between-period ICC $\left(\rho_{bp} = \frac{\tau_\alpha^2}{\tau_\alpha^2+\tau_\gamma^2+\sigma_w^2}\right)$:

$$= \frac{1 + (K_{i-} - 1)\rho_{wp}}{\left[\left(1 + (K_{i-} - 1)\rho_{wp}\right)^2 - (K_{i-})^2\rho_{bp}^2\right]\left(\tau_\alpha^2 + \tau_\gamma^2 + \sigma_w^2\right)}$$

and:

$$B_i = K_{i-}^2 G_i$$

$$= -K_{i-}\left(\frac{\tau_\alpha^2}{\sigma_w^2 + (K_{i-})\left(\tau_\alpha^2 + \tau_\gamma^2\right)}\right)\left(\frac{1}{\sigma_w^2 + (K_{i-})\left[\left(\tau_\alpha^2 + \tau_\gamma^2\right) - \frac{(K_{i-})(\tau_\alpha^2)^2}{\sigma_w^2 + (K_{i-})\left(\tau_\alpha^2 + \tau_\gamma^2\right)}\right]}\right)$$

$$= -K_{i-}\left(\frac{\tau_\alpha^2}{\left(\sigma_w^2 + (K_{i-})\left(\tau_\alpha^2 + \tau_\gamma^2\right)\right)^2 - (K_{i-})^2(\tau_\alpha^2)^2}\right)$$

assuming that cluster-period sizes vary between clusters but not between periods within clusters, $K_{ij} = K_{i0} = K_{i1} = K_{i-}$ .

Therefore, the NEMEw estimator converges in probability to the following estimand:

$$\hat{\delta}_{NEMEw} \overset{P}{\to} E\left[\frac{\left(\frac{1 + (K_{i-} - 1)\rho_{wp}}{\left(1 + (K_{i-} - 1)\rho_{wp}\right)^2 - (K_{i-})^2\rho_{bp}^2}\right)}{E\left[\frac{1 + (K_{i-} - 1)\rho_{wp}}{\left(1 + (K_{i-} - 1)\rho_{wp}\right)^2 - (K_{i-})^2\rho_{bp}^2}\right]}\left(\frac{1}{K_{i-}}\sum_k [Y_{i1k}(1) - Y_{i1k}(0)]\right)\right]$$

## A.3 Evaluation of the EMEw estimator

### A.3.1 Derivation of ICC for maximum bias

Here, we include the derivations for the ICC value that yields maximum bias in the EMEw estimator. As stated in Section 5 of the main paper, the EMEw estimator can be interpreted as a weighted average of the cluster-specific cATE estimands, with weights $\lambda_{EMEw}$. To obtain further insights, we assume there are two subpopulations $u = 1,2$ with fixed cluster-period sizes $K_{i-,1}$ and $K_{i-,2}$ and corresponding weights $\lambda_{EMEw,1}$ and $\lambda_{EMEw,2}$.

The maximum amount of bias in the EMEw estimator for cATE estimand relative to the ICC $\rho$ occurs when there is the maximum amount of difference between the estimand weights of the two subpopulations in the EMEw estimator $(\lambda_{EMEw,1} - \lambda_{EMEw,2})$. Accordingly:

$$\lambda_{EMEw,1} - \lambda_{EMEw,2} = \frac{\left(\frac{1+(K_{i-,1}-1)\rho}{1+(2K_{i-,1}-1)\rho} - \frac{1+(K_{i-,2}-1)\rho}{1+(2K_{i-,2}-1)\rho}\right)}{E\left[\left(\frac{1+(K_{i-}-1)\rho}{1+(2K_{i-}-1)\rho}\right)\right]}$$

This difference $(\lambda_{EMEw,1} - \lambda_{EMEw,2})$ is then maximized for values of $\rho$ where $\frac{d(\lambda_{EMEw,1}-\lambda_{EMEw,2})}{d\rho} = 0$. Accordingly:

$$\frac{d(\lambda_{EMEw,1} - \lambda_{EMEw,2})}{d\rho}$$

$$= \frac{d}{d\rho}\left(E\left[\left(\frac{1+(K_{i-}-1)\rho}{1+(2K_{i-}-1)\rho}\right)\right]\right)^{-1}\left(\frac{1+(K_{i-,1}-1)\rho}{1+(2K_{i-,1}-1)\rho} - \frac{1+(K_{i-,2}-1)\rho}{1+(2K_{i-,2}-1)\rho}\right) + \left(E\left[\left(\frac{1+(K_{i-}-1)\rho}{1+(2K_{i-}-1)\rho}\right)\right]\right)^{-1}\frac{d}{d\rho}\left(\frac{1+(K_{i-,1}-1)\rho}{1+(2K_{i-,1}-1)\rho} - \frac{1+(K_{i-,2}-1)\rho}{1+(2K_{i-,2}-1)\rho}\right)$$

$$= -\left(E\left[\left(\frac{1+(K_{i-}-1)\rho}{1+(2K_{i-}-1)\rho}\right)\right]\right)^{-2} E\left[\frac{-K_{i-}}{(1+(2K_{i-}-1)\rho)^2}\right]\left(\frac{1+(K_{i-,1}-1)\rho}{1+(2K_{i-,1}-1)\rho} - \frac{1+(K_{i-,2}-1)\rho}{1+(2K_{i-,2}-1)\rho}\right) + \left(E\left[\left(\frac{1+(K_{i-}-1)\rho}{1+(2K_{i-}-1)\rho}\right)\right]\right)^{-1}\left(\frac{-K_{i-.1}}{(1+(2K_{i-.1}-1)\rho)^2} - \frac{-K_{i-,2}}{(1+(2K_{i-,2}-1)\rho)^2}\right)$$

which we set $= 0$. Solving for $\rho$:

$$\left(E\left[\left(\frac{1+(K_{i-}-1)\rho}{1+(2K_{i-}-1)\rho}\right)\right]\right)^{-1} E\left[\frac{K_{i-}}{(1+(2K_{i-}-1)\rho)^2}\right]\left(\frac{1+\left(K_{i-,1}-1\right)\rho}{1+\left(2K_{i-,1}-1\right)\rho} - \frac{1+\left(K_{i-,2}-1\right)\rho}{1+\left(2K_{i-,2}-1\right)\rho}\right) = \left(\frac{K_{i-.1}}{(1+(2K_{i-.1}-1)\rho)^2} - \frac{K_{i-,2}}{\left(1+\left(2K_{i-,2}-1\right)\rho\right)^2}\right)$$

$$E\left[\frac{K_{i-}}{(1+(2K_{i-}-1)\rho)^2}\right]\left(\frac{1+\left(K_{i-,1}-1\right)\rho}{1+\left(2K_{i-,1}-1\right)\rho} - \frac{1+\left(K_{i-,2}-1\right)\rho}{1+\left(2K_{i-,2}-1\right)\rho}\right) = E\left[\left(\frac{1+(K_{i-}-1)\rho}{1+(2K_{i-}-1)\rho}\right)\right]\left(\frac{K_{i-.1}}{(1+(2K_{i-.1}-1)\rho)^2} - \frac{K_{i-,2}}{\left(1+\left(2K_{i-,2}-1\right)\rho\right)^2}\right)$$

$$\left(P(u=1)\frac{K_{i-.1}}{(1+(2K_{i-.1}-1)\rho)^2} + P(u=2)\frac{K_{i-,2}}{\left(1+\left(2K_{i-,2}-1\right)\rho\right)^2}\right)\left(\frac{1+\left(K_{i-,1}-1\right)\rho}{1+\left(2K_{i-,1}-1\right)\rho} - \frac{1+\left(K_{i-,2}-1\right)\rho}{1+\left(2K_{i-,2}-1\right)\rho}\right)$$
$$= \left(P(u=1)\frac{1+\left(K_{i-,1}-1\right)\rho}{1+\left(2K_{i-,1}-1\right)\rho} + P(u=2)\frac{1+\left(K_{i-,2}-1\right)\rho}{1+\left(2K_{i-,2}-1\right)\rho}\right)\left(\frac{K_{i-.1}}{(1+(2K_{i-.1}-1)\rho)^2} - \frac{K_{i-,2}}{\left(1+\left(2K_{i-,2}-1\right)\rho\right)^2}\right)$$

$$\left(\frac{K_{i-,2}}{\left(1+\left(2K_{i-,2}-1\right)\rho\right)^2}\right)\left(\frac{1+\left(K_{i-,1}-1\right)\rho}{1+\left(2K_{i-,1}-1\right)\rho}\right) = \left(\frac{K_{i-.1}}{(1+(2K_{i-.1}-1)\rho)^2}\right)\left(\frac{1+\left(K_{i-,2}-1\right)\rho}{1+\left(2K_{i-,2}-1\right)\rho}\right)$$

$$\frac{K_{i-,2}\left(1+\left(K_{i-,1}-1\right)\rho\right)}{1+\left(2K_{i-,2}-1\right)\rho} = \frac{K_{i-,1}\left(1+\left(K_{i-,2}-1\right)\rho\right)}{1+\left(2K_{i-,1}-1\right)\rho}$$

$$K_{i-,2}\left(1+\left(K_{i-,1}-1\right)\rho\right)\left(1+\left(2K_{i-,1}-1\right)\rho\right) = K_{i-,1}\left(1+\left(K_{i-,2}-1\right)\rho\right)\left(1+\left(2K_{i-,2}-1\right)\rho\right)$$

$$K_{i-,2}\left(2K_{i-,1}^2\rho^2 + (1-\rho)^2\right) = K_{i-,1}\left(2K_{i-,2}^2\rho^2 + (1-\rho)^2\right)$$

$$(1-\rho)^2\left(K_{i-,2} - K_{i-,1}\right) = \rho^2\left(2K_{i-,1}K_{i-,2}^2 - 2K_{i-,1}^2K_{i-,2}\right)$$

$$(1-\rho)\sqrt{K_{i-,2} - K_{i-,1}} = \rho\sqrt{2\left(K_{i-,2} - K_{i-,1}\right)K_{i-,1}K_{i-,2}}$$

where $\rho \leq 1$ and we set $K_{i-,1} \leq K_{i-,2}$. Finally, solving for $\rho$, we get:

$$\rho = \frac{\sqrt{K_{i-,2} - K_{i-,1}}}{\sqrt{K_{i-,2} - K_{i-,1}} + \sqrt{2\left(K_{i-,2} - K_{i-,1}\right)K_{i-,1}K_{i-,2}}} = \frac{1}{1+\sqrt{2K_{i-,1}K_{i-,2}}}.$$

In conclusion, we prove that the maximum difference between the estimand weights, and accordingly the maximum amount of bias, occurs when the ICC is equivalent to:

$$\rho = \frac{1}{1 + \sqrt{2K_{i-,1}K_{i-,2}}}.$$

## A.3.2 Derivation of $P(u = 1)$ for maximum bias

Here, we include the derivations for the $P(u = 1)$ value that yields maximum bias in the EMEw estimator. As stated in Section 5 of the main paper, the EMEw estimator can be interpreted as a weighted average of the cluster-specific cATE estimands, with weights $\lambda_{EMEw}$. We assume there are two subpopulations $u = 1,2$ with fixed cluster-period sizes $K_{i-,1}$ and $K_{i-,2}$ and corresponding weights $\lambda_{EMEw,1}$ and $\lambda_{EMEw,2}$.

Assuming that subpopulations $u = 1$ and 2 have corresponding treatment effects $\delta_1$ and $\delta_2$, the cATE is then equivalent to $P(u = 1)\delta_1 + P(u = 2)\delta_2$. Accordingly, the bias is the difference between the estimator and the estimand:

$$\begin{aligned} Bias &= \left(P(u = 1)\lambda_{EMEw,1}\delta_1 + P(u = 2)\lambda_{EMEW,2}\delta_2\right) - \left(P(u = 1)\delta_1 + P(u = 2)\delta_2\right) \\ &= P(u = 1)\left(\lambda_{EMEw,1} - 1\right)\delta_1 + \left(1 - P(u = 1)\right)\left(\lambda_{EMEW,2} - 1\right)\delta_2 \end{aligned}$$

where:

$$\left(\lambda_{EMEw,1} - 1\right) = \frac{\left(1 - P(u = 1)\right)\left(\frac{1 + \left(K_{i-,1} - 1\right)\rho}{1 + \left(2K_{i-,1} - 1\right)\rho} - \frac{1 + \left(K_{i-,2} - 1\right)\rho}{1 + \left(2K_{i-,2} - 1\right)\rho}\right)}{P(u = 1)\left(\frac{1 + \left(K_{i-,1} - 1\right)\rho}{1 + \left(2K_{i-,1} - 1\right)\rho}\right) + \left(1 - P(u = 1)\right)\left(\frac{1 + \left(K_{i-,2} - 1\right)\rho}{1 + \left(2K_{i-,2} - 1\right)\rho}\right)}$$

and:

$$\left(\lambda_{EMEW,2} - 1\right) = \frac{P(u = 1)\left(\frac{1 + \left(K_{i-,2} - 1\right)\rho}{1 + \left(2K_{i-,2} - 1\right)\rho} - \frac{1 + \left(K_{i-,1} - 1\right)\rho}{1 + \left(2K_{i-,1} - 1\right)\rho}\right)}{P(u = 1)\left(\frac{1 + \left(K_{i-,1} - 1\right)\rho}{1 + \left(2K_{i-,1} - 1\right)\rho}\right) + \left(1 - P(u = 1)\right)\left(\frac{1 + \left(K_{i-,2} - 1\right)\rho}{1 + \left(2K_{i-,2} - 1\right)\rho}\right)}$$

Altogether, the bias can be written as:

$$Bias = \frac{P(u = 1)\left(1 - P(u = 1)\right)\left(\frac{1 + \left(K_{i-,2} - 1\right)\rho}{1 + \left(2K_{i-,2} - 1\right)\rho} - \frac{1 + \left(K_{i-,1} - 1\right)\rho}{1 + \left(2K_{i-,1} - 1\right)\rho}\right)}{P(u = 1)\left(\frac{1 + \left(K_{i-,1} - 1\right)\rho}{1 + \left(2K_{i-,1} - 1\right)\rho}\right) + \left(1 - P(u = 1)\right)\left(\frac{1 + \left(K_{i-,2} - 1\right)\rho}{1 + \left(2K_{i-,2} - 1\right)\rho}\right)}(\delta_1 - \delta_2).$$

The bias is then maximized for values of $P(u = 1)$ where $\frac{dln(Bias)}{dP(u=1)} = 0$. We maximize the log bias $\ln(Bias)$ to make calculations more straightforward. Accordingly:

$$\ln(Bias) = \ln\big(P(u=1)\big) + \ln\big(1 - P(u=1)\big) + \ln\left(\frac{1+\big(K_{i-,2}-1\big)\rho}{1+\big(2K_{i-,2}-1\big)\rho} - \frac{1+\big(K_{i-,1}-1\big)\rho}{1+\big(2K_{i-,1}-1\big)\rho}\right) + \ln(\delta_1 - \delta_2) - \ln\left(P(u=1)\left(\frac{1+\big(K_{i-,1}-1\big)\rho}{1+\big(2K_{i-,1}-1\big)\rho}\right) + \big(1-P(u=1)\big)\left(\frac{1+\big(K_{i-,2}-1\big)\rho}{1+\big(2K_{i-,2}-1\big)\rho}\right)\right)$$

and:

$$\frac{d\ln(Bias)}{dP(u=1)} = \frac{1}{P(u=1)} - \frac{1}{1-P(u=1)} - \frac{\left(\frac{1+\big(K_{i-,1}-1\big)\rho}{1+\big(2K_{i-,1}-1\big)\rho} - \frac{1+\big(K_{i-,2}-1\big)\rho}{1+\big(2K_{i-,2}-1\big)\rho}\right)}{P(u=1)\left(\frac{1+\big(K_{i-,1}-1\big)\rho}{1+\big(2K_{i-,1}-1\big)\rho}\right) + \big(1-P(u=1)\big)\left(\frac{1+\big(K_{i-,2}-1\big)\rho}{1+\big(2K_{i-,2}-1\big)\rho}\right)}$$

which we set $= 0$. Solving for $P(u=1)$:

$$\frac{1}{P(u=1)} - \frac{1}{1-P(u=1)} = \frac{\left(\frac{1+\big(K_{i-,1}-1\big)\rho}{1+\big(2K_{i-,1}-1\big)\rho} - \frac{1+\big(K_{i-,2}-1\big)\rho}{1+\big(2K_{i-,2}-1\big)\rho}\right)}{P(u=1)\left(\frac{1+\big(K_{i-,1}-1\big)\rho}{1+\big(2K_{i-,1}-1\big)\rho}\right) + \big(1-P(u=1)\big)\left(\frac{1+\big(K_{i-,2}-1\big)\rho}{1+\big(2K_{i-,2}-1\big)\rho}\right)}$$

$$\frac{1-2P(u=1)}{P(u=1)\big(1-P(u=1)\big)} = \frac{\left(\frac{1+\big(K_{i-,1}-1\big)\rho}{1+\big(2K_{i-,1}-1\big)\rho} - \frac{1+\big(K_{i-,2}-1\big)\rho}{1+\big(2K_{i-,2}-1\big)\rho}\right)}{P(u=1)\left(\frac{1+\big(K_{i-,1}-1\big)\rho}{1+\big(2K_{i-,1}-1\big)\rho}\right) + \big(1-P(u=1)\big)\left(\frac{1+\big(K_{i-,2}-1\big)\rho}{1+\big(2K_{i-,2}-1\big)\rho}\right)}$$

$$P(u=1)\big(1-P(u=1)\big)\left(\frac{1+\big(K_{i-,1}-1\big)\rho}{1+\big(2K_{i-,1}-1\big)\rho} - \frac{1+\big(K_{i-,2}-1\big)\rho}{1+\big(2K_{i-,2}-1\big)\rho}\right) - \big(1-2P(u=1)\big)\left(P(u=1)\left(\frac{1+\big(K_{i-,1}-1\big)\rho}{1+\big(2K_{i-,1}-1\big)\rho}\right) + \big(1-P(u=1)\big)\left(\frac{1+\big(K_{i-,2}-1\big)\rho}{1+\big(2K_{i-,2}-1\big)\rho}\right)\right) = 0$$

$$P(u=1)^2\left(\frac{1+(K_{i-,1}-1)\rho}{1+(2K_{i-,1}-1)\rho}\right)-\left(\frac{1+(K_{i-,2}-1)\rho}{1+(2K_{i-,2}-1)\rho}\right)+2P(u=1)\left(\frac{1+(K_{i-,2}-1)\rho}{1+(2K_{i-,2}-1)\rho}\right)$$
$$-P(u=1)^2\left(\frac{1+(K_{i-,2}-1)\rho}{1+(2K_{i-,2}-1)\rho}\right)=0$$

$$P(u=1)^2\left(\frac{1+(K_{i-,1}-1)\rho}{1+(2K_{i-,1}-1)\rho}\right)-\left(1-P(u=1)\right)^2\left(\frac{1+(K_{i-,2}-1)\rho}{1+(2K_{i-,2}-1)\rho}\right)=0$$

$$P(u=1)^2\left(\frac{1+(K_{i-,1}-1)\rho}{1+(2K_{i-,1}-1)\rho}\right)=\left(1-P(u=1)\right)^2\left(\frac{1+(K_{i-,2}-1)\rho}{1+(2K_{i-,2}-1)\rho}\right)$$

$$P(u=1)\sqrt{\frac{1+(K_{i-,1}-1)\rho}{1+(2K_{i-,1}-1)\rho}}=\left(1-P(u=1)\right)\sqrt{\frac{1+(K_{i-,2}-1)\rho}{1+(2K_{i-,2}-1)\rho}}$$

Finally, solving for $P(u=1)$, we get:

$$P(u=1)=\frac{\sqrt{\frac{1+(K_{i-,2}-1)\rho}{1+(2K_{i-,2}-1)\rho}}}{\sqrt{\frac{1+(K_{i-,1}-1)\rho}{1+(2K_{i-,1}-1)\rho}}+\sqrt{\frac{1+(K_{i-,2}-1)\rho}{1+(2K_{i-,2}-1)\rho}}}=\frac{\sqrt{\lambda_{EMEw,2}}}{\sqrt{\lambda_{EMEw,1}}+\sqrt{\lambda_{EMEw,2}}}.$$

In conclusion, we prove that the maximum bias occurs when the probability of belonging to subpopulation $u=1$ is equivalent to:

$$P(s=1)=\frac{\sqrt{\lambda_{EMEw,2}}}{\sqrt{\lambda_{EMEw,1}}+\sqrt{\lambda_{EMEw,2}}}$$

with $P(u=2)=1-P(u=1)$. Overall this term tends to be approximately $P(u=1)\approx 0.5$ for most values of $K_{i-,1}, K_{i-,2}$, and $\rho$.

### A.3.3 Additional figures for the estimand weight

EMEw weights are plotted for different values of $\zeta=\frac{K_{i-,1}}{K_{i-,2}}$ and $P(u=1)=0.5, 0.9$, or the "optimal" value $\left(P(u=1)=\frac{\sqrt{\lambda_{EMEw,2}}}{\sqrt{\lambda_{EMEw,1}}+\sqrt{\lambda_{EMEw,2}}}\right)$ using the optimal ICC $\left(\rho=\frac{1}{1+\sqrt{2K_{i-,1}K_{i-,2}}}\right)$ to yield the maximum amount of bias. The optimal ICC is plotted in a gray dashed line.

The "optimal" $P(u=1)$ values were $0.42, 0.42, 0.43, 0.46, 0.47$, and $0.49$ for conditions with $\zeta=0.001, 0.002, 0.005, 0.1, 0.2$, and $0.5$, respectively. Notably, the "optimal" $P(u=1)$ are all $\approx 0.5$ and the graph of their corresponding estimand weights $\lambda_{EMEw,s}$ are qualitatively similar to the graphs with $P(u=1)=0.5$.

Intuitively, the maximum amount of bias occurs around scenarios where $P(u=1)=0.5$, since both subpopulations $u=1$ and 2 are equally overweighted and underweighted,

respectively. In contrast, in the graphed scenario where $P(u = 1) = 0.9$, outcomes from subpopulations $s = 1$ are correctly weighted near 1, whereas outcomes from subpopulation $u = 2$ are more drastically underweighted. However, since subpopulation $u = 1$ makes up 90% of the sampled clusters, the estimate ends up being more correctly weighted and less biased for the cATE estimand as a result.

Overall, we observe that the maximum difference between the estimand weights $\lambda_{EMEw,u}$ increases with larger values of $\zeta$. However, graphing up to an extreme value of $\zeta = 0.001$ with the optimal $P(u = 1)$ and $\rho$ to maximize the bias still yielded fairly conservative values for the estimand weights.

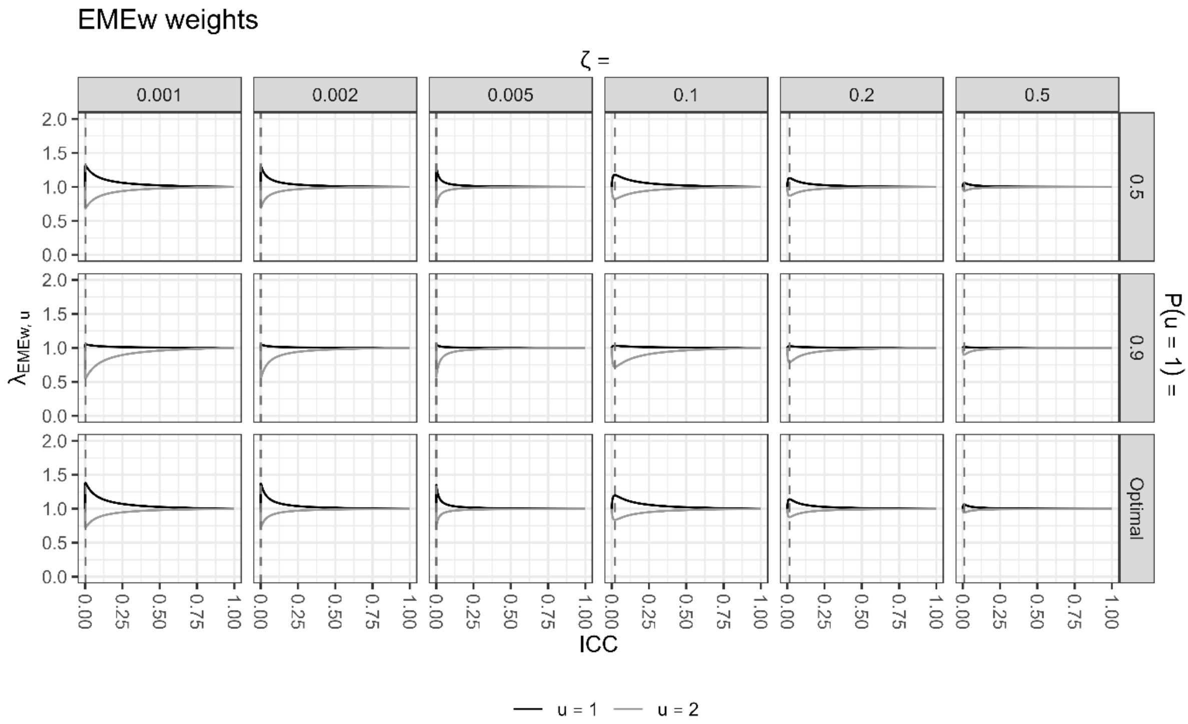

## A.4 Additional simulations

### A.4.1 Simulation results for the cluster robust variance estimator

Simulation results with the bias-reduced linearization (BRL) and Sandwich variance estimators. The bias-reduced linearization method was not implemented with the weighted mixed-effects models (EMEw, NEMEw) due to the lack of compatibility between *WeMix* and *clubSandwich*. Sandwich estimators for IEE, FE, EME, NEME, IEEw, and FEw estimators use the Liang & Zeger (Liang & Zeger, 1986) sandwich variance estimator (as implemented by *clubSandwich* in R); whereas the EMEw and NEMEw estimators use the Rabe-Hesketh & Skrondal (Rabe-Hesketh & Skrondal, 2006) sandwich variance estimator (as implemented by default with *WeMix* in R). Overall, the bias-reduced linearization, and the two sandwich variance estimators all yielded under coverage of the 95% confidence intervals, with only the jackknife variance estimator yielding nominal coverage.

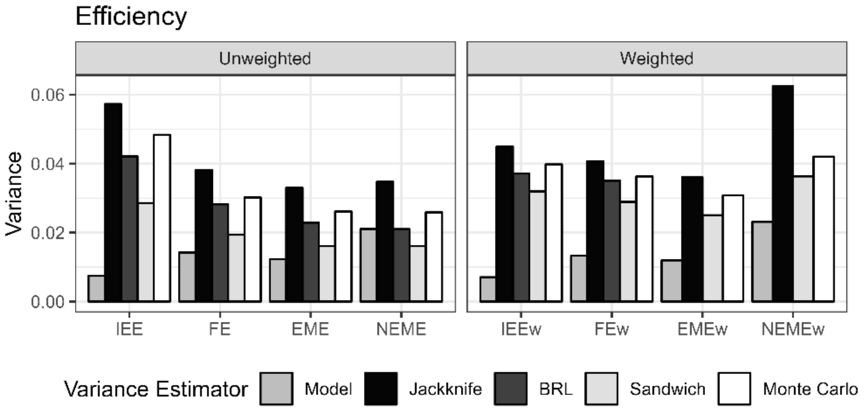


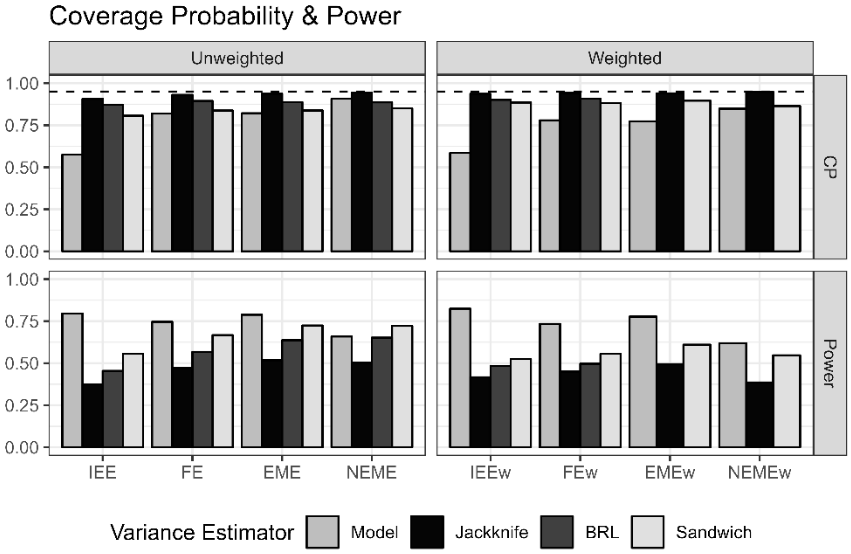


### A.4.2 Additional simulations to evaluate bias

Relative bias results from two additional simulation scenarios. Simulation parameters were set to maximize bias in the EMEw estimator. The two simulation scenarios were simulated with a similar data generating process as described in Section 6 with the same 10

cluster PB-CRT design, but with $E[K_{i-,1}] = 20, E[K_{i-,2}] = 200, \zeta = \frac{E[K_{i-,1}]}{E[K_{i-,2}]} = 0.1$ or $E[K_{i-,1}] = 50, E[K_{i-,2}] = 1000, \zeta = \frac{E[K_{i-,1}]}{E[K_{i-,2}]} = 0.05$. Both scenarios are simulated with $P(u = 1) = 0.5$, CAC=1, and the corresponding $\rho = \frac{1}{1+\sqrt{2E[K_{i-,1}]E[K_{i-,2}]}}$ to optimize the bias in the EMEw estimator for the cATE estimand.

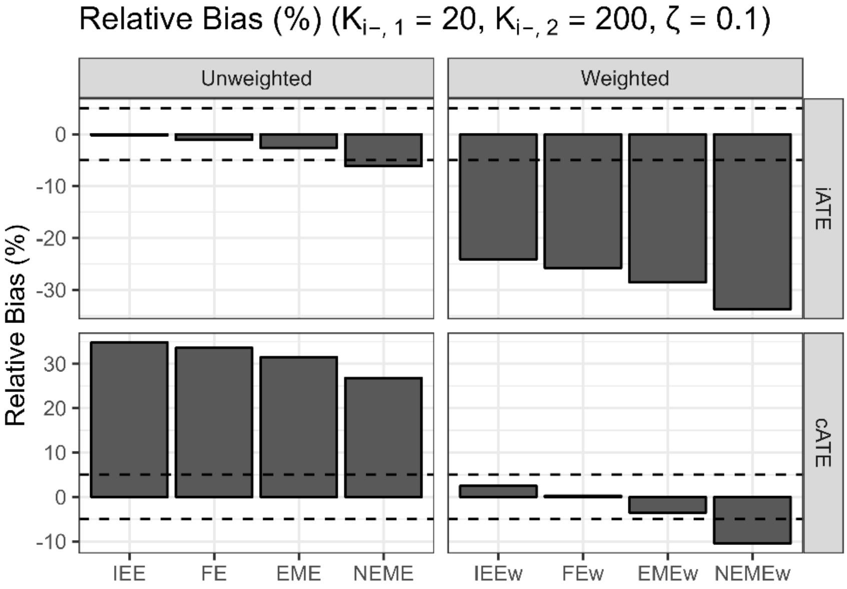


In the first scenario with $\zeta = 0.1$, the EME and EMEw estimator still maintained less than 5% relative bias for the iATE and cATE estimands, respectively. In the second scenario with $\zeta = 0.05$, the EME estimator still maintained less than 5% relative bias for the iATE estimand. The EMEw estimator had slightly over 5% relative bias for the cATE estimand over the 1000 simulation replicates.

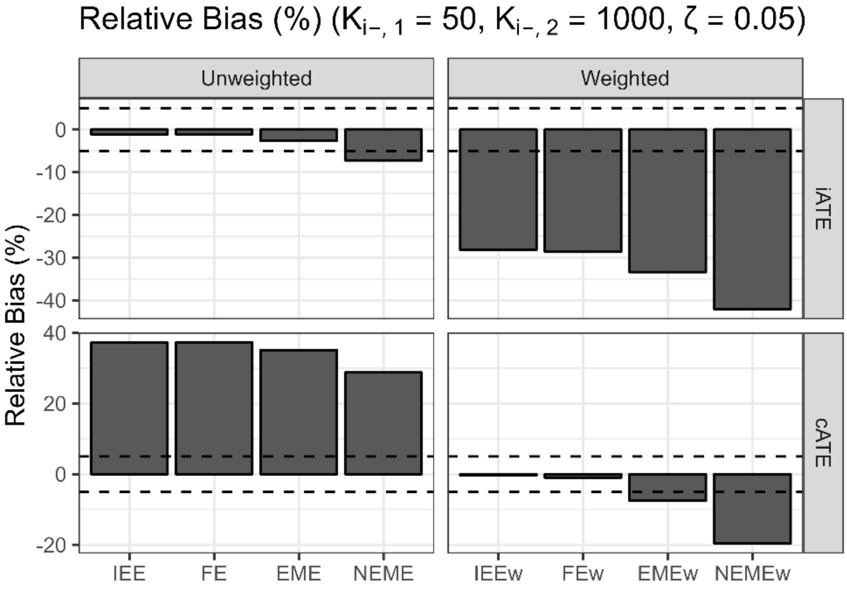


Despite these scenarios being unrealistically tailored to yield biased EMEw estimates for the cATE estimand, the EMEw estimator still remained relatively unbiased. Overall, the

EMEw bias is generally unbiased for the cATE estimand across the explored scenarios, despite not being a theoretically consistent estimator for the cATE in PB-CRTs with informative cluster sizes.

## A.5 Additional information for the case study

Cluster-period cell sizes from the JIAH trial. Cells receiving the intervention are shaded in gray.

| P0 | P1 |
|---|---|
| 48 | 70 |
| 60 | 76 |
| 114 | 81 |
| 47 | 74 |
| 51 | 48 |
| 54 | 17 |
| 75 | 37 |
| 105 | 85 |
| 119 | 73 |
| 69 | 3 |
| 87 | 3 |
| 97 | 92 |
| 132 | 15 |
| 68 | 62 |
| 116 | 94 |
| 92 | 77 |
| 83 | 40 |
| 78 | 22 |
| 101 | 81 |
| 46 | 23 |
| 104 | 93 |
| 138 | 12 |
| 69 | 37 |
| 109 | 52 |
| 50 | 43 |
| 108 | 101 |
| 101 | 5 |
| 69 | 61 |
